# 18th European Symposium on Radiopharmacy and Radiopharmaceuticals: Salzburg, Austria. 7-10 April 2016

**DOI:** 10.1186/s41181-016-0012-6

**Published:** 2016-06-17

**Authors:** V. Radchenko, J. W. Engle, C. Roy, J. Griswold, M. F. Nortier, E. R. Birnbaum, M. Brugh, S. Mirzadeh, K. D. John, M. E. Fassbender, Chuangyan Zhai, Gerben M. Franssen, Milos Petrik, Peter Laverman, Clemens Decristoforo, Ait-Mohand Samia, Dumulon-Perreault Véronique, Guérin Brigitte, D. Summer, A. Kroess, C. Rangger, H. Haas, P. Laverman, F. Gerben, E. von Guggenberg, C. Decristoforo, Cristina Bolzati, Nicola Salvarese, Fiorenzo Refosco, Laura Meléndez-Alafort, Debora Carpanese, Antonio Rosato, Michele Saviano, Annarita Del Gatto, Daniela Comegna, Laura Zaccaro, Emilie Billaud, Muneer Ahamed, Frederik Cleeren, Elnaz Shahbazali, Tim Noël, Volker Hessel, Alfons Verbruggen, Guy Bormans, F. Cleeren, J. Lecina, M. Koole, A. Verbruggen, G. Bormans, B. Lugatoa, S. Stucchia, E. A. Turollaa, L. Giulianoa, S. Toddea, P. Ferraboschib, R. P. Klok, M. P. J. Mooijer, N. H. Hendrikse, A. D. Windhorst, C. Collet, N. Petry, F. Chrétien, G. Karcher, N. Pellegrini-Moïse, S. Lamandé-Langle, Sarah Pfaff, Cecile Philippe, Markus Mitterhauser, Marcus Hacker, Wolfgang Wadsak, François Guérard, Yong-Sok Lee, Sébastien Gouard, Kwamena Baidoo, Cyrille Alliot, Michel Chérel, Martin W. Brechbiel, Jean-François Gestin, K. Lam, C. Chan, R. M. Reilly, Salomé Paillas, John Marshall, Jean-Pierre Pouget, Jane Sosabowski, Emmanuelle Briard, Yves P. Auberson, John Reilly, Mark Healy, David Sykes, Andreas Paulus, Wouter van Marken Lichtenbelt, Felix Mottaghy, Matthias Bauwens, Ann-Christin Baranski, Martin Schäfer, Ulrike Bauder-Wüst, Uwe Haberkorn, Matthias Eder, Klaus Kopka, M. Chaussard, B. Hosten, N. Vignal, V. Tsoupko-Sitnikov, N. Hernio, F. Hontonnou, P. Merlet, J. L. Poyet, L. Sarda-Mantel, N. Rizzo-Padoin, J. Cardinale, M. Schäfer, M. Benešová, U. Bauder-Wüst, O. Seibert, F. Giesel, U. Haberkorn, M. Eder, K. Kopka, Mansour Nematallah, Paquette Michel, Ait-Mohand Samia, Dumulon-Perreault Véronique, Lecomte Roger, Guérin Brigitte, L. Fernandez-Maza, S. Rivera-Marrero, A. Prats Capote, A. Parrado-Gallego, I. Fernandez-Gomez, M. Balcerzyk, M. Sablon-Carrazana, A. Perera-Pintado, D. Merceron-Martinez, E. Acosta-Medina, C. Rodriguez-Tanty, Bala Attili, Muneer Ahamed, Guy Bormans, C. Philippe, M. Zeilinger, T. Scherer, C. Fürnsinn, M. Dumanic, W. Wadsak, M. Hacker, M. Mitterhauser, B. Janssen, D. J. Vugts, G.T. T. Molenaar, U. Funke, P. S. Kruijer, F. Dollé, G. Bormans, A. A. Lammertsma, A. D. Windhorst, Koen Vermeulen, Muneer Ahamed, Michael Schnekenburger, Mathy Froeyen, Dag Erlend Olberg, Marc Diederich, Guy Bormansa, R. M. Raaphorst, G. Luurtsema, A. A. Lammertsma, P. H. Elsinga, A D. Windhorst, Lonneke Rotteveel, Uta Funke, Peter ten Dijke, Harm Jan Bogaard, Adriaan A. Lammertsma, Albert D. Windhorst, Lei Song, Sarah Able, Nadia Falzone, Veerle Kersemans, Katherine Vallis, Davide Carta, Nicola Salvarese, Wiebke Sihver, Feng Gao, Hans Jürgen Pietzsch, Barbara Biondi, Paolo Ruzza, Fiorenzo Refosco, Cristina Bolzati, Roland Haubner, Armin Finkensted, Armin Stegmair, Christine Rangger, Clemens Decristoforo, Heinz Zoller, Irene J. Virgolini, Ivo Pooters, Maartje Lotz, Roel Wierts, Felix Mottaghy, Matthias Bauwens, Sarita Forsback, Bergman Jörgen, Kivelä Riikka, M. Karageorgou, M. Radović, C. Tsoukalas, B. Antic, M. Gazouli, M. Paravatou-Petsotas, S. Xanthopouls, M. Calamiotou, D. Stamopoulos, S. Vranješ-Durić, P. Bouziotis, A. S. Lunev, A. A. Larenkov, K. A. Petrosova, O. E. Klementyeva, G. E. Kodina, O. H. Kvernenes, T. C. H. Adamsen, René Martin, Sebastian Weidlich, Anna-Maria Zerges, Cristiana Gameiro, Neva Lazarova, Marco Müllera, Gert Luurtsema, Michèl de Vries, Michel Ghyoot, Gina van der Woude, Rolf Zijlma, Rudi Dierckx, Hendrikus H. Boersma, Philip H. Elsinga, Fatma Yurt Lambrecht, Ozge Er, Mine Ince, Cıgır Biray Avci, Cumhur Gunduz, Fatma Aslihan Sarı, Kasim Ocakoglu, Ozge Er, Onur Alp Ersoz, Fatma Yurt Lambrecht, Mine Ince, Cagla Kayabasi, Cumhur Gunduz, Torsten Kniess, Sebastian Meister, Steffen Fischer, Jörg Steinbach, Rabia Ashfaq, Saeed Iqbal, Atiq-ur-Rehman, Irfan ullah Khan, R. Iglesias-Jerez, Cayero-Otero, L. Martín-Banderas, A. Perera-Pintado, I. Borrego-Dorado, Ines Farinha-Antunes, Chantal Kwizera, Enza Lacivita, Ermelinda Lucente, Mauro Niso, Paola De Giorgio, Roberto Perrone, Nicola A. Colabufo, Philip H. Elsinga, Marcello Leopoldo, V. V. Vaulina, O. S. Fedorova, V. V. Orlovskaja, С. L. Chen, G. Y. Li, F. C. Meng, R. S. Liu, H. E. Wang, R. N. Krasikova, Laura Meléndez-Alafort, Mohamed Abozeid, Guillermina Ferro-Flores, Anna Negri, Michele Bello, Nikolay Uzunov, Martha Paiusco, Juan Esposito, Antonio Rosato, Laura Meléndez-Alafort, Cristina Bolzati, Guillermina Ferro-Flores, Nicola Salvarese, Debora Carpanese, Mohamed Abozeid, Antonio Rosato, Nikolay Uzunov, L. Palmieri, T. Verbrugghen, M. Glassner, R. Hoogenboom, S. Staelens, L. Wyffels, V. V. Orlovskaja, O. F. Kuznetsova, O. S. Fedorova, V. I. Maleev, Yu. N. Belokon, A. Geolchanyan, A. S. Saghyan, L. Mu, R. Schibli, S. M. Ametamey, R. N. Krasikova, Evgeny Revunov, Jonas Malmquist, Peter Johnström, Juno Van Valkenburgh, Dalton Steele, Christer Halldin, Magnus Schou, Samira Osati, Michel Paquette, Simon Beaudoin, Hasrat Ali, Brigitte Guerin, Jeffrey V. Leyton, Johan E. van Lier, V Di Iorio, M. Iori, C. Donati, V. Lanzetta, P. C. Capponi, S. Rubagotti, T. Dreger, F. Kunkel, M. Asti, Chuangyan Zhai, Christine Rangger, Dominik Summer, Hubertus Haas, Clemens Decristoforo, Suphansa Kijprayoon, Ananya Ruangma, Suthatip Ngokpol, Samart Tuamputsha, Ulrike Filp, Anna Pees, Carlotta Taddei, Aleksandra Pekošak, Antony D. Gee, Alex J. Poot, Albert D. Windhorst, Mine Silindir Gunay, A. Yekta Ozer, Suna Erdogan, Ipek Baysal, Denis Guilloteau, Sylvie Chalon, Filippo Galli, Marco Artico, Samanta Taurone, Enrica Bianchi, Bruce D. Weintraub, Mariusz Skudlinski, Alberto Signore, Nicolas Lepareur, Nicolas Noiret, François Hindré, Franck Lacœuille, Eric Benoist, Etienne Garin, F. Trejo-Ballado, E. Zamora-Romo, J. C. Manrique-Arias, H M Gama-Romero, G. Contreras-Castañon, R. G. Tecuapetla-Chantes, M. A. Avila-Rodriguez, H. Kvaternik, D. Hausberger, C. Zink, B. Rumpf, R. M. Aigner, H. Kvaternik, D. Hausberger, B. Rumpf, R. M. Aigner, Drina Janković, Mladen Lakić, Aleksandar Savić, Slavica Ristić, Nadežda Nikolić, Aleksandar Vukadinović, Tibor J. Sabo, Sanja Vranješ-Đurić, S. Vranješ-Đurić, M. Radović, D. Janković, N. Nikolić, G. F. Goya, P. Calatayud, V. Spasojević, B. Antić, David Goblet, Cristiana Gameiro, Neva Lazarova, Cristiana Gameiro, Ian Oxley, Antero Abrunhosa, Vasko Kramer, Maria Vosjan, Arnold Spaans, Kusum Vats, Drishty Satpati, Haladhar D. Sarma, Sharmila Banerjee, W. Wojdowska, D. W. Pawlak, L. J. Parus, P. Garnuszek, R. Mikołajczak, J. Pijarowska-Kruszyna, A. Jaron, A. Kachniarz, B. Malkowski, P. Garnuszek, R. Mikolajczak, Derya Ilem-Ozdemir, Oya Caglayan-Orumlu, Makbule Asikoglu, Derya Ilem-Ozdemir, Oya Caglayan-Orumlu, Makbule Asikoglu, Arponen Eveliina, Helin Semi, Saarinen Timo, Vauhkala Simo, Kokkomäki Esa, Lehikoinen Pertti, Mariarosaria De Simone, Giancarlo Pascali, Ludovica Carzoli, Mauro Quaglierini, Mauro Telleschi, Piero A. Salvadori, Phoebe Lam, Martina Aistleitner, Reinhard Eichinger, Christoph Artner, Surendra Nakka, Hemantha Kumara MC, Mohammed Al-Qahtani, Mohammed Al-Qahtani, Yousif Al-Malki, N. Mambilima, S. M. Rubow, N. Berroterán-Infante, M. Hacker, M. Mitterhauser, W. Wadsak, Uta Funke, Frederik Cleeren, Joan Lecina, Rodrigo Gallardo, Alfons M. Verbruggen, Guy Bormans, Rocío Ramos-Membrive, Ana Brotons, Gemma Quincoces, Laura Inchaurraga, Inés Luis de Redín, Verónica Morán, Berta García-García, Juan Manuel Irache, Iván Peñuelas, M. Trabelsi, M. S. Cooper, Alejandra Abella, Teodomiro Fuente, Antonio Jesús Montellano, Teresa Martínez, Ruben Rabadan, Luis Meseguer-Olmo, P. Lehtiniemi, C. Yim, K. Mikkola, P. Nuutila, O. Solin, E. von Guggenberg, C. Rangger, C. Mair, L. Balogh, Z. Pöstényi, D. Pawlak, R. Mikołajczak, A. Socan, P. Kolenc Peitl, M. Krošelj, C. Rangger, C. Decristoforo, C. Collet, S. Remy, R. Didier, T. Vergote, G. Karcher, N. Véran, D. Pawlak, M. Maurin, P. Garnuszek, U. Karczmarczyk, R. Mikołajczak, Pil Fredericia, Gregory Severin, Torsten Groesser, Ulli Köster, Mikael Jensen, R. Leonte, F. D. Puicea, A. Raicu, E. A. Min, R. Serban, G. Manda, D. Niculae, Marion Zerna, Hanno Schieferstein, Andre Müller, Mathias Berndt, Cheng-Bin Yim, Kirsi Mikkola, Pirjo Nuutila, Olof Solin, D. Seifert, J. Ráliš, O. Lebeda, Svetlana V. Selivanova, Helena Senta, Éric Lavallée, Lyne Caouette, Éric Turcotte, Roger Lecomte, Marina Zdraveska Kochovska, Emilija Janjevik Ivanovska, Vesna Spasic Jokic, Darinka Gjorgieva Ackova, Katarina Smilkov, Petre Makreski, Trajče Stafilov, Emilija Janevik-Ivanovska, Aschalew Alemu, Joel Munene Muchira, David Mwanza Wanjeh, Emilija Janevik-Ivanovska, Emilija Janevik-Ivanovska, Zoran Zdravev, Uday Bhonsle, Osso Júnior João Alberto, Adriano Duatti, Bistra Angelovska, Zdenka Stojanovska, Zorica Arsova Sarafinovska, Darko Bosnakovski, Darinka Gorgieva-Ackova, Katarina Smilkov, Elena Drakalska, Meera Venkatesh, Rubin Gulaboski, Didier J. Colin, James A. H. Inkster, Stéphane Germain, Yann Seimbille

**Affiliations:** 1 Los Alamos National Laboratory, Los Alamos, New Mexico USA; 2 Oak Ridge National Laboratory, Oak Ridge, Tennessee USA; 3 Department of Nuclear Medicine, Medical University Innsbruck, Innsbruck, Austria; 4 Department of Radiology & Nuclear Medicine, Radboud University Medical Center, Nijmegen, The Netherlands; 5 Institute of Molecular and Translational Medicine, Faculty of Medicine and Dentistry, Palacky University, Olomouc, Czech Republic; 6 Département de Médecine Nucléaire et Radiobiologie, Faculté de Médecine et Sciences de la Santé, Université de Sherbrooke, Sherbrooke, QC Canada J1H5N4; 7 Centre d’Imagerie Moléculaire de Sherbrooke (CIMS), CR-CHUS, Sherbrooke, QC Canada J1H5N4,; 8 Department of Nuclear Medicine, Medical University Innsbruck, Innsbruck, Austria; 9 Division of Molecular Biology/Biocenter, Medical University Innsbruck, Innsbruck, Austria; 10 Department of Nuclear Medicine, Radboud University Medical Center, Nijmegen, The Netherlands; 11 IENI-CNR, Padua, Italy; 12 DiSCOG-University of Padua, Padua, Italy; 13 IOV Padua, Padua, Italy; 14 IC-CNR, Bari, Italy; 15 IBB-CNR, Naples, Italy; 16 Laboratory of Radiopharmacy, KU Leuven, Leuven, Belgium; 17 Micro Flow Chemistry & Process Technology, Chemical Engineering and Chemistry Department, TU Eindhoven, Eindhoven, The Netherlands; 18 Laboratory for Radiopharmacy, University of Leuven, Leuven, Belgium; 19 Department of Nuclear Medicine and Molecular Imaging, University of Leuven, Leuven, Belgium; 20 Department of Medicine and Surgery, Tecnomed Foundation, University of Milano-Bicocca, Milan, Italy; 21 Dipartimento di Biotecnologie Mediche e Medicina Traslazionale, Università degli Studi di Milano, Milan, Italy; 22 Department of Radiology & Nuclear Medicine, VU University Medical Center, Amsterdam, The Netherlands; 23 Department of Clinical Pharmacology & Pharmacy, VU University Medical Center, Amsterdam, The Netherlands; 24 Université de Lorraine, F-54500 Vandoeuvre les Nancy, France; 25 NancycloTEP, Plateforme d’imagerie expérimentale, 54500 Vandoeuvre les Nancy, France; 26 CNRS, UMR 7565 SRSMC, F-54506 Vandoeuvre les Nancy, France; 27 CHU de Nancy-Brabois, F-54511 Vandoeuvre les Nancy, France; 28 Department of Biomedical Imaging and Image-guided Therapy, Division of Nuclear Medicine, Medical University of Vienna, Vienna, Austria; 29 Department of Inorganic Chemistry, University of Vienna, Vienna, Austria; 30 LBI for Applied Diagnostics, Vienna, Austria; 31 Centre de Recherche en Cancérologie Nantes-Angers (CRCNA), Unité INSERM 892 - CNRS 6299, Nantes, 44007 France; 32 Center for Molecular Modeling, Division of Computational Bioscience, Center for Information Technology, National Institutes of Health, Bethesda, Maryland 20892 USA; 33 Radioimmune & Inorganic Chemistry Section, Radiation Oncology Branch, National Cancer Institute, National Institutes of Health, Bethesda, Maryland 20892 USA; 34 Arronax GIP, Nantes, 44817 France; 35 Department of Pharmaceutical Sciences, University of Toronto, Toronto, Canada; 36 Department of Medical Imaging, University of Toronto, Toronto, Canada; 37 Toronto General Research Institute, University Health Network, Toronto, Canada; 38 Centre for Molecular Oncology, Barts Cancer Institute, Charterhouse Square, Queen Mary University of London, London, UK; 39 Centre for Tumour Biology, Barts Cancer Institute, Charterhouse Square, Queen Mary University of London, London, UK; 40 Institut de Recherche en Cancérologie de Montpellier, Inserm U1194, Montpellier, France; 41 Novartis Institutes for Biomedical Research, Basel, Switzerland; 42 Novartis Institutes for Biomedical Research, Cambridge, USA; 43 University of Nottingham, Nottingham, UK; 44 Research School NUTRIM, Maastricht University, Maastricht, Netherlands; 45 Division of Nuclear Medicine, Uniklinikum Aachen, Aachen, Germany; 46 Department of Medical Imaging, Division of Nuclear Medicine, MUMC, Maastricht, Netherlands; 47 Division of Radiopharmaceutical Chemistry, German Cancer Research Center (dkfz), Heidelberg, Germany; 48 Department of Nuclear Medicine, University of Heidelberg, Heidelberg, Germany; 49 Unité Claude Kellershohn, IUH, Hôpital Saint-Louis, Paris, F-75010 France; 50 GH Saint-Louis Lariboisière F. Widal AP-HP, Paris, F-75010 France; 51 Inserm U1160, Hôpital Saint-Louis, Paris, F-75010 France; 52 Université Paris Descartes, Faculté de Pharmacie, Paris, F-75006 France; 53 Université Paris Diderot, Faculté de médecine, Paris, F-75010 France; 54 DKFZ, Division of Radiopharmaceutical Chemistry, Heidelberg, Germany; 55 DKFZ, Clinical Cooperation Unit Nuclear Medicine, Heidelberg, Germany; 56 Département de médecine nucléaire et radiobiologie, Faculté de médecine et sciences de la santé, Université de Sherbrooke, Sherbrooke, QC Canada J1H5N4; 57 Centre d’Imagerie Moléculaire de Sherbrooke (CIMS), CR-CHUS, Sherbrooke, QC Canada J1H5N4; 58 Centro Nacional de Aceleradores. Universidad de Sevilla, CSIC, Junta de Andalucia, Sevilla, Spain; 59 Centro de Neurociencias (CNEURO), La Habana, Cuba; 60 Centro de Isótopos (CENTIS), Mayabeque, Cuba; 61 Centro de Estudios Avanzados de Cuba (CEAC), La Habana, Cuba; 62 Laboratory for Radiopharmacy, Department of Pharmaceutical and Pharmacological Sciences, KU Leuven, Leuven, Belgium; 63 Department of Biomedical Imaging and Image-guided Therapy, Division of Nuclear Medicine, Radiopharmacy and Experimental Nuclear Medicine, Medical University of Vienna, Vienna, Austria; 64 Department of Medicine III, Division of Endocrinology and Metabolism, Medical University of Vienna, Vienna, Austria; 65 Ludwig Boltzmann Institute for Applied Diagnostics, Vienna, Austria; 66 Department of Radiology & Nuclear Medicine, Neuroscience Campus Amsterdam, VU University Medical Center, Amsterdam, The Netherlands; 67 BV Cyclotron VU, Amsterdam, The Netherlands; 68 CEA, Institut d’Imagerie BioMédicale, Service Hospitalier Frédéric Joliot, Orsay, France; 69 Laboratory for Radiopharmacy, Department of Pharmaceutical and Pharmacological Sciences, KU Leuven, Leuven, Belgium; 70 Lab of Radiopharmacy, KU Leuven, Leuven, Belgium; 71 Laboratoire de Biologie Moléculaire et Cellulaire du Cancer, Luxembourg, Grand Duchy of Luxembourg; 72 Laboratory for Medicinal Chemistry, Rega Institute of Medical Research, KU Leuven, Leuven, Belgium; 73 School of Pharmacy, University of Oslo and Norwegian Medical Cyclotron Centre, Oslo, Norway; 74 Department of Radiology & Nuclear Medicine, Neuroscience Campus Amsterdam, VU University Medical Center, Amsterdam, The Netherlands; 75 Department of Nuclear Medicine and Molecular Imaging, University Medical Center Groningen, University of Groningen, Groningen, The Netherlands; 76 Department of Radiology & Nuclear medicine, Institute for Cardiovascular Research, VU University Medical Center, Amsterdam, The Netherlands; 77 Department of Pulmonary Medicine, Institute for Cardiovascular Research, VU University Medical Center, Amsterdam, The Netherlands; 78 Department of Molecular Cell Biology, Leiden University Medical Center, Leiden, The Netherlands; 79 CR-UK/MRC Oxford Institute for Radiation Oncology, Department of Oncology, University of Oxford, Oxford, UK; 80 DSF, University of Padua, Via Marzolo 5, 35131 Padova, Italy; 81 IENI-CNR, Corso Stati Uniti 4, 35127 Padova, Italy; 82 Institute of Radiopharmaceutical Cancer Research, HZDR, Bautzner Landstrasse 400, 01328 Dresden, Germany; 83 ICB-CNR, Via Marzolo 1, 35131 Padova, Italy; 84 Department of Nuclear Medicine, Medical University of Innsbruck, Innsbruck, Austria; 85 Department of Internal Medicine, Medical University of Innsbruck, Innsbruck, Austria; 86 FH Gesundheit/University of Applied Sciences Tyrol, Innsbruck, Austria; 87 Department of Radiology and Nuclear Medicine, MUMC+, Maastricht, The Netherlands; 88 Nuclear Medicine, Uniklinikum Aachen, Aachen, Germany; 89 Research School NUTRIM, Maastricht University, Maastricht, The Netherlands; 90 Radiopharmaceutical Chemistry Laboratory, Turku PET Centre, University of Turku, Turku, Finland; 91 Turku University Hospital, Hospital Pharmacy, Turku, Finland; 92 Radiochemical Studies Laboratory, INRASTES, NCSR “Demokritos”, Athens, Greece; 93 Vinča” Institute of Nuclear Sciences, Laboratory for Radioisotopes, University of Belgrade, Belgrade, Serbia; 94 Department of Basic Medical Science, Laboratory of Biology, School of Medicine, NKUA, Athens, Greece; 95 Department of Solid State Physics, NKUA, Athens, Greece; 96 INN, NCSR “Demokritos”, Athens, Greece; 97 Burnasyan Federal Medical Biophysical Center of FMBA Russia, Moscow, Russia; 98 Moscow State Academy of Veterinary Medicine and Biotechnology, Moscow, Russia; 99 Centre for Nuclear Medicine/PET, Department of Radiology, Haukeland University Hospital, Jonas Lies Vei 65, 5021 Bergen, Norway; 100 Department of Chemistry, University of Bergen, Allégaten 41, 5007 Bergen, Norway; 101 ABX GmbH, Radeberg, Germany; 102 IBA RadioPharma Solutions, Louvain-La-Neuve, Belgium; 103 Department of Nuclear Medicine and Molecular Imaging, University Medical Center Groningen, University of Groningen, Groningen, The Netherlands; 104 IBA RadioPharma Solutions, Louvain-la-Neuve, Belgium; 105 Department of Nuclear Applications, Institute of Nuclear Science, Ege University, Bornova, 35100 Izmir Turkey; 106 Department of Energy Systems Engineering, Faculty of Technology, Mersin University, TR-33480 Tarsus, Mersin Turkey; 107 Department of Medical Biology, Faculty of Medicine, Ege University, Bornova, 35100 Izmir Turkey; 108 Advanced Technology Research & Application Center, Mersin University, Ciftlikkoy Campus, TR-33343 Yenisehir, Mersin Turkey; 109 Advanced Technology Research & Application Center, Mersin University, Ciftlikkoy Campus, TR-33343 Yenisehir, Mersin Turkey; 110 Department of Energy Systems Engineering, Faculty of Technology, Mersin University, TR-33480 Tarsus, Mersin Turkey; 111 Department of Nuclear Applications, Institute of Nuclear Science, Ege University, Bornova, 35100 Izmir Turkey; 112 Department of Medical Biology, Faculty of Medicine, Ege University, Bornova, 35100 Izmir Turkey; 113 Helmholtz-Zentrum Dresden-Rossendorf, Institute of Radiopharmaceutical Cancer Research, 01314 Dresden, Germany; 114 Nuclear Medicine Department, Shaukat Khanum Cancer Hospital and Research Centre, Lahore, Pakistan; 115 Forman Christian College, Lahore, Pakistan; 116 Institute of Nuclear medicine and Oncology, Lahore, Pakistan; 117 Servicio de Medicina Nuclear, Hospital Universitario Virgen del Rocío, Avda Manuel Siurot s/n., 41013 Seville, Spain; 118 Dpt. Farmacia y Tecnología Farmacéutica, Facultad de Farmacia, Universidad de Sevilla, c/Prof. Gracía González n°2, 41012 Seville, Spain; 119 Dirección de Investigaciones Clínicas, Centro de Isotopos, 34 No. 4501 e/45 y 47, Kohly, Playa, La Habana CP 11300 Cuba; 120 Dipartimento di Farmacia – Scienze del Farmaco, Università degli Studi di Bari “Aldo Moro”, Bari, Italy; 121 Department of Nuclear Medicine and Molecular Imaging, University Medical Center Groningen, University of Groningen, Groningen, The Netherlands; 122 BIOFORDRUG s.r.l., Spin-off, Università degli Studi di Bari “Aldo Moro”, Bari, Italy; 123 N.P. Bechtereva Institute of Human Brain, Russian Academy of Science, Saint-Petersburg, Russian Federation; 124 Department of Biomedical Imaging and Radiological Sciences, National Yang-Ming University Taipei, Taipei, Taiwan; 125 National PET/Cyclotron Center, Veterans General Hospital, Taipei, Taiwan; 126 DiSCOG-University of Padua, Padua, Italy; 127 ININ, Ocoyoacac, México; 128 IOV Padua, Padua, Italy; 129 DFA-University of Padua, Padua, Italy; 130 Faculty of Natural Sciences, University of Shumen, Shumen, Bulgaria; 131 INFN-LNL, Legnaro, Italy; 132 DiSCOG-University of Padua, Padua, Italy; 133 IENI-CNR, Padua, Italy; 134 ININ, Ocoyoacac, México; 135 IOV Padua, Padua, Italy; 136 Faculty of Natural Sciences, University of Shumen, Shumen, Bulgaria; 137 Molecular Imaging Center Antwerp, University of Antwerp, Antwerp, Belgium; 138 Department of Nuclear Medicine, Antwerp University Hospital, Antwerp, Belgium; 139 Supramolecular Chemistry, Ghent University, Ghent, Belgium; 140 N.P. Bechtereva Institute of Human Brain, RAS, St.-Petersburg, Russia; 141 A.N. Nesmeyanov Institute of Organoelement Compounds, RAS, Moscow, Russia; 142 SPC Armbiotechnology NAS, Yerevan, Republic of Armenia; 143 Center for Radiopharmaceutical Sciences of ETH, PSI and USZ, Zurich, Switzerland; 144 Department of Clinical Neuroscience, Centre for Psychiatric Research, Karolinska Institutet, Stockholm, Sweden; 145 AstraZeneca Translational Science Centre, Stockholm, Sweden; 146 Department of Medical and Molecular Pharmacology, Ahmanson Translational Imaging Division, University of California, Los Angeles, USA; 147 Département de médecine nucléaire et radiobiologie, Faculté de médecine et sciences de la santé, Université de Sherbrooke, Sherbrooke, QC Canada J1H5N4; 148 Centre d’Imagerie Moléculaire de Sherbrooke (CIMS), CR-CHUS, Sherbrooke, QC Canada J1H5N4; 149 Radiopharmacy Nuclear Medicine Unit, IRST IRCCS, Meldola (FC), Italy; 150 Nuclear Medicine Unit, IRCCS-Arcispedale Santa Maria Nuova, Reggio Emilia, Italy; 151 Eckert & Ziegler Eurotope GmbH, Berlin, Germany; 152 Department of Nuclear Medicine, Medical University Innsbruck, Innsbruck, Austria; 153 Division of Molecular Biology, Medical University Innsbruck, Innsbruck, Austria; 154 Oncology Imaging & Nuclear Medicine Department, Wattanosoth Hospital, Bangkok Hospital at Headquarter, Bangkok, Thailand; 155 Radiology and Nuclear Medicine, VU University Medical Center, Amsterdam, The Netherlands; 156 Division of Imaging Sciences and Biomedical Engineering, King’s College, London, UK; 157 Department of Radiopharmacy, Faculty of Pharmacy, Hacettepe University, 06100 Ankara, Turkey; 158 UMR INSERM U930, Université François Rabelais de Tours, Tours, France; 159 Department of Biochemistry, Faculty of Pharmacy, Hacettepe University, 06100 Ankara, Turkey; 160 Nuclear Medicine Unit, Department of Medical-Surgical Sciences and of Translational Medicine, Faculty of Medicine and Psychology, “Sapienza” University of Rome, Rome, Italy; 161 Department of Sensory Organs, Sapienza University of Rome, Rome, Italy; 162 Trophogen Inc., Rockville, MD USA; 163 Centre Eugene Marquis, Nuclear Medicine Department, INSERM UMR-S 991, 35042 Rennes, France; 164 ENSCR, CNRS UMR 6226, 35708, Rennes, France; 165 University of Angers, INSERM UMR-S 1066 MINT, 49100 Angers, France; 166 University of Angers, SFR ICAT, PRIMEX, 49100 Angers, France; 167 Université Paul Sabatier, SPCMIB, UMR CNRS 5068, 31062 Toulouse, France; 168 Unidad Radiofarmacia-Ciclotrón, Facultad de Medicina, Universidad Nacional Autónoma de México, México, D.F. 04510 México; 169 Division of Nuclear Medicine, Department of Radiology, Medical University of Graz, Graz, Austria; 170 Division of Nuclear Medicine, Department of Radiology, Medical University of Graz, Graz, Austria; 171 Laboratory for radioisotopes, Vinča Institute of Nuclear Sciences, University of Belgrade, Belgrade, Serbia; 172 Chair of General and Inorganic Chemistry, Faculty of Chemistry, University of Belgrade, Belgrade, Serbia; 173 Biomedical Research Center, R&D Institute, Galenika a.d., Belgrade, Serbia; 174 Laboratory for radioisotopes, “Vinča” Institute of Nuclear Sciences, University of Belgrade, Belgrade, Serbia; 175 Instituto de Nanociencia de Aragón (INA), University of Zaragoza, Zaragoza, Spain; 176 IBA, Louvain-la-Neuve, Belgium; 177 IBA SA, Louvain-La-Neuve, Belgium; 178 ICNAS, University of Coimbra, Coimbra, Portugal; 179 PositronPharma S.A., Santigo, Chile; 180 BV Cyclotron VU, Amsterdam, The Netherlands; 181 Radiopharmaceuticals Chemistry Section, Bhabha Atomic Research Centre, Trombay, Mumbai 400085 India; 182 Radiation Biology and Health Science Division, Bhabha Atomic Research Centre, Trombay, Mumbai 400085 India; 183 Radioisotope Centre POLATOM, National Centre for Nuclear Research, Andrzeja Sołtana 7, 05-400 Otwock, Poland; 184 Radioisotope Centre POLATOM, National Centre for Nuclear Research, Otwock, Poland; 185 Department of Nuclear Medicine, Oncology Centre prof. Lukaszczyk Memorial Hospital, Bydgoszcz, Poland; 186 Faculty of Pharmacy, Department of Radiopharmacy, Ege University, Bornova, Izmir, Turkey; 187 Faculty of Pharmacy, Department of Radiopharmacy, Ege University, Bornova, Izmir, Turkey; 188 Radiopharmaceutical Chemistry Laboratory, Turku PET Centre, University of Turku, Turku, Finland; 189 CNR Institute Clinical Physiology Pisa, Pisa, Italy; 190 Life Sciences, Australian Nuclear Science and Technology Organisation, New Illawarra Road, Lucas Heights, Sydney, NSW 2234 Australia; 191 IASON GmbH, Feldkirchnerstraße 4, A-8054 Graz-Seiersberg, Austria; 192 Cyclotron and Radiopharmaceuticals Department, King Faisal Specialist Hospital and Research Center, Riyadh, Saudi Arabia; 193 Cyclotron and Radiopharmaceuticals Department, King Faisal Specialist Hospital and Research Center, Riyadh, Saudi Arabia; 194 University Teaching Hospital, Lusaka, Zambia; 195 Stellenbosch University, Stellenbosch, South Africa; 196 Division of Nuclear Medicine, Department of Biomedical Imaging and Image-Guided Therapy, Medical University of Vienna, Vienna, Austria; 197 Department of Inorganic Chemistry, University of Vienna, Vienna, Austria; 198 LBI for Applied Diagnostics, Vienna, Austria; 199 Laboratory for Radiopharmacy, Department of Pharmaceutical and Pharmacological Sciences, KU Leuven, Leuven, Belgium; 200 VIB Switch Laboratory, KU Leuven, Leuven, Belgium; 201 Radiopharmacy Unit, Department of Nuclear Medicine, University Clinic of Navarra, Pamplona, Spain; 202 Department of Pharmaceutics and Pharmaceutical Technology, University of Navarra, Pamplona, Spain; 203 Department of Nuclear Medicine, University Clinic of Navarra, Pamplona, Spain; 204 Radiopharmacy Department, Royal Liverpool University Hospital, Prescot Street, Liverpool, L7 8XP UK; 205 Unidad de Radiofarmacia Hospital Clínico Universitario Virgen de la Arrixaca, Madrid, Spain; 206 Servicio de Medicina Nuclear, Clínica Belén, Murcia, Spain; 207 Servicio de Traumatología, Hospital Clínico Universitario Virgen de la Arrixaca, Murcia, Spain; 208 UCAM Universidad Católica San Antonio de Murcia, Murcia, Spain; 209 Turku PET Centre, University of Turku, Kiinamyllynkatu 4-8, FI-20500 Turku, Finland; 210 Turku PET Centre, Åbo Akademi University, Porthansgatan 3, FI-20500 Turku, Finland; 211 Department of Nuclear Medicine, Innsbruck Medical University, Innsbruck, Austria; 212 Radioisotope Centre POLATOM, National Centre for Nuclear Research, Otwock, Poland; 213 “Frédéric Joliot-Curie” National Research Institute for Radiobiology and Radiohygiene (NRIRR), Budapest, Hungary; 214 Department of Nuclear Medicine, University Medical Centre Ljubljana, Ljubljana, Slovenia; 215 University Clinic for Nuclear Medicine, University for Medicine, Innsbruck, Austria; 216 Nancyclotep, Plateforme d’imagerie moléculaire, 54500 Vandoeuvre les Nancy, France; 217 Université de Lorraine, F-54500 Vandoeuvre les Nancy, France; 218 CHRU de Nancy-Brabois, F-54511 Vandoeuvre les Nancy, France; 219 Trasis SA, B-4430 Ans, Belgium; 220 National Centre for Nuclear Research Radioisotope Centre POLATOM, Sołtana 7, 05-400 Otwock, Poland; 221 Hevesy Laboratory, DTU-Nutech, Technical University of Denmark, Roskilde, Denmark; 222 Institut Laue-Langevin, Grenoble, France; 223 Horia Hulubei National Institute for Physics and Nuclear Engineering, Radiopharmaceuticals Research Centre, Magurele, Romania; 224 Politehnica University, Bucharest, Romania; 225 National Institute of Patology Victor Babes, Bucharest, Romania; 226 Piramal Imaging, Berlin, Germany; 227 Turku PET Centre, University of Turku, Turku, Finland; 228 Åbo Akademi University, Turku, Finland; 229 Nuclear Physics Institute of the CAS, v. v. i., Husinec-Řež 130, 250 68 Řež, Czech Republic; 230 Molecular Imaging Centre, CRCHUS, Université de Sherbrooke, Sherbrooke, QC Canada; 231 Institute of pathophysiology and nuclear medicine, Medical faculty, Skopje, Macedonia; 232 Faculty of Medical Sciences, University of “Goce Delcev”, Stip, Macedonia; 233 Faculty of Technical Sciences, Universty of Novi Sad, Novi Sad, Serbia; 234 Department of Pharmacy, Faculty of Medical Sciences, University Goce Delčev, Štip, R. Macedonia; 235 Department of Chemistry, Faculty of Natural Sciences and Mathematics, University “Ss. Cyril and Methodius”, Skopje, R. Macedonia; 236 Faculty of Medicine, Addis Ababa, Ethiopia; 237 Ministry of Health, Nairobi City, Kenya; 238 Faculty of Medical Sciences, University Goce Delčev – Štip, Shtip, R. Macedonia; 239 Faculty of Medical Sciences, Goce Delcev University, Stip, Republic of Macedonia; 240 Faculty of Informatics, Goce Delcev University, Stip, Republic of Macedonia; 241 Radioisotope Products and Radiation Technology Section, Division of Physical and Chemical Sciences, IAEA, Vienna, Austria; 242 Laboratory of Radiopharmaceutical Chemistry, Department of Chemical and Pharmaceutical Sciences, University of Ferrara, Ferrara, Italy; 243 MicroPET/SPECT/CT Imaging Laboratory, Centre for BioMedical Imaging (CIBM), University Hospital of Geneva, Geneva, Switzerland; 244 Cycloton Unit, University Hospital of Geneva, Geneva, Switzerland; 245 Life Sciences Division, TRIUMF, Vancouver, Canada

**Keywords:** Positron Emission Tomography, Single Photon Emission Compute Tomography, Standard Uptake Value, Positron Emission Tomography Imaging, High Pressure Liquid Chromatography

## Abstract

OP03 Selective extraction of medically-related radionuclides from proton-irradiated thorium targets

V. Radchenko, J.W. Engle, C. Roy, J. Griswold, M.F. Nortier, E.R. Birnbaum, M. Brugh, S. Mirzadeh, K. D. John, M.E. Fassbender

OP04 Comparison of [68Ga]FSC(succ-RGD)3 and [68Ga]NODAGA-RGD for PET imaging of αvβ3 integrin expression

Chuangyan Zhai, Gerben M. Franssen, Milos Petrik, Peter Laverman, Clemens Decristoforo

OP05 A new NPY-Y1R targeting peptide for breast cancer PET imaging

Ait-Mohand Samia, Dumulon-Perreault Véronique, Guérin Brigitte

OP06 The influence of multivalency on CCK 2 receptor targeting

D. Summer, A. Kroess, C. Rangger, H. Haas, P. Laverman, F. Gerben, E. von Guggenberg, C.Decristoforo

OP07 SPECT Imaging of αvβ3 Expression by [99mTc(N)PNP43]- Bifunctional Chimeric RGD Peptide not Cross-Reacting with αvβ5

Cristina Bolzati, Nicola Salvarese, Fiorenzo Refosco, Laura Meléndez-Alafort, Debora Carpanese, Antonio Rosato, Michele Saviano, Annarita Del Gatto, Daniela Comegna, Laura Zaccaro

OP09 New dienophiles for the inverse-electron-demand Diels-Alder reaction and for pretargeted PET imaging

Emilie Billaud, Muneer Ahamed, Frederik Cleeren, Elnaz Shahbazali, Tim Noël, Volker Hessel, Alfons Verbruggen and Guy Bormans

OP10 New complexing agent for Al18F-labelling of heat-sensitive biomolecules: Synthesis and preclinical evaluation of Al18F-RESCA1-HAS

Cleeren F, Lecina J, Koole M, Verbruggen A and Bormans G

OP11 A novel versatile precursor efficient for F-18 radiolabelling via click-chemistry

B. Lugatoa, S. Stucchia, E.A. Turollaa, L. Giulianoa, S.Toddea, P. Ferraboschib

OP12 A general applicable method to quantify unidentified UV impurities in radiopharmaceuticals

R.P. Klok, M.P.J. Mooijer, N.H. Hendrikse, A.D. Windhorst

OP13 Development of [^18^F]Fluoro-C-glycosides to radiolabel peptides

Collet C., Petry N., Chrétien F., Karcher G., Pellegrini-Moïse N., Lamandé-Langle S.

OP14 A Microfluidic Approach for the 68Ga-labeling of PSMAHBED-CC and NODAGA-RGD

Sarah Pfaff, Cecile Philippe, Markus Mitterhauser, Marcus Hacker, Wolfgang Wadsak

OP16 Surprising reactivity of astatine in the nucleophilic substitution of aryliodonium salts: application to the radiolabeling of antibodies

François Guérard, Yong-Sok Lee, Sébastien Gouard, Kwamena Baidoo, Cyrille Alliot, Michel Chérel, Martin W. Brechbiel, Jean-François Gestin

OP17 ^64^Cu-NOTA-pertuzumab F(ab')_2_ fragments, a second-generation probe for PET imaging of the response of HER2-positive breast cancer to trastuzumab (Herceptin)

Lam K, Chan C, Reilly RM

OP18 Development of radiohalogenated analogues of a avb6-specific peptide for high LET particle emitter targeted radionuclide therapy of cancer

Salomé Paillas, John Marshall, Jean-Pierre Pouget, Jane Sosabowski

OP19 Ligand Specific Efficiency (LSE) as a guide in tracer optimization

Emmanuelle Briard, Yves P. Auberson, John Reilly, Mark Healy, David Sykes

OP23 The radiosynthesis of an 18F-labeled triglyceride, developed to visualize and quantify brown adipose tissue activity

Andreas Paulus, Wouter van Marken Lichtenbelt,Felix Mottaghy, Matthias Bauwens

OP24 Influence of the fluorescent dye on the tumor targeting properties of dual-labeled HBED-CC based PSMA inhibitors

Baranski, Ann-Christin, Schäfer, Martin, Bauder-Wüst, Ulrike, Haberkorn, Uwe, Eder, Matthias, Kopka, Klaus

OP25 [18F]MEL050 as a melanin PET tracer : fully automated radiosynthesis and evaluation for the detection of pigmented melanoma in mice pulmonary metastases

Chaussard M, Hosten B, Vignal N, Tsoupko-Sitnikov V, Hernio N, Hontonnou F, Merlet P, Poyet JL, Sarda-Mantel L, Rizzo-Padoin N

OP26 Design and Preclinical Evaluation of Novel Radiofluorinated PSMA Targeting Ligands Based on PSMA-617

J. Cardinale, M. Schäfer, M. Benešová, U. Bauder-Wüst, O. Seibert, F. Giesel, U. Haberkorn, M. Eder, K. Kopka

OP27 A novel radiolabeled peptide for PET imaging of prostate cancer: 64Cu-DOTHA2-PEG-RM26

Mansour Nematallah, Paquette Michel, Ait-Mohand Samia, Dumulon-Perreault Véronique, Lecomte Roger, Guérin Brigitte

OP29 Biodistribution of [^18^F]Amylovis®, a new radiotracer PET imaging of β-amyloid plaques

Fernandez-Maza L, Rivera-Marrero S, Prats Capote A, Parrado-Gallego A, Fernandez-Gomez I, Balcerzyk M, Sablon-Carrazana M, Perera-Pintado A, Merceron-Martinez D, Acosta-Medina E, Rodriguez-Tanty C

OP30 Synthesis and preclinical evaluation of [11C]-BA1 PET tracer for the imaging of CSF-1R

Bala Attili, Muneer Ahamed, Guy Bormans

OP31 In vivo imaging of the MCHR1 in the ventricular system via [18F]FE@SNAP

C. Philippe, M. Zeilinger, T. Scherer, C. Fürnsinn, M. Dumanic, W. Wadsak, M. Hacker, M. Mitterhauser

OP32 Synthesis of the first carbon-11 labelled P2Y12 receptor antagonist for imaging the anti-inflammatory phenotype of activated microglia

B. Janssen, D.J. Vugts, G.T. Molenaar, U. Funke, P.S. Kruijer, F. Dollé, G. Bormans, A.A. Lammertsma, A.D. Windhorst

OP33 Radiosynthesis of a selective HDAC6 inhibitor [11C]KB631 and in vitro and ex vivo evaluation

Koen Vermeulen, Muneer Ahamed, Michael Schnekenburger, Mathy Froeyen, Dag Erlend Olberg, Marc Diederich, Guy Bormansa

OP34 Improving metabolic stability of fluorine-18 labelled verapamil analogues

Raaphorst RM, Luurtsema G, Lammertsma AA, Elsinga PH, Windhorst AD

OP36 Development of a novel PET tracer for the activin receptor-like kinase 5

Lonneke Rotteveel, Uta Funke, Peter ten Dijke, Harm Jan Bogaard, Adriaan A. Lammertsma, Albert D. Windhorst

OP37 SPECT imaging and biodistribution studies of 111In-EGF-Au-PEG nanoparticles in vivo

Lei Song, Sarah Able, Nadia Falzone, Veerle Kersemans, Katherine Vallis

OP38 Melanoma targeting with [99mTc(N)(PNP3)]-labeled NAPamide derivatives: preliminary pharmacological studies

Davide Carta, Nicola Salvarese, Wiebke Sihver, Feng Gao, Hans Jürgen Pietzsch, Barbara Biondi, Paolo Ruzza, Fiorenzo Refosco, Cristina Bolzati

OP39 [^68^Ga]NODAGA-RGD: cGMP synthesis and data from a phase I clinical study

Roland Haubner, Armin Finkensted, Armin Stegmair, Christine Rangger, Clemens Decristoforo, Heinz Zoller, Irene J. Virgolin

OP44 Implementation of a GMP-grade radiopharmacy facility in Maastricht

Ivo Pooters, Maartje Lotz, Roel Wierts, Felix Mottaghy, Matthias Bauwens

OP45 Setting up a GMP production of a new radiopharmaceutical

Forsback, Sarita, Bergman Jörgen, Kivelä Riikka

OP48 In vitro and in vivo evaluation of 68-gallium labeled Fe3O4-DPD nanoparticles as potential PET/MRI imaging agents

M. Karageorgou, M. Radović, C. Tsoukalas, B. Antic, M. Gazouli, M. Paravatou-Petsotas, S. Xanthopouls, M. Calamiotou, D. Stamopoulos, S. Vranješ-Durić, P. Bouziotis

OP49 Fast PET imaging of inflammation using 68Ga-citrate with Fe-containing salts of hydroxy acids

A. S. Lunev, A. A. Larenkov, K.A. Petrosova, O. E. Klementyeva, G. E. Kodina

PP01 Installation and validation of 11C-methionine synthesis

Kvernenes, O.H., Adamsen, T.C.H.

PP02 Fully automated synthesis of 68Ga-labelled peptides using the IBA Synthera® and Synthera® Extension modules

René Martin, Sebastian Weidlich, Anna-Maria Zerges, Cristiana Gameiro, Neva Lazarova, Marco Müllera

PP03 GMP compliant production of ^15^O-labeled water using IBA 18 MeV proton cyclotron

Gert Luurtsema, Michèl de Vries, Michel Ghyoot, Gina van der Woude, Rolf Zijlma, Rudi Dierckx, Hendrikus H. Boersma, Philip H. Elsinga

PP04 In vitro Nuclear Imaging Potential of New Subphthalocyanine and Zinc Phthalocyanine

Fatma Yurt Lambrecht, Ozge Er, Mine Ince, Cıgır Biray Avci, Cumhur Gunduz, Fatma Aslihan Sarı

PP05 Synthesis, Photodynamic Therapy Efficacy and Nuclear Imaging Potential of Zinc Phthalocyanines

Kasim Ocakoglu, Ozge Er, Onur Alp Ersoz, Fatma Yurt Lambrecht, Mine Ince, Cagla Kayabasi, Cumhur Gunduz

PP06 Radio-U(H)PLC – the Search on the Optimal Flow Cell for the γ-Detector

Torsten Kniess, Sebastian Meister, Steffen Fischer, Jörg Steinbach

PP07 Radiolabeling, characterization & biodistribution study of cysteine and its derivatives with Tc99m

Rabia Ashfaq, Saeed Iqbal, Atiq-ur-Rehman, Irfan ullah Khan

PP08 Radiolabelling of poly (lactic-co.glycolic acid) (PLGA) nanoparticles with 99mTC

R Iglesias-Jerez, Cayero-Otero, L. Martín-Banderas, A. Perera-Pintado, I. Borrego-Dorado

PP09 Development of [^18^F]PD-410 as a non-peptidic PET radiotracer for gastrin releasing peptide receptors

Ines Farinha-Antunes, Chantal Kwizera, Enza Lacivita, Ermelinda Lucente, Mauro Niso, Paola De Giorgio, Roberto Perrone, Nicola A. Colabufo, Philip H. Elsinga, Marcello Leopoldo

PP10 An improved nucleophilic synthesis of 2-(3,4-dimethoxyphenyl)-6-(2-[18F]fluoroethoxy) benzothiazole ([18F]FEDMBT), potential diagnostic agent for breast cancer imaging by PET

V.V. Vaulina, O.S. Fedorova, V.V. Orlovskaja, С.L. Chen, G.Y. Li, F.C. Meng, R.S. Liu, H.E. Wang, R.N. Krasikova

PP11 Internal radiation dose assessment of radiopharmaceuticals prepared with accelerator-produced 99mTc

Laura Meléndez-Alafort, Mohamed Abozeid, Guillermina Ferro-Flores, Anna Negri, Michele Bello, Nikolay Uzunov, Martha Paiusco, Juan Esposito, Antonio Rosato

PP12 A specialized five-compartmental model software for pharmacokinetic parameters calculation

Laura Meléndez-Alafort, Cristina Bolzati, Guillermina Ferro-Flores, Nicola Salvarese, Debora Carpanese, Mohamed Abozeid, Antonio Rosato, Nikolay Uzunov

PP13 Molecular imaging of the pharmacokinetic behavior of low molecular weight ^18^F-labeled PEtOx in comparison to ^89^Zr-labeled PEtOx

Palmieri L, Verbrugghen T, Glassner M, Hoogenboom R, Staelens S, Wyffels L

PP14 Towards nucleophilic synthesis of the α-[18F]fluoropropyl-L-dihydroxyphenylalanine

V. V. Orlovskaja, O. F. Kuznetsova, O. S. Fedorova, V. I. Maleev, Yu. N. Belokon, A. Geolchanyan, A. S. Saghyan, L. Mu, R. Schibli, S. M. Ametamey, R. N. Krasikova

PP15 A convenient one-pot synthesis of [18F]clofarabine

Revunov, Evgeny, Malmquist, Jonas, Johnström, Peter, Van Valkenburgh, Juno, Steele, Dalton, Halldin, Christer, Schou, Magnus

PP16 BODIPY-estradiol conjugates as multi-modality tumor imaging agents

Samira Osati,Michel Paquette,Simon Beaudoin,Hasrat Ali,Brigitte Guerin, Jeffrey V. Leyton, Johan E. van Lier

PP17 Easy and high yielding synthesis of 68Ga-labelled HBED-PSMA and DOTA-PSMA by using a Modular-Lab Eazy automatic synthesizer

Di Iorio V, Iori M, Donati C, Lanzetta V, Capponi PC, Rubagotti S, Dreger T, Kunkel F, Asti M

PP18 Synthesis and evaluation of fusarinine C-based octadentate bifunctional chelators for zirconium-89 labelling

Chuangyan Zhai, Christine Rangger, Dominik Summer, Hubertus Haas, Clemens Decristoforo

PP19 Fully automated production of [18F]NaF using a re-configuring FDG synthesis module.

Suphansa Kijprayoon, Ananya Ruangma, Suthatip Ngokpol, Samart Tuamputsha

PP20 Extension of the Carbon-11 Small Labeling Agents Toolbox and Conjugate Addition

Ulrike Filp, Anna Pees, Carlotta Taddei, Aleksandra Pekošak, Antony D. Gee, Alex J. Poot, Albert D. Windhorst

PP21 In vitro studies on BBB penetration of pramipexole encapsulated theranostic liposomes for the therapy of Parkinson’s disease

Mine Silindir Gunay, A. Yekta Ozer, Suna Erdogan, Ipek Baysal, Denis Guilloteau, Sylvie Chalon

PP22 Factors affecting tumor uptake of 99mTc-HYNIC-VEGF165

Filippo Galli, Marco Artico, Samanta Taurone, Enrica Bianchi, Bruce D. Weintraub, Mariusz Skudlinski, Alberto Signore

PP23 Rhenium-188: a suitable radioisotope for targeted radiotherapy

Nicolas Lepareur, Nicolas Noiret, François Hindré, Franck Lacœuille, Eric Benoist, Etienne Garin

PP24 Preparation of a broad palette of 68Ga radiopharmaceuticals for clinical applications

Trejo-Ballado F, Zamora-Romo E, Manrique-Arias JC, Gama-Romero HM, Contreras-Castañon G, Tecuapetla-Chantes RG, Avila-Rodriguez MA

PP25 68Ga-peptide preparation with the use of two 68Ge/68Ga-generators

H. Kvaternik, D. Hausberger, C. Zink, B. Rumpf, R. M. Aigner

PP26 Assay of HEPES in 68Ga-peptides by HPLC

H. Kvaternik, D. Hausberger, B. Rumpf, R. M. Aigner

PP27 Preparation, in vitro and in vivo evaluation of a 99mTc(I)-Diethyl Ester (S,S)-Ethylenediamine- N,N´-DI-2-(3-Cyclohexyl) Propionic acid as a target-specific radiopharmaceutical

Drina Janković, Mladen Lakić, Aleksandar Savić, Slavica Ristić, Nadežda Nikolić, Aleksandar Vukadinović, Tibor J. Sabo, Sanja Vranješ-Đurić

PP28 90Y-labeled magnetite nanoparticles for possible application in cancer therapy

S. Vranješ-Đurić, M. Radović, D. Janković, N. Nikolić, G. F. Goya, P. Calatayud, V. Spasojević, B. Antić

PP29 Simplified automation of the GMP production of 68Ga-labelled peptides

David Goblet, Cristiana Gameiro, Neva Lazarova

PP30 Combining commercial production of multi-products in a GMP environment with Clinical & R&D activities

Cristiana Gameiro, Ian Oxley, Antero Abrunhosa, Vasko Kramer, Maria Vosjan, Arnold Spaans

PP31 ^99^mTc(CO)3-labeling and Comparative In-Vivo Evaluation of Two Clicked cRGDfK Peptide Derivatives

Kusum Vats, Drishty Satpati, Haladhar D Sarma, Sharmila Banerjee

PP32 Application of AnaLig resin for 99mTc separation from molybdenum excess

Wojdowska W., Pawlak D.W., Parus L. J., Garnuszek P., Mikołajczak R.

PP33 Constraints for selection of suitable precursor for one-step automated synthesis of [18F]FECNT, the dopamine transporter ligand

Pijarowska-Kruszyna J, Jaron A, Kachniarz A, Malkowski B, Garnuszek P, Mikolajczak R

PP34 Gamma scintigraphy studies with 99mTc- amoxicillin sodium in bacterially infected and sterile inflamed rats

Derya Ilem-Ozdemir, Oya Caglayan-Orumlu, Makbule Asikoglu

PP35 Preparation of 99mTc- Amoxicillin Sodium Lyophilized Kit

Derya Ilem-Ozdemir, Oya Caglayan-Orumlu, Makbule Asikoglu

PP36 Outfits of Tracerlan FXC-PRO for 11C-Labeling

Arponen Eveliina, Helin Semi, Saarinen Timo, Vauhkala Simo, Kokkomäki Esa, Lehikoinen Pertti

PP37 Microfluidic synthesis of ω-[18F]fluoro-1-alkynes

Mariarosaria De Simone, Giancarlo Pascali, Ludovica Carzoli, Mauro Quaglierini, Mauro Telleschi, Piero A. Salvadori

PP38 Automated 18F-flumazenil production using chemically resistant disposable cassettes

Phoebe Lam, Martina Aistleitner, Reinhard Eichinger, Christoph Artner

PP39 The effect of the eluent solutions (TBAHCO3, Kryptand K2.2.2) on the radiochemical yields of 18F-Fluoromethylcholine

Surendra Nakka, Hemantha Kumara MC, Al-Qahtani Mohammed

PP40 [68Ga]Radiolabeling of short peptide that has a PET imaging potentials

Al-Qahtani, Mohammed, Al-Malki, Yousif

PP41 Is validation of radiochemical purity analysis in a public hospital in a developing country possible?

N Mambilima, SM Rubow

PP42 Improved automated radiosynthesis of [18F]FEPPA

N. Berroterán-Infante, M. Hacker, M. Mitterhauser, W. Wadsak

PP43 Synthesis and initial evaluation of Al18F-RESCA1-TATE for somatostatin receptor imaging with PET

Uta Funke, Frederik Cleeren, Joan Lecina, Rodrigo Gallardo, Alfons M. Verbruggen, Guy Bormans

PP44 Radiolabeling and SPECT/CT imaging of different polymer-decorated zein nanoparticles for oral administration

Rocío Ramos-Membrive, Ana Brotons, Gemma Quincoces, Laura Inchaurraga, Inés Luis de Redín, Verónica Morán, Berta García-García, Juan Manuel Irache, Iván Peñuelas

PP45 An analysis of the quality of 68Ga-DOTANOC radiolabelling over a 3 year period

Trabelsi, M., Cooper M.S.

PP46 In vivo biodistribution of adult human mesenchymal stem cells I (MSCS-ah) labeled with 99MTC-HMPAO administered via intravenous and intra-articular in animal model. Preliminary results

Alejandra Abella, Teodomiro Fuente, Antonio Jesús Montellano, Teresa Martínez, Ruben Rabadan, Luis Meseguer-Olmo

PP47 Synthesis of [^18^F]F-exendin-4 with high specific activity

Lehtiniemi P, Yim C, Mikkola K, Nuutila P, Solin O

PP48 Experimental radionuclide therapy with 177Lu-labelled cyclic minigastrin and human dosimetry estimations

von Guggenberg E, Rangger C, Mair C, Balogh L, Pöstényi Z, Pawlak D, Mikołajczak R

PP49 Synthesis of radiopharmaceuticals for cell radiolabelling using anion exchange column

Socan A, Kolenc Peitl P, Krošelj M, Rangger C, Decristoforo C

PP50 [68Ga]peptide production on commercial synthesiser mAIO

Collet C., Remy S., Didier R,Vergote T.,Karcher G., Véran N.

PP51 Dry kit formulation for efficient radiolabeling of 68Ga-PSMA

D. Pawlak, M. Maurin, P. Garnuszek, U. Karczmarczyk, R. Mikołajczak

PP52 Development of an experimental method using Cs-131 to evaluate radiobiological effects of internalized Auger-electron emitters

Pil Fredericia, Gregory Severin, Torsten Groesser, Ulli Köster, Mikael Jensen

PP53 Preclinical comparative evaluation of NOTA/NODAGA/DOTA CYCLO-RGD peptides labelled with Ga-68

R. Leonte, F. D. Puicea, A. Raicu, E. A. Min, R. Serban, G. Manda, D. Niculae

PP54 Synthesizer- and Kit-based preparation of prostate cancer imaging agent 68Ga-RM2

Marion Zerna, Hanno Schieferstein, Andre Müller, Mathias Berndt

PP55 Synthesis of pancreatic beta cell-specific [18F]fluoro-exendin-4 via strain-promoted aza-dibenzocyclooctyne/azide cycloaddition

Cheng-Bin Yim, Kirsi Mikkola, Pirjo Nuutila, Olof Solin

PP56 Automated systems for radiopharmacy

D. Seifert, J. Ráliš, O. Lebeda

PP57 Simple, suitable for everyday routine use quality control method to assess radionuclidic purity of cyclotron-produced 99mTc

Svetlana V. Selivanova, Helena Senta, Éric Lavallée, Lyne Caouette, Éric Turcotte, Roger Lecomte

PP58 Effective dose estimation using Monte Carlo simulation for patients undergoing radioiodine therapy

Marina Zdraveska Kochovska, Emilija Janjevik Ivanovska, Vesna Spasic Jokic

PP59 Chemical analysis of the rituximab radioimmunoconjugates in lyophilized formulations intended for oncological applications

Darinka Gjorgieva Ackova, Katarina Smilkov, Petre Makreski, Trajče Stafilov, Emilija Janevik-Ivanovska

PP61 The need and benefits of established radiopharmacy in developing African countries

Aschalew Alemu, Joel Munene Muchira, David Mwanza Wanjeh, Emilija Janevik-Ivanovska

PP62 University Master Program of Radiopharmacy – step forward for Good Radiopharmacy Education

Emilija Janevik-Ivanovska, Zoran Zdravev, Uday Bhonsle, Osso Júnior João Alberto, Adriano Duatti, Bistra Angelovska, Zdenka Stojanovska, Zorica Arsova Sarafinovska, Darko Bosnakovski, Darinka Gorgieva-Ackova, Katarina Smilkov, Elena Drakalska, Meera Venkatesh, Rubin Gulaboski

PP63 Synthesis and preclinical validations of a novel 18F-labelled RGD peptide prepared by ligation of a 2-cyanobenzothiazole with 1,2-aminothiol to image angiogenesis.

Didier J. Colin, James A. H. Inkster, Stéphane Germain, Yann Seimbille

## ORAL PRESENTATIONS

### OP03 Selective extraction of medically-related radionuclides from proton-irradiated thorium targets

#### V. Radchenko^1^, J. W. Engle^1^, C. Roy^2^, J. Griswold^2^, M. F. Nortier^1^, E. R. Birnbaum^1^, M. Brugh^1^, S. Mirzadeh^2^, K. D. John^1^, M. E. Fassbender^1^

##### ^1^Los Alamos National Laboratory, Los Alamos, New Mexico, USA; ^2^Oak Ridge National Laboratory, Oak Ridge, Tennessee, USA

###### **Correspondence:** V. Radchenko – Los Alamos National Laboratory, Los Alamos, New Mexico, USA

**Background:** Clinicians rely on nuclear medicine for the treatment of numerous diseasesimpacting millions of patients annually. Recently, Targeted Radiotherapy (TR) has been successfully advanced with the US FDA approval of several radionuclide based drugs]. Combinations of several types of radionuclide emissions for therapy (i.e., α-therapeutic agent combined with β^-^ therapeutic) could lead to even more effective treatment options. One of the limiting factors in the development of TR as a widely adopted treatment option is the availability of select radionuclides with optimum emission properties (both in volume and periodicity of delivery), which poses a challenge due to the fact that different radionuclides typically require different target materials and/or nuclear reaction pathways for their formation.

**Materials and** **methods:** We already published a successful strategy for the isolation of ^225/227^Ac from irradiated thorium targets [5]. We also published the recovery of Pa isotopes [6] from proton irradiated thorium. In this work, we propose the isolation of several other medically related radionuclides namely ^103^Ru, ^223/225^Ra, ^111^Ag from the same target material.

**Results:** Several methods based on ion exchange chromatography and solid phase extraction show promise for the co-extraction of ^103^Ru and Ra isotopes from thorium irradiated targets. Anion exchange in HCl media proved to be an efficient method for the isolation of ^103^Ru, while a combination of cation exchange resin/citrate and DGA resin/HNO_3_ is suitable for Ra isotopes separation.

**Discussion/conclusion:** Production yields for the proposed radionuclides were evaluated by comparison of actual product yields with calculated (predicted) yields. Radiochemical strategies for co-extraction of ^103^Ru and ^223/225^Ra isotopes based on ion exchange and solid phase extraction chromatography will be discussed.

### OP04 Comparison of [68Ga]FSC(succ-RGD)3 and [68Ga]NODAGA-RGD for PET imaging of αvβ3 integrin expression

#### Chuangyan Zhai^1^, Gerben M. Franssen^2^, Milos Petrik^3^, Peter Laverman^2^, Clemens Decristoforo^1^

##### ^1^Department of Nuclear Medicine, Medical University Innsbruck, Innsbruck, Austria; ^2^Department of Radiology & Nuclear Medicine, Radboud University Medical Center, Nijmegen, The Netherlands; ^3^Institute of Molecular and Translational Medicine, Faculty of Medicine and Dentistry, Palacky University, Olomouc, Czech Republic

###### **Correspondence:** Chuangyan Zhai **–** Department of Nuclear Medicine, Medical University Innsbruck, Innsbruck, Austria

**Background:** The arginine-glycine-aspartic (RGD) peptide sequence serves as a high-affinity antagonist of the integrin α_v_β_3_ receptor that plays an important role in tumor angiogenesis. Recently we reported [^68^Ga]FSC(succ-RGD)_3_, a trimeric RGD peptide, exhibited excellent targeting properties for α_v_β_3_ integrin expression and significant improved tumor uptake compared to monomeric [^68^Ga]NODAGA-RGD.(1) Here we report the PET imaging properties of [^68^Ga]FSC(succ-RGD)_3_ in different xenograft tumor model and compared them with [^68^Ga]NODAGA-RGD.

**Materials and methods:** The PET imaging properties of [^68^Ga]FSC(succ-RGD)_3_ were studied in nude mice bearing M21 human melanoma xenografts and human glioblastoma U87MG xenograft tumor. A parallel PET imaging of ^68^GaNODAGA-RGD in same mouse bearing U87MG xenograft tumor was performed as a comparison.

**Results:** The static PET image of [^68^Ga]FSC(succ-RGD)_3_ in nude mice showed highly visualized tumors of M21 (positive) whereas nonvisualized tumor of M21-L (negative) tumor xenografts 1 h post injection confirming receptor-specific activity accumulation. The dynamic PET images of [^68^Ga]FSC(succ-RGD)_3_ showed rapid clearance of [^68^Ga]FSC(succ-RGD)_3_ from the circulation while the tumor remained clearly visible. A direct comparison of [^68^Ga]FSC(succ-RGD)_3_ with [^68^Ga]NODAGA-RGD in nude mice bearing U87MG xenograft tumor using PET/CT resulted comparable target/background ratio (tumor/kidneys ratio = 1.3 and 1.6, tumor/muscle ratio = 4.9, 5, respectively, 90 min post injection). The time activity curves from dynamic PET data showed an increase of the activity concentration of [^68^Ga]FSC(succ-RGD)_3_ in tumor firstly, then remained almost constant whereas that of [^68^Ga]NODAGA-RGD decreased quickly. The significant enhanced tumor uptake (3.8 vs. 1.6 % ID/g) in addition to the slower washout rate from tumor for [^68^Ga]FSC(succ-RGD)_3_ not only allows the PET imaging at late time points, but also provides the possibility to achieve the same image contrast using less radioactivity as well as detect low-level integrin expression.

**Discussion/conclusion:** [^68^Ga]FSC(succ-RGD)_3_ shows the advantages in the respect of delayed imaging, reduced radiation dose as well as monitoring low-level integrin expression in tissues in comparison to [^68^Ga]NODAGA-RGD, therefore it is a promising agent for integrin α_v_β_3_ receptor imaging.

### OP05 A new NPY-Y1R targeting peptide for breast cancer PET imaging

#### Ait-Mohand Samia^1^, Dumulon-Perreault Véronique^2^, Guérin Brigitte^1,2^

##### ^1^Département de Médecine Nucléaire et Radiobiologie, Faculté de Médecine et Sciences de la Santé, Université de Sherbrooke, QC, Canada J1H5N4; ^2^ Centre d’Imagerie Moléculaire de Sherbrooke (CIMS), CR-CHUS, Sherbrooke, QC, Canada J1H5N4

###### **Correspondence:** Guérin Brigitte – Département de Médecine Nucléaire et Radiobiologie, Faculté de Médecine et Sciences de la Santé, Université de Sherbrooke, QC, Canada J1H5N4

**Background:** NPY-Y1 receptor (NPY-Y1R) is a promising target for breast cancer imaging. Previously, our group prepared and tested a series of truncated NPY analogs derived from BVD-15 (; [Pro^30^, Tyr^32^, Leu^34^]NPY(28-36)-NH_2_) for ^64^Cu-labeling). Unfortunately the biological half-live of the most potent tracer, [Lys(^64^Cu/DOTA)^4^]BVD15, when injected in mouse plasma was shorter than 15 minutes. In this study, we improved the design of BVD15 in order to increase its stability *in vitro* and *in vivo* and maintain its targeting capability.

**Materials and methods:** Modifications of the peptide backbone, the chelator and the use of D- and non-naturel amino acids were proposed to improve the peptide tracer stability. The peptides were synthesized on solid phase and conjugated to NOTA chelator. Binding studies on MCF-7 human breast cancer cells (2) were performed after each structural modification to make sure that the potency and the selectivity of the new NOTA-peptide conjugates to NPY-Y1R were maintained. Once active compounds were identified, they were radiolabeled with ^64^Cu for performing plasma stability, cellular uptake, internalization, and blocking studies on MCF-7 cells in order to rapidly identify the promising candidates for *in vivo* studies.

**Results:** A ^64^Cu/NOTA-BVD15 derivative presenting a very low K_i_ (9 nM) and showing a very high stability in plasma up to 20 h and *in vivo* for 30 minutes has been identified. Cell assays showed a constant uptake and internalization over the whole experiment. The internalized fraction after 2h was ~20%. The radiopeptide uptake was blocked in presence of an excess of unlabeled peptide.

**Discussion/conclusion:** We have identified a new ^64^Cu-labeled peptide presenting a good stability and an excellent affinity to NPY-Y1R. On the basis of the cellular results, the ^64^Cu/NOTA-BVD15 derivative appears to have a potential for the targeting of NPY-Y1R positive tumors.

### OP06 The influence of multivalency on CCK 2 receptor targeting

#### D. Summer^1^, A. Kroess^1^, C. Rangger^1^, H. Haas^2^, P. Laverman^3^, F. Gerben^3^, E. von Guggenberg^1^, C.Decristoforo^1^

##### ^1^Department of Nuclear Medicine, Medical University Innsbruck, Innsbruck, Austria; ^2^Division of Molecular Biology/Biocenter, Medical University Innsbruck, Innsbruck, Austria; ^3^Department of Nuclear Medicine, Radboud University Medical Center, Nijmegen, The Netherlands

###### **Correspondence**: D. Summer – Department of Nuclear Medicine, Medical University Innsbruck, Innsbruck, Austria

**Background**: Multivalency has shown to enhance accumulation of receptor targeted radiopharmaceuticals, due to the avidity effect and increased apparent tracer concentration. For metabolically unstable peptides multimerisation may also delay metabolic processes like oxidation and enzymatic cleavage leading to loss of binding affinity. The aim of this study was to show the benefit of multimeristation on the targeting behaviour using a CCK2-receptor targeting, metabolically unstable Minigastrin peptide (MG11).

**Materials and methods:** Fusarinine C, a cyclic siderophore bearing three amino residues, was chosen as chelating agent and up to three peptide sequences (MG11= D-Glu1, desGlu2-6]-minigastrin) were conjugated by using maleimide chemistry. Radiotracers were labelled with 68Ga as well as with 89Zr following standard radiolabeling protocols. For in vitro characterisation stability studies, determination of the partition coefficient and cell uptake studies were performed. In vivo experiments included biodistribution studies (1h p.i) and static micro PET/CT imaging and were carried out in tumour xenograft bearing balb/c nude mice.

**Results**: Peptide conjugates could be labelled with 68Ga (5-15min RT, pH4-5) and 89Zr (60min, RT, pH7) achieving radiochemical yields of more than 98%. All complexes showed excellent stabilities in the presence of EDTA, DTPA, FeCl3 and in human serum after certain timepoints. Partition coefficient (logP) values ranging from -1 to -3 revealed a hydrophilic character of the radiotracers. As expected a decrease of hydrophilicity correlated with increasing grade of multimerisation. The cell uptake ranged from 10 to 15% per mg protein and could be reduced significantly by blocking with human minigastrin indicating specific receptor binding of all conjugates. Biodistribution studies showed a tumour uptake ranging from 5% (monomer) to approximately 10% ID/g (multimer) one hour post injection. Tumour to organ ratio was decreased by multimerisation and especially the kidney retention was increased significantly by the di- and trimeric radiotracers. Static animal imaging one and two hours after injection of 68Ga labeled conjugates confirmed the outcome of the biodistribution studies. 89Zr labeled counterparts showed very similar results but after 24 hours post injection the trimeric bioconjugate showed much better tumour imaging ability than the corresponding mono- and dimer.

**Discussion/conclusion**: Though these results are preliminary multimerisation seems to correlate with higher tumour uptake leading to better late timepoint imaging but high kidney retention seems to be a major limitation. Stability studies are ongoing.

### OP07 SPECT Imaging of αvβ3 Expression by [99mTc(N)PNP43]- Bifunctional Chimeric RGD Peptide not Cross-Reacting with αvβ5

#### Cristina Bolzati^1^, Nicola Salvarese^1,2^, Fiorenzo Refosco^1^, Laura Meléndez-Alafort^2^, Debora Carpanese^2^, Antonio Rosato^2,3^, Michele Saviano^4^, Annarita Del Gatto^5^, Daniela Comegna^5^, Laura Zaccaro^5^

##### ^1^IENI-CNR, Padua, Italy; ^2^DiSCOG-University of Padua, Padua, Italy; ^3^IOV Padua, Padua, Italy; ^4^IC-CNR, Bari, Italy; ^5^IBB-CNR, Naples, Italy

###### **Correspondence:** Cristina Bolzati – IENI-CNR, Padua, Italy

**Background:** Recently a new bifunctional chimeric RGD peptide (RGDechi), comprising a cyclic Arg-Gly-Asp pentapeptide covalently bound to an echistatin domain, has been reported^1^. *In vitro* and *in vivo* biological studies evidenced that this chimeric peptide selectively binds to αvβ3 integrin and does not cross-react with αvβ5^2^. In order to obtain an optimal SPECT radiotracer, a series of [^99m^Tc(N)PNP] labelled peptides has been prepared and their pharmacological properties investigate.

**Materials and methods:** RGDechi-hCit (**1**) and three truncated peptide derivatives [RGDechi1_17 (**2**), RGDechi1_16 (**3**) and RGDechi1_14 (**4**)] lacking the two, three and five C-terminal amino acids, were synthetized in solid phase by Fmoc chemistry and conjugated with a cysteine linked to the Lys1 side chain to allow the labeling with [^99m^Tc(N)PNP43]-synthon (PNP43 = (CH_3_)_2_P(CH_2_)_2_N(C_2_H_4_OCH_3_)(CH_2_)_2_P(CH_3_)_2_). *In vitro* stability and pharmacological parameters of the corresponding compounds, ^**99m**^ **Tc1-4,** were assessed. Challenges with an excess of glutathione and cysteine and Log P values were also investigated. Furthermore, radiolabeled peptides (^**99m**^ **Tc1-4**) were applied to study *in vivo* stability and the pharmacokinetic profiles on tumor bearing mice.

**Results:** All ^99m^Tc-compounds were obtained with RCY > 90%. Log P values demonstrate the hydrophilic nature of the radiolabeled peptides ranging from -2.96 to -2.12. No significant variations in RCPs of the complexes were detected in challenge experiments with an excess (10 mM) of glutathione and cysteine. In general, a high *in vitro* stability was observed after incubation in human and mice sera as well as in mice liver homogenate; a slight degradation of ^**99m**^ **Tc1-4** was found in kidneys homogenate. Cell uptake assays showed that, excluding ^**99m**^ **Tc4** compound, ^**99m**^ **Tc1-3** radiolabeled peptides accumulate selectively in cells expressing αvβ3 integrin and does not accumulate in cell expressing moderate levels of αvβ5 and undetectable levels of αvβ3 integrins. In agreement with *in vitro* findings, biodistribution studies showed that the ^**99m**^ **Tc1-3** radiolabeled chimeric peptide selectively localizes in tumor xenografts expressing αvβ3 and fails to accumulate in those expressing αvβ5 integrin.

**Discussion/conclusion:** ^99m^Tc-labeled RGDechi, RGDechi1_17 and RGDechi1_16 chimeric peptides can be used for highly selective α_v_β_3_ expression imaging by SPECT technology. Among the tested compounds, ^**99m**^ **Tc2** possess the best distribution profile and highest localization in tumor expressing αvβ3. This research was supported by MIUR through PRIN 20097FJHPZ-004 and FIRB “RINAME”2010-RBAP114AMK, by Programma Operativo Nazionale Ricerca e Competitivita PON 01_02388 and by Italian Association for Cancer Research AIRC IG 13121.

### OP09 New dienophiles for the inverse-electron-demand Diels-Alder reaction and for pretargeted PET imaging

#### Emilie Billaud^1^, Muneer Ahamed^1^, Frederik Cleeren^1^, Elnaz Shahbazali^2^, Tim Noël^2^, Volker Hessel^2^, Alfons Verbruggen^1^, Guy Bormans^1^

##### ^1^Laboratory of Radiopharmacy, KU Leuven, Leuven, Belgium; ^2^Micro Flow Chemistry & Process Technology, Chemical Engineering and Chemistry Department, TU Eindhoven, Eindhoven, The Netherlands

###### **Correspondence:** Emilie Billaud – Laboratory of Radiopharmacy, KU Leuven, Leuven, Belgium

**Introduction:** In cancer research, pretargeted PET imaging has emerged as an effective two-step approach that combines the affinity and selectivity of antibodies with the rapid pharmacokinetics and favorable dosimetry of smaller molecule radiolabeled with short-lived radionuclides. This approach can be based on the bioorthogonal inverse-electron-demand Diels-Alder (IEDDA) “click” reaction between tetrazines and *trans*-cyclooctene (TCO) derivatives. Our project aims to develop new [^18^F]TCO-dienophiles with high reactivity for the IEDDA reaction, improved *in vivo* stability and favorable pharmacokinetics. New dienophiles were synthesized using an innovative continuous-flow micro-photochemistry process, and their reaction kinetics with a tetrazine were determined. *In vivo* stability studies of the most promising ^18^F-radiolabeled-TCO-derivative ([^18^F]*trans*-EB-70) was investigated, and its potential for pretargeted PET imaging was assessed.

**Materials and Methods:** ***Organic chemistry*** Fluoro-*cis*-cyclooctene derivatives and mesylate precursors for radiofluorination were synthesized in 5-8 steps. Structures of intermediates and final compounds were confirmed by NMR and HRMS. ***Photochemistry*** *Trans*-for-*cis* isomerization was performed using a microfluidic setup. ***Kinetics*** Reactions between dienophiles and 3,6-di(pyridin-2-yl)-1,2,4,5-tetrazine in MeOH at 25°C were monitored by UV-spectrophotometry at 290 nm (pseudo-first order conditions). ^***18***^ ***F-radiolabeling (semi-automated)*** Nucleophilic substitution of mesylate *trans*-EB-77 using [^18^F]KF,K_222_ was achieved in MeCN at 90°C for 15 min. [^18^F]*trans*-EB-70 was purified by HPLC. ***In vivo stability*** [^18^F]*trans*-EB-70 was evaluated in healthy NMRI mice, by *ex vivo* biodistribution (2, 10, 30, 60 min p.i.). ***In vitro pretargeting*** Prostate tumor slices (LNCaP and PC-3 cells) were successively incubated with a prostate-specific membrane antigen (PSMA) inhibitor modified with 3-(4-(trifluoromethyl)phenyl)-6-phenyl-1,2,4,5-tetrazine, and [^18^F]*trans*-EB-70. Direct incubation with the corresponding [^18^F]”preclicked”-compound, and blocking experiments (2-(phosphonomethyl)pentane-1,5-dioic acid) were performed.

**Results** Fluoro-*cis*-cyclooctene derivatives and mesylate precursors were synthesized in 3-35% overall yields. The *trans*-for-*cis* micro-photoisomerization reached yields of 48%. Reaction kinetics of the new dienophiles are fast, with k_2_ ranging from 475.6±32.8 to 1913.0±195.9 M^-1^.s^-1^. Radiosynthesis of [^18^F]*trans*-EB-70 was achieved in 60 min, with 12% radiochemical yield (decay-corrected), a radiochemical purity >99% (for at least 2h), and 70-188 GBq.μmol^-1^ specific activity. Biodistribution of [^18^F]*trans*-EB-70 in mice demonstrated absence of *in vivo* defluorination and a fast clearance *via* urinary and hepatobiliary systems. Regarding *in vitro* experiment, the binding on LNCaP tumor slices (expressing PSMA receptors) subjected to pretargeting was PSMA-specific and slightly inferior to the binding of [^18^F]”preclicked”-compound. No significant binding was observed in PC-3 cells (negative control).

**Discussion/conclusion:** We demonstrated that [^18^F]*trans*-EB-70 is a suitable dienophile for the IEDDA “click” reaction and for pretargeting applications. Therefore, [^18^F]*trans*-EB-70 will be investigated further in pretargeted μPET experiments.

**Research support:** SBO MIRIAD (IWT Flanders)

### OP10 New complexing agent for Al18F-labelling of heat-sensitive biomolecules: Synthesis and preclinical evaluation of Al18F-RESCA1-HAS

#### Cleeren F^1^, Lecina J^1^, Koole M^2^, Verbruggen A^1^, Bormans G^1^

##### ^1^Laboratory for Radiopharmacy, University of Leuven, Leuven, Belgium; ^2^Department of Nuclear Medicine and Molecular Imaging, University of Leuven, Leuven, Belgium

###### **Correspondence:** Cleeren F – Laboratory for Radiopharmacy, University of Leuven, Belgium

**Introduction:** The Al18F-labelling strategy involves formation of aluminium mono[18F]fluoride ({Al18F}2+) which is trapped by a suitable chelator –mostly bound to a biomolecule- in aqueous medium.1At this moment however, the need for elevated temperatures (100-120 °C) limits its widespread use. Therefore, we designed new restrained complexing agents (RESCAs) for use of this strategy at moderate temperature. RESCA1 is an acyclic pentadentate ligand with a N2O3 coordinative set that is able to complex {Al18F}2+ efficiently at 25 °C. To evaluate the stability and kinetic inertness of the chelate in vivo, RESCA1 was conjugated to human serum albumin (HSA) and labelled with {Al18F}. The Al18F-labelled conjugate was monitored in vivo for 6 h p.i.

**Materials and methods:** HSA was reacted with RESCA1-TFP. RESCA1-HSA (7.5 mg, 110 nmol) in 750 μl sodium acetate buffer (0.1 M, pH 4.5) was added to a freshly prepared {Al^18^F}^2+^ solution (1.4 GBq, 50 nmol AlCl_3_, 12 min, RT). The product was purified using a PD-10 column and RCP was determined with SEC-HPLC. To test stability in serum, Al^18^F-RESCA1-HSA in 100 μl PBS was added to rat serum (900 μl), kept at 37°C and monitored up to 4 h. *Ex vivo* biodistribution was studied at 1 h, 3 h and 6 h p.i. of Al^18^F-RESCA1-HSA (2-7.5 MBq) in healthy female rats. Small-animal whole-body PET imaging was performed using a FOCUS 220 tomograph.

**Results:** RESCA1-HSA was obtained with a chelator-to-protein ratio of 3, estimated by ESI-TOF-HRMS analysis. Al18F-RESCA-HSA was prepared in high RCY (>70%) and purity (>95%) in < 30 min. 91% of product was still intact in rat serum after 4 h incubation. Distribution in rats showed high retention in blood with 5.42 ± 0.23% ID/g, 4.93 ± 0.22% ID/g and 3.66 ± 0.06% ID/g at 1h, 3h and 6 h respectively from which the blood biological half-life was calculated to be 8.6 h. No significant increase in bone uptake was observed, indicating excellent in vivo stability of the Al18F-labelled construct.

**Discussion/conclusion:** We successfully labelled for the first time a heat-sensitive biomolecule via the Al18F-method in one radiolabelling step. Al18F- RESCA1-HSA showed excellent stability and favourable properties for PET blood pool imaging applications.

### OP11 A novel versatile precursor efficient for F-18 radiolabelling via click-chemistry

#### B. Lugatoa^1^, S. Stucchia^1^, E.A. Turollaa^1^, L. Giulianoa^1^, S.Toddea^1^, P. Ferraboschib^2^

##### ^1^Department of Medicine and Surgery, Tecnomed Foundation, University of Milano-Bicocca, Milan, Italy; ^2^Dipartimento di Biotecnologie Mediche e Medicina Traslazionale, Università degli Studi di Milano, Milan, Italy

###### **Correspondence:** B. Lugatoa – Department of Medicine and Surgery, Tecnomed Foundation, University of Milano-Bicocca, Milan, Italy

**Introduction** In the last years, the Cu(I)-catalyzed Huisgen [3+2] cycloaddition between terminal alkynes and azides emerged as a powerful tool in F-18 radiolabelling of biomolecules such as peptides, because of its regioselectivity, mild aqueous organic conditions, reduced reaction times, and high yields.^(1)^.A weak point of the method is the lack of suitable commercially available, stable precursors.^(2)^ In this paper we report the synthesis and F-18 radiolabelling of a new, versatile, easy to handle, and stable azido precursor useful for *click-chemistry*.

**Materials and methods:** Reagents and solvents were purchased from Sigma-Aldrich. [^18^F]Fluoride was produced with an IBA Cyclone 18/9 cyclotron. Radioactive synthesis were carried out on a fully automated radiosynthesis module (GE TracerLab FX-FN Pro), and analyzed by analytical RP-HPLC with UV and radiochemical detectors. Non-radioactive compounds were fully characterized by NMR, ESI-MS and IR.

**Results:** A series of bifunctional precursors, bearing the azido moiety and different leaving groups (e.g. tosylate, mesylate, iodo), coupled to a short polyetyleneglycol chain (to improve their stability and hydrophilicity) were designed and successfully prepared following a ten-step synthetic pathway. A protection-deprotection strategy of functional groups achieved the precursors and the fluorinated reference with good yield and purity. Precursors were radiolabelled with F-18 and then coupled to propargylglycine as alkyne counterpart. [^18^F]Fluoride was purified following standard procedure, and nucleophilic displacement of the iodo leaving group took place at 100° C, in 20 min. The resulting labelled azide was successfully purified using a Sep-Pak tC18 cartridge (51% radiochemical yield not decay corrected, 93% radiochemical purity). The purified azide was then conjugated to propargylglycine, showing 52% conversion within 30 min at room temperature. Purification and formulation have still to be optimized.

**Discussion/conclusion:** A new optimized precursor useful for F-18 radiolabelling and *click-chemistry* was prepared. It demonstrated to be effective in radiolabelling non-protected alkynyl-modified aminoacids, by a fully automated synthetic procedure. Good results, in terms of radiochemical yield and purity, were obtained from the iodo-derivative precursor. Purification and formulation of the final cycloaddition product are in progress.

### OP12 A general applicable method to quantify unidentified UV impurities in radiopharmaceuticals

#### R.P. Klok^1^, M.P.J. Mooijer^1^, N.H. Hendrikse^1,2^, A.D. Windhorst^1^

##### ^1^Department of Radiology & Nuclear Medicine, VU University Medical Center, Amsterdam, The Netherlands; ^2^Department of Clinical Pharmacology & Pharmacy, VU University Medical Center, Amsterdam, The Netherlands

**Introduction:** Radiopharmaceuticals are released for administration by a quality control procedure against pre-set specifications. One of these release specifications is the chemical purity of the drug product, determined with High Pressure Liquid Chromatography (HPLC) and UV detection. In the European Pharmacopeia (EP) hardly any specifications are given for the chemical purity of a radiopharmaceutical. When unknown impurities are present in the chromatogram, the decision if the radiopharmaceutical can be released, is very frequently based on unclear parameters like ‘no unidentified UV signals present’. There is a need for an objective specification in order to have a save and reliable release.

**Aim:** The purpose of the presented work is to define a generally applicable method to define tolerances for unidentified impurities in radiopharmaceuticals.

**Materials and methods:** A retrospective analysis was performed on HPLC analysis results of [11C]Flumazenil, [11C]PIB, [11C]Erlotinib, [11C]DPA713, [18F]PK209 and [18F]FES. Quantification of the carrier signal in the UV chromatogram was determined by use of calibration curves, utilizing Chromeleon® 6.8. Unidentified impurities were semi-quantified utilizing the surface area in the UV chromatogram relative to the quantified carrier signal. Based on the EP. monography of [11C]Flumazenil and [18F]FET, the specification for unidentified UV impurities was determined to be 0.22 pmol/injection volume for a single unidentified impurity and 0.88 pmol/injection volume for the total of unidentified impurities. This specification was tested for over 500 batches of the radiopharmaceuticals and compared to the less specific parameter ‘no unidentified UV signals present’.

**Results:** In a pilot assessment we encountered in 5-10% cases unidentified UV impurities leading to rejection of the batch, based on the specification ‘no unidentified UV signals present’. Of these rejected batches 25% was also rejected with the new defined specification. Reason for this reduced number of rejections is that with the new specification the presence of unidentified impurities is evaluated objectively. The analysis of the full database is currently on-going.

**Discussion/conclusion:** With this method the amount of unidentified impurities can be estimated in an optimal and objective way, utilizing EP limits of [11C]Flumazenil and [18F]FET, without operator variability.

### OP13 Development of [^18^F]Fluoro-C-glycosides to radiolabel peptides

#### Collet C.^1,2^, Petry N.^1,3^, Chrétien F.^1,3^, Karcher G.^1,2,4^, Pellegrini-Moïse N.^1,3^, Lamandé-Langle S.^1,3^

##### ^1^Université de Lorraine, F-54500 Vandoeuvre les Nancy – France; ^2^NancycloTEP, Plateforme d’imagerie expérimentale, 54500 Vandoeuvre les Nancy – France; ^3^CNRS, UMR 7565 SRSMC, F-54506 Vandoeuvre les Nancy – France; ^4^CHU de Nancy-Brabois, F-54511 Vandoeuvre les Nancy – France

###### **Correspondence:** Collet C. – Université de Lorraine, F-54500 Vandoeuvre les Nancy – France

**Introduction:** The ^18^F-labeling of peptides for PET applications has been used for many years.^1^ However, the sensitivity of these peptides does not allow their direct radiolabelling under harsh conditions, except few recent examples. A solution is to use a prosthetic group, an easily radiolabeled small molecule, subsequently coupled in mild condition to the peptide. In continuation of our previous work,^2^ we therefore propose to develop and use new *C*-glycoside-based prosthetic groups. The use of sugar derivatives as prosthetic group would improve bioavailability and pharmacokinetic properties of peptides.

**Materials and methods:** These *C*-glycoside derivatives should have a good leaving group thus allowing easy substitution by fluorine-18, *i.e.* triflate. A copper catalyzed azide alkyne cycloaddition (CuAAC) was then used for coupling these carbohydrates with a peptide. Some model peptides containing a cysteine residue as RGDC and c(RGDfC) are used. The high nucleophilicity of the thiol function can thus be exploited to prepare *S*-propargylated derivatives. The fully automated radiosynthesis of these [^18^F]fluoro-glycopeptides was performed on an AllInOne® (Trasis) synthesizer.

**Results:** Triflated precursors of these *C*-glucosides prosthetic groups and the non-radioactive references were synthesized in alpha and beta configuration. Fluoride-18 radiolabeling was optimized and the automated radiosynthesis of [^18^F]fluoro-glycopeptides with some model peptides (RGDC, c(RGDfC)) was presented.

**Discussion/conclusion:** The synthesis and radiosynthesis of 6-[^19^F/^18^F]fluoro-*C*-glycosides displaying a three carbon arm terminated with an azide group were optimized. CuAAC of fluoro-*C*-glycosides with RGD derivative peptides gave [^19^F/^18^F]fluoro-glycopeptides in good yields.

### OP14 A Microfluidic Approach for the 68Ga-labeling of PSMAHBED-CC and NODAGA-RGD

#### Sarah Pfaff^1,2^, Cecile Philippe^1^, Markus Mitterhauser^1,3^, Marcus Hacker^1^, Wolfgang Wadsak^1,2^

##### ^1^Department of Biomedical Imaging and Image-guided Therapy, Division of Nuclear Medicine, Medical University of Vienna, Vienna, Austria; ^2^Department of Inorganic Chemistry, University of Vienna, Vienna, Austria; ^3^LBI for Applied Diagnostics, Vienna, Austria

###### **Correspondence:** Sarah Pfaff – Department of Biomedical Imaging and Image-guided Therapy, Division of Nuclear Medicine, Medical University of Vienna, Vienna, Austria

**Introduction:** In nuclear medicine a remarkably high demand of ^68^Ga-radiotracers has emerged during the last decade. For a variety of non-^68^Ga-containing radiotracers a microfluidic approach for their syntheses could be established, enabling an enhancement of yields due to high surface-to-volume ratios^1,2^. In this proof-of-principle study, the ^68^Ga-radiolabeling of PSMA^HBED-CC^ and NODAGA-RGD using a microfluidic approach was evaluated. Furthermore, adding TWEEN 20 (a surfactant suitable for *in vivo* applications) and its impact on the radiochemical yield was explored.

**Materials and methods:** The syntheses of ^68^Ga-PSMA^HBED-CC^ and ^68^Ga-NODAGA-RGD (both precursors from ABX) were performed using an Advion NanoTek LF microfluidic device. The system incorporates a flow-through reactor that consists of a silica capillary (l=2 m, Ø100 μm, V=15.6 μL). ^68^Ga^3+^ was obtained from a ^68^Ge/^68^Ga-generator (3.7 GBq; Obninsk) according to a fractionized protocol. The precursor and ^68^Ga^3+^ were loaded in two storage loops and distinct volumes thereof were pushed through the reactor with different flowrates (30, 50, 80 μL/min) at different temperatures (25, 50, 80, 100, 120, 150°C) using NaOAc and HEPES for pH adjustment, respectively. Additionally, the influence of TWEEN 20 as a surfactant, to reduce known adsorption effects in microfluidic tubing, was investigated.

**Results:** Accomplished experiments revealed feasibility of ^68^Ga-labeling of PSMA^HBED-CC^ and NODAGA-RGD using a microfluidic device. All temperatures and flowrates resulted in mean radiochemical yields in a range of 20-55%. Syntheses at higher temperatures and flowrates proved to be more efficient. For instance, HEPES buffered syntheses at 100°C and flowrate of 80 μL/min yielded ^68^Ga-PSMA^HBED-CC^ in 42.6 ± 22.1 % (n=10) and ^68^Ga-NODAGA-RGD in 49.7 ± 32.5 % (n=7). Applying the same parameters, TWEEN 20 could strikingly improve the yield of ^68^Ga-PSMA^HBED-CC^ to 70.8 ± 16.8 % (n=13).

**Discussion/conclusion:** This study provides the proof-of-principle of ^68^Ga-labeling in a microfluidic “flow-through” system. The application of TWEEN 20 led to drastically increased yields of ^68^Ga-PSMA^HBED-CC^ due to its surfactant nature. As a result, this microfluidic approach will be pursued to increase availability of ^68^Ga-radiopharmaceuticals according to the dose-on-demand principle.

### OP16 Surprising reactivity of astatine in the nucleophilic substitution of aryliodonium salts: application to the radiolabeling of antibodies

#### François Guérard^1^, Yong-Sok Lee^2^, Sébastien Gouard^1^, Kwamena Baidoo^3^, Cyrille Alliot^1,4^, Michel Chérel^1^, Martin W. Brechbiel^3^, Jean-François Gestin^1^

##### ^1^Centre de Recherche en Cancérologie Nantes-Angers (CRCNA), Unité INSERM 892 - CNRS 6299, Nantes 44007, France; ^2^Center for Molecular Modeling, Division of Computational Bioscience, Center for Information Technology, National Institutes of Health, Bethesda, Maryland 20892, USA; ^3^Radioimmune & Inorganic Chemistry Section, Radiation Oncology Branch, National Cancer Institute, National Institutes of Health, Bethesda, Maryland 20892, USA; ^4^Arronax GIP, Nantes 44817, France

###### **Correspondence:** François Guérard – Centre de Recherche en Cancérologie Nantes-Angers (CRCNA), Unité INSERM 892 - CNRS 6299, Nantes 44007, France

**Introduction:** Aryliodonium salts have recently emerged as versatile precursors for the synthesis of ^18^F-radiolabeled compounds for PET imaging.^1,2^ However, little is known about the applicability of these reagents for labeling with the heaviest radiohalogens iodine and astatine, both useful for nuclear imaging and/or therapy.^3^ In this study, we aimed at probing the reactivity of radio-iodide (^125^I) and astatide (^211^At) towards diaryliodonium salts in order to assess their usefulness for radiolabeling biomolecules of interest in nuclear medicine.

**Materials and methods:** First, parameters of radio-iodination and astatination reaction (solvent, temperature, duration and counter-ion of iodonium) were studied on model compounds. Bifunctional iodonium salts were then designed, allowing the synthesis of [^125^I]-SIB and [^211^At]-SAB, two prosthetic groups widely used for radio-iodination and astatination of biomolecules. Both [^125^I]-SIB and [^211^At]-SAB were conjugated to the multiple myeloma targeting mAb 9E7.4 (anti-CD138). Conjugation yields and resulting immunoreactivity were compared with the conventional arylstannane chemistry approach.

**Results:** Initial reaction parameters studies highlighted a striking difference of reactivity between radio-iodide and astatide that could not be anticipated from the trends observed within the halogen series. Not only the astatination reaction was highly efficient at much lower temperature than iodination, but it appeared also solvent and counter-ion independent (not iodination). Thermochemical studies highlighted a large difference of activation energy in acetonitrile between both halogens with *Ea* = 23.5 kcal/mol and 17.2 kcal/mol for radio-iodination and astatination, respectively. Quantum chemical calculations support the hypothesis that astatination occurs via a monomeric iodonium complex whereas iodide occurs via a dimeric complex which requires more energy for the reaction to proceed. This explains the large reactivity difference observed. Radiolabeling of an antibody with specifically designed iodonium salts outperformed conventional arylstannane chemistry approaches in terms of global efficiency (radiochemical yields >90%, conjugation yields ≈ 75%, and simpler purification: no HPLC needed) with excellent preservation of immunoreactivity of the IgG with both radionuclides and less concerns regarding the toxicity of precursors and side products.

**Discussion/conclusion:** In comparison with the conventional arylstannane approach, aryliodonium salts appear as more efficient precursors for preparation of radio-iodinated and astatinated compounds. Furthermore, they allow simpler purification procedures (easy transfer to automation), with the additional advantage of a much lower toxicity, which is of primary importance for human use. Most of all, the unexpected reactivity of astatine we unveiled highlights that a lot is still to be discovered about the chemistry of this radioelement which remains to date largely unexplored.

### OP17 ^64^Cu-NOTA-pertuzumab F(ab')_2_ fragments, a second-generation probe for PET imaging of the response of HER2-positive breast cancer to trastuzumab (Herceptin)

#### Lam K^1^, Chan C^1^, Reilly RM^1,2,3^

##### ^1^Department of Pharmaceutical Sciences, University of Toronto, Toronto, Canada; ^2^Department of Medical Imaging, University of Toronto, Toronto, Canada; ^3^Toronto General Research Institute, University Health Network, Toronto, Canada

###### **Correspondence:** Lam K – Department of Pharmaceutical Sciences, University of Toronto, Toronto, Canada

**Introduction:** SPECT/CT imaging with ^111^In-BzDTPA-pertuzumab detected trastuzumab (Herceptin)-mediated HER2 downregulation in human breast cancer xenografts in mice and was correlated with a good response to treatment. This agent is now being studied in a Phase I/II clinical trial sponsored by OICR (PETRA; ClinicalTrials.gov identifier: NCT01805908). Our objective was to develop and characterize a second-generation positron-emitting analogue for PET/CT imaging, ^64^Cu-NOTA-pertuzumab F(ab')_2_, to provide greater sensitivity, more accurate radiotracer quantitation and a lower radiation absorbed dose.

**Materials and methods:** To determine the optimal dose, mice with subcutaneous HER2-overexpressing tumours were injected with 5, 50, 100 or 200 μg of ^64^Cu-NOTA-F(ab')_2_ (2.2±0.6 MBq) and sacrificed at 24 h p.i. for biodistribution. To determine the normal tissue distribution, pharmacokinetics and radiation dosimetry of ^64^Cu-NOTA-F(ab')_2_, non-tumour bearing Balb/c mice were administered 50 μg of ^64^Cu-NOTA-F(ab')_2_ (2.9±0.3 MBq) and sacrificed at select time points. Three groups of mice bearing HER2-overexpressing tumours were injected with ^64^Cu-NOTA-F(ab')_2_ (50 μg; 10.6±0.4 MBq) with or without administration of pertuzumab (1 mg) 24 h before, or with ^64^Cu-NOTA-F(ab')_2_ prepared from nonspecific human IgG (50 μg; 8.2±1.9 MBq) to demonstrate specificity. Mice were imaged with PET/CT at 24 and 48 h p.i. and sacrificed for biodistribution.

**Results:** The 50 μg dose of ^64^Cu-NOTA-F(ab')_2_ showed the highest tumour to blood ratio, followed by the 100, 5, and 200 μg doses (18.2±7.2, 15.4±0.8, 14.0±5.0, and 10.9±1.8, respectively). In non-tumour bearing mice, only the kidney retained significant radiotracer uptake at 24 h p.i. (49.1±5.9 %ID/g). Initial estimates for *t*_*½*_ *α* and *t*_*½*_ *β* were 1.2 h and 6.0 h, respectively. The projected total body radiation absorbed dose to humans was 0.02 mSv/MBq, half that estimated for ^111^In-BzDTPA-pertuzumab. Tumour to normal tissue contrast of PET/CT images appeared similar between the 24 and 48 h p.i. imaging time points. Tumour accumulation of ^64^Cu-NOTA-F(ab')_2_ was significantly higher in mice administered ^64^Cu-NOTA-F(ab')_2_ without pertuzumab blocking (8.4±3.4 %ID/g) relative to mice pre-injected with 1 mg of pertuzumab (3.9±0.5 %ID/g; *P* < 0.05), and mice administered ^64^Cu-NOTA-F(ab')_2_ prepared from non-specific human IgG (2.7±0.5 %ID/g; *P* < 0.05).

**Discussion/conclusion:** ^64^Cu-NOTA-F(ab')_2_ is HER2 specific and can visualize HER2-overexpressing tumours at 24 or 48 h p.i. with PET/CT imaging. The lower projected radiation absorbed doses for ^64^Cu-NOTA-F(ab')_2_ compared to ^111^In-BzDTPA-pertuzumab may make this a more attractive imaging agent to detect trastuzumab-mediated HER2 downregulation in breast cancer. Supported by grants from the OICR Smarter Imaging and High Impact Clinical Trials (HICT) Programs.

### OP18 Development of radiohalogenated analogues of a avb6-specific peptide for high LET particle emitter targeted radionuclide therapy of cancer

#### Salomé Paillas^1^, John Marshall^2^, Jean-Pierre Pouget^3^, Jane Sosabowski^1^

##### ^1^Centre for Molecular Oncology, Barts Cancer Institute, Charterhouse Square, Queen Mary University of London, London, UK; ^2^Centre for Tumour Biology, Barts Cancer Institute, Charterhouse Square, Queen Mary University of London, London, UK; ^3^Institut de Recherche en Cancérologie de Montpellier, Inserm U1194, Montpellier, France

###### **Correspondence:** Salomé Paillas – Centre for Molecular Oncology, Barts Cancer Institute, Charterhouse Square, Queen Mary University of London, UK

**Introduction:** Targeted radionuclide therapy (TRT) of solid tumors has a limited efficacy mainly because these tumours have high radioresistance and take up limited amounts of radiolabeled vectors. Strategies to overcome this include the use of small peptides combined with radionuclides that emit highly cytotoxic particles, namely high linear energy transfer (LET) particles such as alpha particles and Auger electrons. We have chosen to target the epithelial-specific integrin αvβ6, which is weak or absent on normal tissues but is upregulated on many cancers where it is strongly associated with reduced survival. One targeting vector is a 20 mer peptide, A20FMDV2 which we have previously used to image αvβ6-positive tumours with SPECT (In-111) or PET (F-18, Ga-68). The peptide showed extremely high kidney retention when radiolabelled with radiometals, but this was not seen with the F-18 analogue. Due to anticipated dose-limiting toxicity in kidney for radiometal-based TRT, we have developed a peptide suitable for radiohalogenation with the Auger electron emitter I-125 and the alpha particle emitter At-211.

**Materials and methods:** The 20 mer high affinity αvβ6-targeting peptide, A20FMDV2 used in previous work has been derivatised to contain a trimethylstannyl benzamide moiety so that it can be radiolabelled with I-125 and At-211. The peptide has been radiolabelled with I-125 and radioligand binding and internalization assays have been carried out in cells that overexpress αvβ6 and compared with that of the 111In-DTPA-A20FMDV2 studied previously. Clonogenic assays have been carried out on both the non-radiolabelled peptide and the radiolabelled analogue in both αvβ6-positive and negative cells to determine the effect of high LET irradiation.

**Results:** Radiolabelling efficiency with I-125 was >91 % and the analogue showed high binding affinity and rapid internalisation. Clonogenic assays showed that the non-labelled peptide alone was able to inhibit cell growth and that this effect was not seen on αvβ6-negative cells. This effect was enhanced when radiolabelled with a high LET particle emitter.

**Discussion/conclusion:** The αvβ6-specific peptide when radiolabelled with a high-LET particle emitter shows promise as an agent for targeted radionuclide therapy.

### OP19 Ligand Specific Efficiency (LSE) as a guide in tracer optimization

#### Emmanuelle Briard^1^, Yves P. Auberson^1^, John Reilly^2^, Mark Healy^2^, David Sykes^3^

##### ^1^Novartis Institutes for Biomedical Research, Basel, Switzerland; ^2^Novartis Institutes for Biomedical Research, Cambridge, USA, ^3^University of Nottingham, Nottingham, UK

###### **Correspondence**: Emmanuelle Briard – Novartis Institutes for Biomedical Research, Basel, Switzerland

**Introduction:** Successful radiotracers result from a favorable combination of target density, ligand affinity, nonspecific binding and permeation. All these parameters can be measured independently and the interplay between some of them is well known: for instance, Bmax/Kd > 10. Importantly, the required affinity of a tracer is also correlated to nonspecific binding: An increased affinity is beneficial only if the nonspecific binding remains constant. This work aimed at identifying an index taking into account the relationship between these two parameters, to guide optimization from the early stage of tracer development projects.

**Materials and methods:** Similarly to the Ligand Efficiency (LE) index, we explored the usefulness of the Ligand Specific Efficiency index (LSE), which we defined as the ratio between affinity (expressed as e.g. pIC_50_ or pK_d_), and the logarithmic value of the experimental non-specific binding measurement, CHI(IAM).^[Jiang]^ LSE provides a measure of affinity, normalized to non-specific binding. It shows how efficient the ligand is at binding to the desired target, compared to all other non-specific binding partners.

**Results:** A series of well-described PET tracers was evaluated to set the LSE threshold. This analysis showed that an LSE > 5 and preferably LSE > 5.4 is required for a successful tracer. This concept was applied to the development of a prostacyclin receptor (IPR) tracer. Our chemical starting point was Ro1138452,^[Clark]^ which we selected based on encouraging overall properties, including a high affinity for IPR (Ki = 0.23 nM). In contrast, its CHI(IAM) value of 58 clearly indicated a high tendency for non-specific binding. Despite such high non-specific binding, Ro1138452 has a LSE value of 5.1, which is close to the minimum value that would be expected for a successful PET tracer. This raised hope that some improvement in binding specificity would allow the use of a close derivative for imaging purposes. The use of LSE during the IPR tracer optimization will be presented.

**Discussion/conclusion:** LSE is based on a rather intuitive concept, in the sense that a good PET tracer candidate should have the optimum balance of affinity and binding specificity. It is a convenient index to evaluate and compare molecules based on measured, rather than *in silico* values, and is applicable independently of target and chemotype. It is a useful index from the beginning of a project, facilitates the selection of the most promising scaffold and guides their optimization.

### OP23 The radiosynthesis of an 18F-labeled triglyceride, developed to visualize and quantify brown adipose tissue activity

#### Andreas Paulus^1^, Wouter van Marken Lichtenbelt^1^,Felix Mottaghy^2,3^, Matthias Bauwens^1,3^

##### ^1^Research School NUTRIM, Maastricht University, Maastricht, Netherlands; ^2^Division of Nuclear Medicine, Uniklinikum Aachen, Aachen, Germany; ^3^Department of Medical Imaging, Division of Nuclear Medicine, MUMC, Maastricht, Netherlands

###### **Correspondence:** Matthias Bauwens – Research School NUTRIM, Maastricht University, Maastricht, Netherlands

**Introduction:** Brown adipose tissue (BAT) is an interesting type of tissue that receives major interest as a target to combat obesity. It was (re-)discovered in 2009, when PET-CT studies with [^18^F]-FDG showed its presence and metabolic activity even in adult humans [1]. It is however difficult to quantitatively assess this metabolic activity using [^18^F]-FDG, as BAT mainly uses lipids as an energy source. Radiolabeled fatty acids, such as [^18^F]-FTHA, can provide useful information, but are not optimal considering the lipid uptake mechanism BAT is not based on singular fatty acids, but instead triglycerides (in lipoproteins). As such, we aim to develop a radiolabeled triglyceride, hoping this will shed more light on the lipid burning of BAT, thus allowing to calculate the impact of BAT on the total metabolism of the human body.

**Methods:** A fatty acid (C_16_) BODIPY-Fl dye is radiolabeled with F-18 using an F-18/F-19 exchange reaction of the boron-fluoride core of the BODIPY dye to yield a bimodal PET/fluorescent imaging tool. BODIPY-C_16_ is esterified with 1,3 – Diolein and assistance of thionylchloride yielding the resulting fluorescent triglyceride (TG). *In vitro* experiments with BODIPY-C_16_ are conducted to investigate the applicability the dual-modality imaging probe.

**Results:** BODIPY-C_16_ could be radio-labeled in a Lewis Acid assisted F-18/F-19 exchange reaction in a reasonable yield (66%, not corrected for decay) and a final purity after C18 Sep-Pak purification of more than 97%. Esterification of BODIPY-C_16_ resulted in a BODIPY-TG with a yield of more than 90% within 30 minutes. First *in vitro* experiments showed specific uptake of BODIPY-C_16_ in BAT and WAT as the compound was located within the lipid droplets of the cell. Direct radiolabeling of the BODIPY-TGL was also successful (60% yield, >97% purity).

**Discussion/conclusion:** First experiments implied radiolabeled BODIPY-C_16_ is a promising BAT PET tracer with the opportunity to resolve its metabolic character on a sub-cellular level due to its dual-modality. In vivo imaging of radiolabeled BODIPY-C_16_ and BODIPY-TGL, incorporation into micelles as well as further *in vitro* experiments will be conducted in the near future to investigate the applicability of this tracer in lipid metabolism imaging of BAT.

### OP24 Influence of the fluorescent dye on the tumor targeting properties of dual-labeled HBED-CC based PSMA inhibitors

#### Baranski, Ann-Christin^1^, Schäfer, Martin^1^, Bauder-Wüst, Ulrike^1^, Haberkorn, Uwe^2^, Eder, Matthias^1^, Kopka, Klaus^1^

##### ^1^Division of Radiopharmaceutical Chemistry, German Cancer Research Center (dkfz), Heidelberg, Germany; ^2^Department of Nuclear Medicine, University of Heidelberg, Heidelberg, Germany

###### **Correspondence:** Baranski, Ann-Christin **–** Division of Radiopharmaceutical Chemistry, German Cancer Research Center (dkfz), Heidelberg, Germany

**Introduction:** Image-guided cancer surgery using fluorescence imaging has high clinical impact and already shows potential to improve the outcome of oncological surgery (1). As first clinical experiences with the ^68^Ga-labeled PSMA-targeting inhibitor Glu-urea-Lys-Ahx-HBED-CC (PSMA-11) demonstrated high and specific tracer uptake in prostate cancer lesions a fluorescence dye conjugate of PSMA-11 might represent a promising bimodal tracer. The combination of preoperative staging by means of PET/CT and PET/MRI, followed by image-guided surgery will further improve the accuracy of detecting PSMA-positive tumor lesions by merging the strengths of both techniques. Therefore, various fluorescent dyes were conjugated to the inhibitor PSMA-11 to determine the impact of the dye conjugation on the ligand’s in vitro and in vivo characteristics.

**Materials and methods:** The optical-dye labeled tracer PSMA-HBED-CC-FITC, PSMA-HBED-CC-AlexaFluor488 and PSMA-HBED-CC-IRDye800CW were synthesized based on PSMA-11. The binding properties were analyzed in a competitive cell binding assay followed by internalization experiments in human PSMA expressing LNCaP cells. Biodistribution studies were performed in LNCaP tumor-bearing mice (BALB/c nu/nu) to determine specific tumor uptake and pharmacokinetic properties.

**Results:** Comparative cell binding experiments revealed a high affinity to PSMA expressing cell lines for all conjugates, which is in line with the values obtained with the reference ^68^Ga-PSMA-11. The radiolabeled fluorescent-dye conjugates showed specific cell uptake and were effectively internalized into the PSMA expressing cell line LNCaP. First in vivo results indicated slightly varying pharmacokinetic properties depending on the fluorescent dye. The FITC- and AlexaFluor488-conjugates revealed a higher tumor uptake compared to ^68^Ga-PSMA-11, while a minor, but still satisfying uptake was detected for the IRDye800CW-conjugate.

**Discussion/conclusion:** Conjugation of a fluorescent dye to the well-established imaging agent PSMA-11 showed rather minor dye-dependent impact on cell binding properties, tumor uptake and the pharmacokinetic characteristics. In order to further improve the biodistribution profile of the IRDye800CW-conjugate, structural optimization will be done. These first preclinical results emphasize the potential of a dual-labeled PSMA inhibitor to serve as a multimodal imaging agent, enabling sensitive pre-, intra- and post-therapeutic identification of metastases with one and the same molecule.

### OP25 [18F]MEL050 as a melanin PET tracer : fully automated radiosynthesis and evaluation for the detection of pigmented melanoma in mice pulmonary metastases

#### Chaussard M^1,2^, Hosten B^1,2,4^, Vignal N^1,2,4^, Tsoupko-Sitnikov V^1,2^, Hernio N^1,5^, Hontonnou F^1,5^, Merlet P^1,2,5^, Poyet JL^3,5^, Sarda-Mantel L^1,2,5^, Rizzo-Padoin N^1,2,4^

##### ^1^Unité Claude Kellershohn, IUH, Hôpital Saint-Louis, Paris, F-75010, France; ^2^GH Saint-Louis Lariboisière F. Widal AP-HP, Paris, F-75010, France; ^3^Inserm U1160, Hôpital Saint-Louis, Paris, F-75010 France; ^4^Université Paris Descartes, Faculté de Pharmacie, Paris, F-75006, France; ^5^Université Paris Diderot, Faculté de médecine, Paris, F-75010, France

###### **Correspondence:** Chaussard M – Unité Claude Kellershohn, IUH, Hôpital Saint-Louis, Paris, F-75010, France

**Introduction**: Melanoma is a highly malignant cutaneous tumor of melanin-producing cells. Early detection of melanoma is the best way to reduce mortality. Several radiolabeled imaging probes have been evaluated for melanoma imaging. MEL050 is a synthetic benzamide-derived molecule that specifically binds to melanin with high affinity. Our aim was to implement a fully automated radiosynthesis of [^18^F]MEL050, including HPLC purification and formulation, using for the first time, the AllInOne radiosynthesis platform (Trasis, Ans, Belgium), and to validate this PET radiotracer *in vivo* in a mouse model of melanoma.

**Materials and methods**: [^18^F]MEL050 was synthesized using a one-step bromine-for-fluorine nucleophilic heteroaromatic substitution. Briefly, [^18^F]MEL050 was prepared from a bromo-precursor, using no-carrier-added 18F-KF-Kryptofix 222 (dimethylformamide, 150°C, 6 min), followed by preparative HPLC purification (C18 column (300x7.8 mm, 7 μm), isocratic elution with acetonitrile/20 mM ammonium bicarbonate (20:80, v/v), 3.0 ml/min) and cartridge-reformulation (Sep-Pak® Plus C18 environmental) of the collected fraction. Radiochemical and chemical purity, stability and specific activity measurements were monitored using analytical HPLC. Chemical identity of the labeled compound [^18^F]MEL050 was assessed by co-injection with non-radioactive standard MEL050. Experimental model of pulmonary metastatic melanoma was obtained by IV injection of B16-F10 cells in NMRI mice. Mice (n=8) were imaged 15 days after inoculation, using INVEON microPET/CT device (Siemens), after IV injection of 0.36±0.04MBq/g of [^18^F]MEL050. Dynamic and static acquisitions were acquired from time of injection to 2h after tracer injection. The maximum percentage of [^18^F]MEL050 Injected Dose per g of lung tissue (%ID/g Max) was determined using ROIs manually drawn on 1h-post injection PET images, and correlated to *ex-vivo* findings.

**Results:** The fully automated radiosynthesis of [^18^F]MEL050 required an overall radiosynthesis time of 60 min, with an end-of-synthesis yield of 20-26% (n=12). Isocratic semi-preparative HPLC allowed efficient separation of [^18^F]MEL050 from the reaction mixture. The radiotracer was consistently produced with radiochemical purity higher than 99%. The specific activity was in the range of 177-325GBq/μmol and the product stability was maintained at RCP>98% over 6 h. PET/CT images retrieved known biodistribution of [^18^F]MEL050 in mice, and allowed clear visualization of <1mm lung tumours with [^18^F]MEL050 %/ID/g Max of 4.7±2.6%.

**Discussion/conclusion:** We successfully implemented the radiosynthesis of [^18^F]MEL050 using the AllInOne radiosynthesis platform, including HPLC separation and formulation. *In vivo* PET/CT validation of the radiotracer was obtained in a mouse model of metastatic pigmented melanoma, showing high specific [^18^F]MEL050 uptake in sub-millimetric lung tumours.

### OP26 Design and Preclinical Evaluation of Novel Radiofluorinated PSMA Targeting Ligands Based on PSMA-617

#### J. Cardinale^1^, M. Schäfer^1^, M. Benešová^1^, U. Bauder-Wüst^1^, O. Seibert^2^, F. Giesel^2^, U. Haberkorn^2^, M. Eder^1^, K. Kopka^1^

##### ^1^DKFZ, Division of Radiopharmaceutical Chemistry, Heidelberg, Germany; ^2^DKFZ, Clinical Cooperation Unit Nuclear Medicine, Heidelberg, Germany

###### **Correspondence**: J. Cardinale – DKFZ, Division of Radiopharmaceutical Chemistry, Heidelberg, Germany

**Introduction:** Urea-based inhibitors of the prostate-specific membrane antigen (PSMA) are well known and promising candidates for the diagnosis (1, 2) and therapy of prostate cancer. The aim of the project was the development of F-18 labeled PSMA ligands based on the theranostic compound PSMA-617. The compounds evaluated during the preliminary experiments showed a high uptake in non-target organs caused by their relatively high lipophilicity. Therefore we currently reduce this lipophilicity by the addition of charged amino acids to the linker region of our PSMA inhibitors (3).

**Materials and methods:** The PSMA binding motif Glu-NH-CO-NH-Lys was synthesized by a well-established method (4) using solid phase chemistry and subsequently an amino acid linker was built up by fmoc-based solid phase peptide synthesis (SPPS). The non-radioactive reference compounds were also prepared analogically and eventually conjugated by 6-fluoronicotinic acid. After separation from the resin and deprotection the peptidomimetics were labeled using the TFP-ester of 6-[^18^F]fluoronicotinic acid as prosthetic group. The K_i_ values of all compounds were determined by competitive binding assays on PSMA-positive LNCaP cells against ^68^Ga-PSMA-10 (5) using the respective cold reference compounds. Additionally, the cellular internalization of the radiofluorinated ligands was determined.

**Results:** All ^18^F-labeled PSMA-inhibitors presented in this study showed low nanomolar affinities towards PSMA, usually paired with high internalization ratios of more than 15 %, shown *in vitro*. Among those PSMA-1007 showed an outstandingly high internalization ratio of about 70 % while the *K*_*i*_ was in the typical range (6 nM). Hence PSMA-1007 was further evaluated *in vivo*. The organ distribution showed a high and specific tumor uptake of 8.0±2.4 %ID/g. Finally, the PSMA-targeting potential of PSMA-1007 was further demonstrated by dynamic μPET experiments.

**Discussion/conclusion:** Based on our experience with PSMA-617 and preliminary results we developed a series of radiofluorinated PSMA inhibitors with high affinities and internalization ratios. Among those a promising candidate for further translation into the clinic has already been found. This candidate – namely PSMA-1007 – is currently transferred into first-in-man studies. Nevertheless further optimization of the lead compound is still ongoing.

### OP27 A novel radiolabeled peptide for PET imaging of prostate cancer: 64Cu-DOTHA2-PEG-RM26

#### Mansour Nematallah^1^, Paquette Michel^1^, Ait-Mohand Samia^1^, Dumulon-Perreault Véronique^2^, Lecomte Roger^1,2^, Guérin Brigitte^1,2^

##### ^1^Département de médecine nucléaire et radiobiologie, Faculté de médecine et sciences de la santé, Université de Sherbrooke, QC, Canada J1H5N4; ^2^Centre d’Imagerie Moléculaire de Sherbrooke (CIMS), CR-CHUS, Sherbrooke, QC, Canada J1H5N4

###### **Correspondence:** Guérin Brigitte – Département de médecine nucléaire et radiobiologie, Faculté de médecine et sciences de la santé, Université de Sherbrooke, QC, Canada J1H5N4

**Introduction:** The Gastrin-Releasing Peptide Receptor (GRPR) is overexpressed in a wide variety of prostate cancers, hence its interest as a potential biomarker. As such, previous work used different radiolabeled GRPR-binding peptides to specifically target tumors *in vivo* (1, 2). Recently, we synthesized a novel bifunctional chelator bearing hydroxamic acid arms, called DOTHA_2_, for which our group demonstrated fast and stable complexation to ^64^Cu (3). The goal of this study was to develop a GRPR antagonist, D-Phe-Gln-Trp-Ala-Val-Gly-His-Sta-Leu-NH_2_ (RM26), conjugated to the novel [^64^Cu]-DOTHA_2_ to visualize prostate tumor by PET imaging.

**Material and methods:** DOTHA_2_-PEG-RM26 was synthesized on solid support. The inhibition constant (*K*_i_) was measured on PC3 cells. Dynamic PET images were acquired during 60 minutes after bolus administration on nu/nu male mice bearing PC3 tumors. Biodistribution studies were performed at different time points, using balb/c male mice and nu/nu male mice bearing PC3 tumors. To assess specific binding, a cohort received 500 nmol/kg of unlabeled peptide 15 minutes prior tracer injection for both PET and dissection studies.

**Results:** The *K*_i_ of Cu-DOTHA_2_-PEG-RM26 was in the low nanomolar range (0.68 nM). DOTHA_2_-PEG-RM26 complexed ^64^Cu with fast kinetics at room temperature. The radiopeptide showed high stability, low residual activity in various tissues and fast clearance in normal mice. Small animal blocking experiments showed a significant uptake drop compared to tracer by biodistribution in the GRPR-rich pancreas for both balb/c and nu/nu mice (respectively p< 0.05 and p < 0.01 at 30 min). A significant uptake decrease from 5.3±0.8%ID/g to 3.4±1.4%ID/g was also observed in PC3 tumors when unlabeled peptide was added in biodistribution experiments at 30 minutes, whereas a significantly reduced tumor uptake was also assessed by PET imaging from 20 to 60 minutes (p<0.05) post-injection.

**Discussion/conclusion:** The use of [^64^Cu]-DOTHA_2_-PEG-RM26 is promising for visualizing prostate tumors. These preliminary data suggest that DOTHA_2_ can be used to develop many other peptide- and protein-derived PET tracers.

### OP29 Biodistribution of [^18^F]Amylovis®, a new radiotracer PET imaging of β-amyloid plaques

#### Fernandez-Maza L^1^, Rivera-Marrero S^2^, Prats Capote A^3^, Parrado-Gallego A^1^, Fernandez-Gomez I^1^, Balcerzyk M^1^, Sablon-Carrazana M^2^, Perera-Pintado A^3^, Merceron-Martinez D^2^, Acosta-Medina E^4^, Rodriguez-Tanty C^2^

##### ^1^Centro Nacional de Aceleradores. Universidad de Sevilla, CSIC, Junta de Andalucia, Sevilla, Spain; ^2^Centro de Neurociencias (CNEURO), La Habana, Cuba; ^3^Centro de Isótopos (CENTIS), Mayabeque, Cuba; ^4^Centro de Estudios Avanzados de Cuba (CEAC), La Habana, Cuba

###### **Correspondence:** Fernandez-Maza L – Centro Nacional de Aceleradores. Universidad de Sevilla, CSIC, Junta de Andalucia, Sevilla, Spain

**Aim:** [^18^F]-2-(3-fluoropropyl)-6-methoxynaphtalene ([^18^F]Amylovis®) is a new naphthalene-derivative for detecting β-amyloid plaques in Alzheimer’s disease. The aim of the study is the assessment of the animal biodistribution of this new radiotracer.

**Material and methods:** [^18^F]Amylovis® was synthesized by nucleophilic substitution of the tosyl group of the precursor. Thirty five healthy male Balb/C mice of 10-12 weeks were divided into 6 groups of 5 animals each and injected with similar doses of [^18^F]Amylovis® through a lateral tail vein. Blood samples were collected and the animals were sacrificed at 5, 15, 30, 45, 70 and 180 minutes. Organs of interest were removed and washed with saline. Radioactivity of blood, plasma, urine, faeces, brain, cerebellum, heart, liver, stomach, spleen, bowel, colon, left kidney, muscle, bone and tail was measured in a well counter. To assess protein binding, plasma samples were diluted with acetonitrile and centrifuged at 4000 g. Pellets of proteins and supernatants were separated and their radioactivity measured in a well counter. RadioTLC analysis of plasma were performed for the same purpose in silicagel 60 and mobile phase of acetonitrile/water (95/5). 20μL of each supernatant was analysed by HPLC-RP using a C18 column and acetonitrile/water (75/25) as mobile phase to identify plasma metabolites. Pharmacokinetic parameters (AUC, t_1/2_, C_max_, Cl, V_ss_) were calculated using non-compartmental analysis (NCA). Dynamic PET/CT images of healthy and transgenic APPSwe/PS1dE9 mice were acquired for 2.5 h after i.v. administration. Immunohistochemistry of control and transgenic mice brains were performed to identify β-amyloid plaques.

**Results:** [^18^F]Amylovis® crossed blood brain barrier. PET/CT images showed significant differences between healthy and transgenic mice, expressed in Cortex to cerebellum SUV ratio, with maximum difference at 30 minutes. *Postmortem* studies of immunohistochemistry showed also differences in healthy vs transgenic mice (amyloid positive). Plasma T_1/2_ of 37 min. No significant protein binding was observed. Renal and hepatic pathways were the main excretion routes. Some amount of *in vivo* degradation metabolites appeared in blood from 1 h post-administration.

**Conclusion**: [^18^F]Amylovis® could be a promising PET radiotracer for amyloid plaques visualization.

### OP30 Synthesis and preclinical evaluation of [11C]-BA1 PET tracer for the imaging of CSF-1R

#### Bala Attili, Muneer Ahamed, Guy Bormans

##### Laboratory for Radiopharmacy, Department of Pharmaceutical and Pharmacological Sciences, KU Leuven, Leuven, Belgium

###### **Correspondence:** Bala Attili – Laboratory for Radiopharmacy, Department of Pharmaceutical and Pharmacological Sciences, KU Leuven, Leuven, Belgium

**Introduction:** Colony stimulating factor-1R (CSF-1R) is also called as Feline McDonough Sarcoma (FMS), is a type-III kinase receptor. FMS widely expressed and considered to regulate development, maintenance and functioning of mononuclear phagocyte lineage such as monocytes, *macrophages*, dendritic cells, langerhans cells, *microglia* and osteoclasts^1^. Over expression of FMS have been implicated in number of disease states including cancer, rheumatoid arthritis, Crohn’s disease and Bone disorders. FMS also known to play a key role in microglia differentiation and activation and assuming that it was key mediator in neuroinflammatory process.

**Materials and methods:** All the chemicals and reagents used in the experiments were obtained from commercial sources and used without any further purification. Synthesis of 5-cyano-N-(4-(4-methylpiperazine-1-yl)-2-(4-methylpiperidin-1-yl)phenyl)furan-2-carboxamide and 5-cyano-N-(2-(4-methylpiperidine-1-yl)-4-(piperazine-1-yl)phenyl)furan-2-carboxamide molecules were done according to literature methods available with slight modifications for radiolabeling experiments^3^. The [^11^C]-methyl triflate reacts with the precursor in presence of a base at room temperature for 2 minutes gives the carbon-11 radiolabeled [^11^C]BA-1 which was purified by using semi-preparative reversed-phase high pressure liquid chromatography (RP-HPLC). The peak corresponding the reference compound will be collected and checked for the purity using analytical RP-HPLC. Baseline biodistribution study was performed at 2, 10, 30 and 60 min. respectively. Blocking experiment (10 mg/kg cold compound) was performed at 30 min time point in healthy female adult mice n=3 and compared with vehicle treated batch. In vitro autoradiography experiments were carried on rat brain slices by incubating tracer and cold blocking solution. MicroPET imaging studies were performed on a Focus™ 220 microPET scanner with female rats.

**Results**: Reference and precursor molecules were synthesized with comparable purities and yields reported in the literature, the radiochemical yield (Alkylation yield with [11C]CH3I) 60 % and radiochemical purity of 98 % and specific activity (n=5) 247.3 GBq/μmol. Biodistribution study shows higher tracer uptake into the brain % ID 4 at 2 min time point, main route of excretion via renal and hepatobiliary circulation. High lung uptake was observed with % ID 14 at 2 min. Blocking with cold compound did not observe any blocking effect in brain but we observed blocking in peripheral organs like liver and spleen, also observed slight blocking in pancreas and kidneys. We did not observed any blocking with in vitro autoradiography experiments. Baseline microPET scans suggests good uptake of tracer into brain with SUV 1.2 but with blocking we did not observed any blocking effect despite we observe higher uptake in brain with SUV 2.

**Discussion/conclusion:** We successfully synthesized, [^11^C]-BA1. In preclinical evaluation we did not observe any significant blocking effect in the brain, we are currently looking for other high affinity molecules for CSF-1R.

### OP31 In vivo imaging of the MCHR1 in the ventricular system via [18F]FE@SNAP

#### C. Philippe^1^, M. Zeilinger^1^, T. Scherer^2^, C. Fürnsinn^2^, M. Dumanic^1^, W. Wadsak^1^, M. Hacker^1^, M. Mitterhauser^1,3^

##### ^1^Department of Biomedical Imaging and Image-guided Therapy, Division of Nuclear Medicine, Radiopharmacy and Experimental Nuclear Medicine, Medical University of Vienna, Vienna, Austria; ^2^Department of Medicine III, Division of Endocrinology and Metabolism, Medical University of Vienna, Vienna, Austria; ^3^Ludwig Boltzmann Institute for Applied Diagnostics, Vienna, Austria

###### **Correspondence:** C. Philippe – Department of Biomedical Imaging and Image-guided Therapy, Division of Nuclear Medicine, Radiopharmacy and Experimental Nuclear Medicine, Medical University of Vienna, Austria

**Introduction:** The melanin concentrating hormone receptor 1 (MCHR1) is predominately expressed in the lateral hypothalamus and is playing a key role in energy homeostasis and obesity. Recently, it has been shown that the MCHR1 is expressed in the ependymal cells of the 3^rd^ ventricle where it is involved in the regulation of cilia beat frequency [1]. This beating facilitates cerebrospinal fluid circulation, which is crucial for brain function, as defects in ventricular cilia result in hydrocephalus. Our aim was to investigate the potential of the MCHR1 ligand [^18^F]FE@SNAP [2] for PET-imaging of the MCHR1 in the ventricular system.

**Materials and methods:** *In vivo* experiments were conducted in naïve male Sprague Dawley rats. For small-animal PET/CT/MR experiments, rats were anesthetized by isoflurane. 25min after [^18^F]FE@SNAP iv injection (47.8±1MBq; SA: 19.7±6GBq/μmol), vehicle (baseline group, n=3) or 15mg/kg SNAP-7941 (blocking group, n=3) were administered through the tail vein. 75min after tracer injection, the rats were sacrificed. Radioactivity concentrations in brain were calculated and expressed as SUVs. In another set of experiments, [^18^F]FE@SNAP (51.3±26MBq; SA: 36.1±28GBq/μmol) as well as vehicle (baseline group, n=3) or 15mg/kg SNAP-7941 (blocking group, n=3) were injected into conscious freely moving rats via a permanent catheter implanted into the jugular vein, thus excluding an influence of anaesthesia. 45min after tracer application, rats were sacrificed, the brain was removed and cut for *ex vivo* autoradiography. Subsequently, brain slices were put on Phosphor Imager plates for exposure and analyzed with a Cyclone Phosphor Imager the following day. Regions of interest (ROIs) were drawn for the hypothalamic region, the ventricle and a non-target region. ROIs resulted in normalized DLU/mm^2^); ratios of ventricle/non-target were calculated.

**Results:** PET/CT/MR experiments: the SUV in the ventricular system was 0.73±0.11 for the baseline group and 0.34±0.07 for the blocking group, which represents a significant reduction. *Ex vivo* autoradiography showed a distinct uptake in the ventricular, which was significantly reduced in the blocking group.

**Conclusion:** [^18^F]FE@SNAP evinced specific uptake in the ventricular system of naïve rats regardless their state of consciousness and is therefore a suitable imaging agent for cilia beat function.

### OP32 Synthesis of the first carbon-11 labelled P2Y12 receptor antagonist for imaging the anti-inflammatory phenotype of activated microglia

#### B. Janssen^1^, D.J. Vugts^1^, G.T. Molenaar^1,2^, U. Funke^1,2^, P.S. Kruijer^2^, F. Dollé^3^, G. Bormans^4^, A.A. Lammertsma^1^, A.D. Windhorst^1^

##### ^1^Department of Radiology & Nuclear Medicine, Neuroscience Campus Amsterdam, VU University Medical Center, Amsterdam, The Netherlands; ^2^BV Cyclotron VU, Amsterdam, The Netherlands; ^3^CEA, Institut d’Imagerie BioMédicale, Service Hospitalier Frédéric Joliot, Orsay, France; ^4^Laboratory for Radiopharmacy, Department of Pharmaceutical and Pharmacological Sciences, KU Leuven, Leuven, Belgium

###### **Correspondence:** B. Janssen – Department of Radiology & Nuclear Medicine, Neuroscience Campus Amsterdam, VU University Medical Center, Amsterdam, The Netherlands

**Introduction:** Activated microglia are a hallmark of neuroinflammation (NI), which in turn is associated with neurodegenerative diseases. The P2Y_12_ receptor (P2Y_12_R) is upregulated in the anti-inflammatory phenotype of activated microglia, and is not expressed on macrophages and other brain cells. Therefore, P2Y_12_R could be an interesting new target for PET imaging of microglial activation in NI. Recently, a series of P2Y_12_R antagonists with high binding affinity was reported. Based on this series, the purpose of the present study was to label urea derivative **5** (IC_50_ = 6 nM) with carbon-11.

**Materials and methods:** The synthesis of sulfonylurea **5**, as reference, and azetidine amine **3** and sulfonyl azide **6**, as precursors for the radiosynthesis of [^11^C]**5**, is depicted below. Carbon-11-labelling was performed via a rhodium(I)-mediated [^11^C]CO carbonylation reaction [4,5]. [^11^C]**5** was evaluated using in vitro autoradiography of healthy mouse brains and ex vivo biodistribution and radiometabolite analyses in healthy male Wistar rats.

**Results:** Compound 5 and precursors 3 and 6 were successfully synthesised. [11C]5 was obtained in a radiochemical yield of 10±2 % (corrected for decay, calculated from [11C]CO2 (n=6)), and high (radio)chemical purity (≥98%) and specific activity (79±32 GBq·μmol-1 (n=6)). In in vitro autoradiography studies of the healthy mouse brain, [11C]5 could be blocked (81%) with ticagrelor, indicating specific binding to P2Y12R. However, rapid metabolism in rat plasma was observed with only 30±4% (n=3) of [11C]5 left at 45 min p.i.. In addition, ex vivo biodistribution revealed that [11C]5 did not enter the rat brain.

**Discussion/conclusion:** [^11^C]**5** is not suitable for in vivo studies, but can still be used in vitro to validate P2Y_12_R as a target for imaging the anti-inflammatory phenotype of activated microglia.

**Acknowledgement:** This research has received funding from the European Union's Seventh Framework Programme (FP7/2007-2013) under grant agreement n° HEALTH-F2-2011-278850 (INMiND).

### OP33 Radiosynthesis of a selective HDAC6 inhibitor [11C]KB631 and in vitro and ex vivo evaluation

#### Koen Vermeulen^1^, Muneer Ahamed^1^, Michael Schnekenburger^2^, Mathy Froeyen^3^, Dag Erlend Olberg^4^, Marc Diederich^2^, Guy Bormansa^1^

##### ^1^Lab of Radiopharmacy, KU Leuven, Leuven, Belgium; ^2^Laboratoire de Biologie Moléculaire et Cellulaire du Cancer, Luxembourg, Grand Duchy of Luxembourg; ^3^Laboratory for Medicinal Chemistry, Rega Institute of Medical Research, KU Leuven, Leuven, Belgium; ^4^School of Pharmacy, University of Oslo and Norwegian Medical Cyclotron Centre, Oslo, Norway

###### **Correspondence:** Koen Vermeulen – Lab of Radiopharmacy, KU Leuven, Leuven, Belgium

**Introduction**: HDAC6 has been reported as a regulator in numerous diseases ranging from different kinds of cancers (ovarian, breast, prostate,..) to neurological deficiencies (Alzheimer’s disease, Huntington’s disease, amyotrophic sclerosis,..) [1,2]. Still many characteristics and functions of HDAC6 in these pathologies are unrevealed. Hence, we aim to develop a selective HDAC6 PET tracer to visualize the dynamics of HDAC6 in normal and disease states. Recently, Lu and coworkers synthesized [^11^C]KB631, a highly potent (IC_50_=1.4 nM) and selective (3700-fold selectivity against HDAC1) PET tracer for HDAC6 imaging in the brain, which showed similar pharmacokinetic properties as tubastatin A [3]. However, [^11^C]KB631 showed low brain bioavailability [4]. In this regard we opted to use this tracer as a more peripheral imaging agent. The radiosynthesis was redesigned and preliminary results were obtained with a biodistribution study and autoradiography experiments in brain and tumor slices.

**Materials and methods:** [^11^C]KB631 was synthesized through methylation of the corresponding precursor (300 μg) with [^11^C]-MeI in anhydrous DMSO (250 μL) at 100 °C for 4 min. The biodistribution was studied in NMRI-mice at 2, 10, 30 and 60 min (n = 3/time point) post injection (p.i.). Organs and tissues were harvested and radioactivity was counted in a gamma-counter. Autoradiography studies were carried out with [^11^C]KB631 on wild-type Wistar rat brain tissue and PC3/LNCaP prostate tumor slices. Slices were incubated with 18.5 MBq/400μL (brain) or 18.5 MBq/250 μL (tumor) of tracer with/without 100 μM of cold authentic reference compound (KB631) or non-structural related pan-HDAC inhibitor Suberoylanilide hydroxamic acid (SAHA) [5].

**Results:** Based on prep HPLC integration, the methylation yield was 55 % with a radiochemical purity of 97 % and a specific activity of 48 GBq/μmol. Biodistribution studies indicated low brain uptake (<0.1 %ID at 2, 10, 30 and 60 min) and renal and hepatobiliary excretion. Autoradiography experiments showed regional binding. Binding in brain/tumor slices was highly displaceable in the presence of 100 μM non-labeled reference KB631 (up to 90% for brain, PC3 and LNCaP) or 100 μM SAHA (73% brain, 39% PC3 and 59% LNCaP).

**Discussion/conclusion:** We successfully radiolabeled and evaluated a potential carbon-11 labeled radiotracer for *in vitro* and *ex vivo* visualization of HDAC6. However, biodistribution studies indicated low brain uptake, peripheral potency still needs to be further examined. Autoradiography studies showed regional and displaceable binding. Furthermore, radiometabolite studies followed by μPET on a mice tumor model will be performed.

### OP34 Improving metabolic stability of fluorine-18 labelled verapamil analogues

#### Raaphorst RM^1^, Luurtsema G^2^, Lammertsma AA^1^, Elsinga PH^2^, Windhorst AD^1^

##### ^1^Department of Radiology & Nuclear Medicine, Neuroscience Campus Amsterdam, VU University Medical Center, Amsterdam, The Netherlands; ^2^Department of Nuclear Medicine and Molecular Imaging, University Medical Center Groningen, University of Groningen, Groningen, The Netherlands

###### **Correspondence:** Raaphorst RM – Department of Radiology & Nuclear Medicine, Neuroscience Campus Amsterdam, VU University Medical Center, Amsterdam, The Netherlands

**Introduction:** *(R)* **-**[^11^C]verapamil is widely used as a PET tracer for investigating P-glycoprotein (P-gp) function in patients with epilepsy, Alzheimer´s disease and other neurodegenerative diseases [1]. Recently, we have developed the fluorine-18 analogues *(R)*-*N*-[^18^F]fluoroethylverapamil (**1**) and *(R)*-*O*-[^18^F]fluoroethylnorverapamil (**2**), potentially enabling P-gp studies in centres without an on-site cyclotron. These analogues showed specific P-gp substrate behaviour, but metabolic stability was poor. The purpose of the present study was to assess whether deuterated analogues would have better metabolic stability.

**Materials and methods:** To a dried ^18^F/K_2.2.2_/K_2_CO_3_ complex, 2-bromoethyl-*d*_*4*_-tosylate in DMF was added and reacted for 10 min at 90°C to obtain 2-bromo-[^18^F]fluoroethane-*d*_*4*_. This was distilled at 100 °C through an AgOTf column at 200 °C into a cooled (0 °C) vial containing 1.5 mg of *(R)*-normethyl verapamil (**1a**) and 3 mg of K_2_CO_3_ in ACN. While stirring, this reaction mixture was heated at 120 °C for 15 min and purified by HPLC, resulting in **1b**. Tracer **2b**, **3b** and **4b** were obtained by direct fluorination of precursors **2a**, **3a** and **4a** and only **2b** required final Boc-deprotection with TFA. **1b** and **2b** were administered to Wistar rats, and the level of labelled metabolites was measured in blood plasma and brain samples.

**Results: 1b**, **2b**, **3b** and **4b** were obtained in a radiochemical yield of 3, 6, 5 and 10%, respectively, a purity >98% and a specific activity >80 GBq·μmol^-1^. Results of the metabolite analysis are presented. The deuterated analogues showed improved metabolic stability compared with the non-deuterated compounds.

**Discussion/conclusion:** Labelling of tracers **1b**-**4b** was successful. Although, both **1b** and **2b** showed significantly increased metabolic stability, total intact tracer levels at 15 min are still lower than desired. The resulting effect of increased metabolic stability for PET imaging will be evaluated in P-gp KO mice. To determine the effect of a deuterated *N*-methyl group, tracer **3b** and **4b** were designed and still need to be evaluated for metabolic stability.

### OP36 Development of a novel PET tracer for the activin receptor-like kinase 5

#### Lonneke Rotteveel^1^, Uta Funke^1^, Peter ten Dijke^3^, Harm Jan Bogaard^2^, Adriaan A. Lammertsma^1^, Albert D. Windhorst^1^

##### ^1^Department of Radiology & Nuclear medicine, Institute for Cardiovascular Research, VU University Medical Center, Amsterdam, The Netherlands; ^2^Department of Pulmonary Medicine, Institute for Cardiovascular Research, VU University Medical Center, Amsterdam, The Netherlands; ^3^Department of Molecular Cell Biology, Leiden University Medical Center, Leiden, The Netherlands

###### **Correspondence:** Lonneke Rotteveel – Department of Radiology & Nuclear medicine, Institute for Cardiovascular Research, VU University Medical Center, Amsterdam, The Netherlands

**Introduction:** Pulmonary arterial hypertension (PAH) is a disease in which pulmonary arterial obstruction increases vascular resistance, leading to right ventricular failure [1]. Inhibition of transforming growth factor-β (TGF-β) signalling via activin-receptor-like kinase 5 (ALK5) prevents progression and development of pulmonary hypertension [2]. To further understand the role of ALK5 in PAH, the purpose of this study was to synthesize a carbon-11 labeled ALK5 tracer (IC_50_ = 5.5 nM) [3] and to assess its potential as a positron emission tomography (PET) ligand for measuring ALK5 expression and activity *in vivo*.

**Materials and methods:** The [^11^C]ALK5 tracer was synthesized by a carboxylation reaction. The radiolabeling was carried out by heating [^11^C]CO_2_, the precursor molecule, isobutyl iodide and BEMP in DMSO for 10 minutes at 75°C. The tracer was evaluated using biodistribution and metabolite studies in Wistar rats (n=4 per time point at 15 and 60 min). In addition, specific binding was assessed using autoradiography on ALK5 expressing MDA-MB-231 tumor sections.

**Results:** The [^11^C]ALK5 tracer was synthesized with a yield of 18±6 %, a specific activity of 116±31 GBq·μmol^-1^ and a purity of > 95 %. The tracer showed a normal biodistribution *ex vivo* and a moderate stability *in vivo*. The autoradiograms presented binding of the tracer to the tumor sections. Pretreatment of the tumor sections with ALK5 blocking agents (EW-7197, SB-431542 and the ALK5 inhibitor) decreased the binding of the tracer significantly.

**Conclusion:** The ALK5 tracer was synthesized successfully, and initial *in vitro* and *ex vivo* studies indicate its potential as a putative tracer of ALK5, warranting further *in vivo* evaluation.

**Acknowledgment:** CVON is acknowledged for funding of this project and the BV Cyclotron VU for providing [^11^C]CO_2_.

### OP37 SPECT imaging and biodistribution studies of 111In-EGF-Au-PEG nanoparticles in vivo

#### Lei Song, Sarah Able, Nadia Falzone, Veerle Kersemans, Katherine Vallis

##### CR-UK/MRC Oxford Institute for Radiation Oncology, Department of Oncology, University of Oxford, Oxford, UK

###### **Correspondence:** Lei Song – CR-UK/MRC Oxford Institute for Radiation Oncology, Department of Oncology, University of Oxford, Oxford, UK

**Introduction:** Radiolabelled antibodies and peptides hold promise for molecular radiotherapy but are often limited by low payload resulting in delivery of inadequate amounts of radioactivity to tumour tissue and, therefore, modest therapeutic effect. We have developed PEGylated epidermal growth factor (EGF)-gold nanoparticles (NP) with a high indium-111 (^111^In) payload (^111^In-EGF-Au-PEG NPs) as a prototypic NP-based theranostic radiopharmaceutical.

**Materials and methods:** EGF-Au-PEG NPs were prepared via an interaction between gold and the disulphide bonds of EGF followed by PEGylation by mPEG-thiol (MW: 800, 2000, 6000) and characterised by SEC-HPLC, DLS and zeta potential. The targeting property of NPs with various PEG MWs was investigated by confocal imaging following exposure of MDA-MB-468 (1.3 x 10^6^ EGFR/cell) and MDA-MB-231/H2N (10^5^ EGFR/cell) cells to Cy3-EGF-Au-PEG NPs. ^111^In-EGF-Au-PEG (MW: 6000) and ^111^In-EGF-Au NPs were chosen for SPECT imaging and biodistribution studies using MDA-MB-468 xenograft-bearing mice.

**Results:** Successful PEGylation was confirmed by DLS and zeta potential measurements, showing the hydrodynamic sizes of NPs were 18.5, 19.4, 24.8 and 32.5 nm; the zeta potentials in water were -24, -15, -14 and -9 mV for EGF-Au and EGF-Au-PEG with MWs of 800, 2000 and 6000, respectively. SEC-HPLC showed that the retention time of EGF-Au-PEG NPs was shorter than EGF-Au NPs as PEGylation resulted in larger NPs. Confocal imaging demonstrated that both EGF-Au and EGF-Au-PEG NPs were efficiently bound and internalised by MDA-MB-468 cells. In vivo SPECT studies in mice bearing MDA-MB-468 xenografts revealed high tumour uptake in animals that received ^111^In-EGF-Au-PEG (MW: 6000) compared to ^111^In-EGF-Au. The tumour/muscle radioactivity ratios for ^111^In-EGF-Au-PEG and ^111^In-EGF-Au were 7.2 and 2.5.

**Conclusion:** An ^111^In-labelled EGF-Au-PEG nanosystem was developed. It enabled targeted delivery of a high ^111^In payload to an EGFR-positive cancer model that can be potentially exploited for molecularly targeted radiotherapy.

### OP38 Melanoma targeting with [99mTc(N)(PNP3)]-labeled NAPamide derivatives: preliminary pharmacological studies

#### Davide Carta^1^, Nicola Salvarese^2^, Wiebke Sihver^3^, Feng Gao^3^, Hans Jürgen Pietzsch^3^, Barbara Biondi^4^, Paolo Ruzza^4^, Fiorenzo Refosco^2^, Cristina Bolzati^2^

##### ^1^DSF, University of Padua, Via Marzolo 5, 35131 Padova, Italy; ^2^IENI-CNR, Corso Stati Uniti 4, 35127 Padova, Italy; ^3^Institute of Radiopharmaceutical Cancer Research, HZDR, Bautzner Landstrasse 400, 01328 Dresden, Germany; ^4^ICB-CNR, Via Marzolo 1, 35131 Padova, Italy

###### **Correspondence:** Cristina Bolzati **–** IENI-CNR, Corso Stati Uniti 4, 35127 Padova, Italy; ^3^Institute of Radiopharmaceutical Cancer Research, HZDR, Bautzner Landstrasse 400, 01328 Dresden, Germany

**Introduction:** Malignant melanoma is the most lethal form of skin cancer and the most commonly diagnosed malignancy among young adults with an increasing incidence. Hence, the development of new melanoma-specific pharmaceutical for diagnosis and/or therapy is a subject of great interest and intense research. The purpose of this study was to examine the effect of cyclization on the biological profile of [^99m^Tc(N)(PNP3)]-labeled α-MSH peptide analogs (PNP3 = N,N-bis(dimethoxypropylphosphinoethyl)methoxyethylamine).

**Method:** A lactam bridge-cyclized H-Cys-Ahx-βAla^3^-c[Lys^4^-Glu-His-D-Phe-Arg-Trp-Glu^10^]-Arg^11^-Pro-Val-NH_2_ (NAP―NS2) and the corresponding linear H-Cys-Ahx-βAla-Nle-Asp-His-D-Phe-Arg-Trp-Gly-NH_2_ (NAP-NS1) peptide were synthetized, characterized by ESI-MS spectroscopy and their MC1R binding affinity were determined in B16/F10 melanoma cells. In vitro stability and pharmacological parameters of [^99m^Tc(N)(NAP―NS1)(PNP3)]^+^ (**1**) and [^99m^Tc(N)(NAP―NS2)(PNP3)]^+^ (**2**) were assessed. Challenges with an excess of glutathione and cysteine and Log P values were also investigated. Furthermore, **1** and **2** were applied to study in vivo stability and the pharmacokinetic profiles on healthy rats.

**Results: 1** and **2** were obtained in high yield (RCY > 90%). Log P values demonstrate the hydrophilic nature of the radiolabelled peptides: -1.43 for **1**; - 2.087 for **2**. No significant variations in RCPs of both the complexes were observed in challenge experiments with an excess (10 mM) of glutathione and cysteine. A high in vitro stability was observed for both complexes after incubation in human and rat sera as well as in rat liver homogenate; a fast degradation of **2** was detected in kidneys homogenate. **1** retains a high receptor affinity (K_d_=7.1 ± 0.5 nM). Biodistribution data of **1** display a favorable pharmacokinetic profiles characterized by a fast blood clearance and elimination from normal tissues. A rapid excretion via the renal pathway was observed according to the high hydrophilic character of the radio-conjugate. The effect of the cyclization on the pharmacokinetic profile of **2** was reflected in a reduction of the blood clearance and of the elimination from the other organs, in particular, from excretory organs such as liver and kidneys.

**Conclusion:** Compared with the linear peptide, cyclization negatively affects the biological properties of NAP-NS2 peptide by reducing its binding affinity for MCR1R (Ki: 0.9±0.3 nM for NAP_NS1; 7.1±2.4 nM for NAP_NS2) and decreasing the overall excretion rate of the corresponding [^99m^Tc(N)(PNP3)]-labeled peptide from the body as well as its stability. Thus only the linear [^99m^Tc(N)(PNP3)-labeled peptide is suitable for further investigations in tumor bearing animals. This research was supported by MIUR through PRIN 20097FJHPZ-004 and FIRB “RINAME”2010-RBAP114AMK.

### OP39 [^68^Ga]NODAGA-RGD: cGMP synthesis and data from a phase I clinical study

#### Roland Haubner^1^, Armin Finkensted^2^, Armin Stegmair^1,3^, Christine Rangger^1^, Clemens Decristoforo^1^, Heinz Zoller^2^, Irene J. Virgolini^1^

##### ^1^Department of Nuclear Medicine, Medical University of Innsbruck, Innsbruck, Austria; ^2^Department of Internal Medicine, Medical University of Innsbruck, Austria; ^3^FH Gesundheit/University of Applied Sciences Tyrol, Innsbruck, Austria.

###### **Correspondence:** Roland Haubner –Department of Nuclear Medicine, Medical University of Innsbruck, Innsbruck, Austria

**Introduction:** Preclinical studies demonstrated that [^68^Ga]NODAGA-RGD allows imaging of integrin α_v_β_3_ expression using PET. Here we present all data (quality control, toxicological study, and dosimetry estimation) necessary to initialize clinical studies, the establishment of the remote controlled synthesis, and data from the phase I clinical study.

**Methods:** Labelling was carried out in a remote controlled synthesis unit under clean room conditions. Quality control included TLC, HPLC, GC, pH control, Ge-breakthrough, half-life, endotoxin content, and control of sterility. Storage stability after 4 h was studied and dose estimations based on animal data were carried out using OLINDA. Sprague-Dawley rats were used for an extended single dose toxicity study. The phase I clinical study included 9 patients with hepatocellular carcinoma (HCC). Static scans at 5, 30, and 60 min p.i. including 5 bed positions each were performed using the Discovery 690 PET/CT. Blood was sampled 30 and 60 min p.i. and urine 60 min p.i. and used for stability studies via HPLC.

**Results:** [^68^Ga]NODAGA-RGD could be produced in high radiochemical yield and radiochemical purity (HPLC and TLC >99%). The pH in the final isotonic saline formulation was approx. 6. Ethanol content was between 2.5 and 3.0% v/v. No detectable ^68^Ge-germanium was found. LAL test revealed 0.7 EU/ml. Sterility tests showed that all samples met the specifications according to Ph. Eur.. No radiolysis of the tracer was found in the formulated solution. The single dose toxicity study showed that the compound was well tolerated in animals. This was confirmed by the clinical study where no severe side effects were observed. High metabolic stability of [^68^Ga]NODAGA-RGD was found based on the analysis of blood and urine samples. The static scans showed rapid tracer elimination from the body with low background activity in almost all organs. The calculated effective dose was 21.5±5.4 μSv/MBq. Unfortunately, the investigated tumors did not show increased tracer accumulation indicating no or low integrin α_v_β_3_ expression.

**Conclusion:** This study revealed that [^68^Ga]NODAGA-RGD can be easily produced under GMP conditions and met the requirements for the clinical use. The phase I clinical study with patients bearing HCC did not allow identification of the lesions but demonstrated rapid elimination from the body, high metabolic stability and low radiation burden. The low tracer accumulation in the tumor might be related to low receptor expression, thus further studies are needed to verify the integrin α_v_β_3_ imaging properties.

### OP44 Implementation of a GMP-grade radiopharmacy facility in Maastricht

#### Ivo Pooters^1^, Maartje Lotz^1^, Roel Wierts^1^, Felix Mottaghy^1,2^, Matthias Bauwens^1,3^

##### ^1^Department of Radiology and Nuclear Medicine, MUMC+, Maastricht, The Netherlands; ^2^Nuclear Medicine, Uniklinikum Aachen, Aachen, Germany; ^3^Research School NUTRIM, Maastricht University, Maastricht, The Netherlands

###### **Correspondence:** Matthias Bauwens – Department of Radiology and Nuclear Medicine, MUMC+, Maastricht, The Netherlands

**Introduction:** In the Netherlands, a modified “light” version of the European GMP, i.e. GMP-z, for the (kit-) production of registered radiopharmaceuticals for individual patients in hospital pharmacies is in effect. However, GMP-z does not allow to synthesize radiopharmaceuticals for clinical trials or therapeutic applications, in which case the European GMP applies. At the MUMC, a growing interest in performing in-house clinical trials with new radiopharmaceuticals and in the synthesis of radiotherapeuticals led to the decision of upgrading the current Radiopharmacy facility. Several restrictions applied: radiopharmaceuticals will not be sold to third parties and the surface area of the entire facility is limited to 55 m^2^ (including the production lab, QC lab and separate sluices for personnel and goods). Additionally, the facility had to allow both preparation of routine ^99m^Tc-compounds as well as GMP-grade radiopharmaceuticals. This study examines the time line of implementation for such a small scale facility and may be relevant to other hospitals considering to comply with this regulation.

**Materials and methods:** A team was founded late 2012, including a radiochemist, QA officer, radiopharmacist, radiation safety officer, clinical physicist and a construction project leader (totaling nearly 5 fulltime-equivalents). User requirements for the facility, hotcells and laboratory equipment were determined. Preliminary building plans were drawn up and several expert companies chosen, specialized in HVAC, cleanroom construction and area and radiation monitoring.

**Results:** For the hotcells, a successful Factory Acceptance Test was performed in February 2015. Construction of the new facility started end of May 2015, starting with the planned installation of the hotcells for which removal of a section from the outer wall was necessary. The second phase of the reconstruction (setting up of a GMP-compliant cleanroom) is scheduled to start January 2016, leading to full operability in April 2016 and, upon inspection by the Dutch Inspectorate (IGZ), GMP-compliant production is expected by the end of 2016.

**Discussion/conclusion:** It is essential to have a high degree of in-house expertise when starting a radiopharmacy building project. Completely documented construction planning, based on a solid list of requirements, takes 2-3 years to build up and is a necessity to allow timely and high-quality construction.

### OP45 Setting up a GMP production of a new radiopharmaceutical

#### Forsback, Sarita^1^, Bergman Jörgen^1^, Kivelä Riikka^2^

##### ^1^Radiopharmaceutical Chemistry Laboratory, Turku PET Centre, University of Turku, Turku, Finland; ^2^Turku University Hospital, Hospital Pharmacy, Turku, Finland

###### **Correspondence:** Forsback, Sarita – Radiopharmaceutical Chemistry Laboratory, Turku PET Centre, University of Turku, Turku, Finland

**Introduction:** Good Manufacturing Practice (GMP) sets strict requirements for the manufacturing conditions, synthesis and quality of radiopharmaceuticals. Robust production and quality control methods for radiopharmaceuticals are essential for diverse and effective clinical utilization of Positron Emission Tomography (PET). This presentation describes the set up procedure of a new radiopharmaceutical in Turku PET Centre (TPC). All issues other than directly related to GMP such as toxicology, labeling chemistry, analytical methods or documentation for the authorities (i.e. IMPD) are not discussed here.

**Materials and Methods:** Quality standards and product specification are expressed in EU (Annex 15 Qualification and Validation, revision into operation 1 Oct 2015) and European Pharmacopoeia (8^th^ Edition). The setup of a new radiopharmaceutical at TPC requires existence of the following: Established quality system, Documentation system, Competent personnel, Classified clean rooms, Qualified equipment, System for material management. Initially the specifications for critical materials and primary packing materials of the new radiopharmaceutical must be defined. At TPC specifications for new radiopharmaceutical are: Appearance, Identification, Radioactivity, Radionuclidic identity, Radionuclidic purity, Radiochemical purity, Chemical purity, Residual solvents, Content of ethanol, pH, Sterility, Bacterial endotoxins, Shelf life.

**Results:** After ensuring that the production of a new radiopharmaceutical is robust and repeatable and all analytical methods including sterility and endotoxin tests are validated, the process can be validated according to a written validation plan. In addition bioburden (number of bacteria living on the drug solution before sterilization) must be determined. The aseptic processing of operators must also be confirmed by performing media fills (the performance of an aseptic manufacturing procedure using a sterile microbiological growth medium in place of the drug solution). At TPC the process validation includes three consecutive process validation batches which shall fulfill all specifications for the given radiopharmaceutical. This also qualifies the operator for production. Additional batches must be done in order to qualify more operators. Finally, process validation and documentation is compiled. Process validation report, method description for preparation and quality control and master batch record are written.

**Discussion/conclusion:** After all documentation has been accepted by QA, the new radiopharmaceutical is ready for clinical production and all changes to the process must be performed via change control process.

### OP48 In vitro and in vivo evaluation of 68-gallium labeled Fe3O4-DPD nanoparticles as potential PET/MRI imaging agents

#### M. Karageorgou^1^, M. Radović^2^, C. Tsoukalas^1^, B. Antic^2^, M. Gazouli^3^, M. Paravatou-Petsotas^1^, S. Xanthopouls^1^, M. Calamiotou^4^, D. Stamopoulos^4,5^, S. Vranješ-Durić^2^, P. Bouziotis^1^

##### ^1^Radiochemical Studies Laboratory, INRASTES, NCSR “Demokritos”, Athens, Greece; ^2^Vinča” Institute of Nuclear Sciences, Laboratory for Radioisotopes, University of Belgrade, Belgrade, Serbia; ^3^Department of Basic Medical Science, Laboratory of Biology, School of Medicine, NKUA, Athens, Greece; ^4^Department of Solid State Physics, NKUA, Athens, Greece; ^5^INN, NCSR “Demokritos”, Athens, Greece

###### **Correspondence:** M. Karageorgou – Radiochemical Studies Laboratory, INRASTES, NCSR “Demokritos”, Athens, Greece

**Introduction:** The combination of different imaging modalities has received increasing interest over the past decade, as it enables to overcome the limitations of a single imaging modality and ensures enhanced interpretation of diseases and abnormalities in vivo. Dual modality PET/MRI imaging agents, such as radiolabeled magnetic nanoparticles, are promising candidates for a number of diagnostic and therapeutic applications (i.e. MRI-magnetic hyperthermia and radiotherapy). The aim of the present study is to evaluate the efficacy of ^68^Ga labeled Fe_3_O_4_ superparamagnetic iron oxide nanoparticles (SPIONs) coated with DPD phosphonate, as potential PET/MRI imaging agents.

**Materials and methods:** SPIONs coated with biocompatible DPD-phosphonate were radiolabeled with positron-emitting Gallium-68 to quantify the accumulation of the nanoparticles *in vivo. In vitro* stability studies, in PBS, saline and human serum, were performed to evaluate their aqueous solubility *in vivo. In vivo* biodistribution study was performed in 9 healthy mice at 30, 60 and 120 min post-injection.

**Results:** ^68^Ga-Fe_3_O_4_-DPD SPIONs presented high radiolabeling yield (95%) and proved stable *in vitro*. The *in vivo* study exhibited significant liver and spleen uptake at all examined time points in healthy mice, whereas minor fractions attained in other organs. A small fraction of radiolabeled nanoparticles presented in bones is indicative of high affinity of phosphonate to bone tissue. The biodistribution profiles between ^68^Ga-Fe_3_O_4_-DPD SPIONs and free ^68^Ga-acetate were also compared, indicating different pharmacokinetic behavior for ^68^Ga-acetate, with no target tissue and excretion *via* the kidneys.

**Conclusion:** ^68^Ga-Fe_3_O_4_-DPD SPIONs demonstrated high radiolabeling efficiency and *in vitro* stability and satisfactory *in vivo* behavior. Cytotoxicity studies to explore the potential toxic effects of the nanoparticles, as well as biodistribution studies in tumor models, are in progress.

### OP49 Fast PET imaging of inflammation using 68Ga-citrate with Fe-containing salts of hydroxy acids

#### A. S. Lunev^1,2^, A. A. Larenkov^1^, K.A. Petrosova^1^, O. E. Klementyeva^1^, G. E. Kodina^1^

##### ^1^Burnasyan Federal Medical Biophysical Center of FMBA Russia, Moscow, Russia; ^2^Moscow State Academy of Veterinary Medicine and Biotechnology, Moscow, Russia

###### **Correspondence:** A. S. Lunev – Burnasyan Federal Medical Biophysical Center of FMBA Russia, Moscow, Russia

**Introduction:** ^68^Ga-citrate is one of the radiotracers for PET imaging of inflammation/infection. Gallium (like iron) has high affinity to blood transport proteins (e.g. transferrin) due to their similar physical/chemical properties [1]. One of the functions of transport proteins is delivery iron (gallium too) to inflammation/infection foci for inclusion in metabolic pathways related with eradication of pathology [2]. But excessive gallium bindings with proteins are cause of slow blood clearance, long accumulation time in foci (24-72 h) and exception of application possibility of the short-lived ^68^Ga (T½ = 1,13 h) unlike ^67^Ga (T½ = 78,26 h). Injection of additional nonradioactive chemical agents (e.g. Fe^3+^ containing salts of hydroxy acids: citrate, tartrate, lactate, etc.) competing with gallium to the protein joining (blocking of its metal binding capacity) is one of the ways to solve formulated problem. It can be used for correction of ^68^Ga-citrate pharmacokinetics for increasing of the blood clearance, accumulation in foci and fast imaging.

**Materials and methods.** ^68^Ga-citrate without/with extra injection of Fe^3+^ containing salts (citrate, tartrate, lactate, malate, and ascorbate) was injected mice with modeled lung infection (was got using intra lung injection of cell suspension of E. coli; acute phase of infection was evoluted for 3-4 days). PET/X-RAY Genisys4 (Sofie Bioscience, USA) was used for noninvasive PET imaging with subsequent reconstruction of imaging and their analysis (value of clearance, distribution volume). Scanning time is 10 min.

**Results:** I. v. injection of Fe^3+^ containing salts of hydroxy acids blocked the metal-binding capability of transferrin serum and others and allowed decreasing gallium-68 radioactivity in blood significantly and increasing accumulation in inflammation (3-5 time). It allowed receiving more informative PET-images of inflammation early (for 30-60 min after injection). Pharmacokinetic parameters proved it.

**Discussion/conclusion.** There was no statistically significant difference between ^68^Ga-citrate accumulation for different inflammation model because PET imaging is indication of pathological processes and isn't their identification.

## POSTER PRESENTATIONS

### PP01 Installation and validation of 11C-methionine synthesis

#### Kvernenes, O.H.^1^, Adamsen, T.C.H.^1,2^

##### ^1^Centre for Nuclear Medicine/PET, Department of Radiology, Haukeland University Hospital, Jonas Lies Vei 65, 5021 Bergen, Norway; ^2^Department of Chemistry, University of Bergen, Allégaten 41, 5007, Bergen, Norway

###### **Correspondence:** Kvernenes, O.H. – Centre for Nuclear Medicine/PET, Department of Radiology, Haukeland University Hospital, Jonas Lies Vei 65, 5021 Bergen, Norway

**Introduction:** In the installation of methionine as our latest tracer we chose the Eckert and Ziegler Modular-Lab PharmTracer as production module, utilizing the so called «wet-method» for producing methyl iodide. After complete installation in the hot cell, a simplified version of Ph.Eur (Ph Eur monograph 1617) release criterions was used, and the product was found to pass. HPLC showed excellent radio purity and the installation was completed.

**Materials and methods:** Homocysteine thiolactone, iodic acid, 0.1M lithium aluminum hydrid in THF, potassium dihydrogen phosphate were used in the C11-Met synthesis, Methods used are described in Ph.Eur, with the exception of chiral HPLC, in which an Astec Chirobiotic T column with 70:30:0.02, MeOH:H_2_O:HCOOH eluent was employed.

**Results:** During later analyses for residual solvents in connection with a GC-PQ, THF was found present in the product, 3-4 times above recommended level. The HPLC method in the Ph.Eur is 10 minutes, however, by increasing acquisition time, a radio peak appeared at about 18 minutes in the chromatogram. This peak was about 10-15% of total radio signal, meaning the required radio purity limit in Ph.Eur was not met. When analysing for enantiomeric purity, which was not initially done, about 12% of the *d*-enantiomer was found, not meeting the Ph.Eur limit. Increasing the flow of helium during the azeotropic distillation decreased residual solvent, and subsequently the ICH residual solvent limits was met. The radio peak in the HPLC was found to be methyl iodide (MeI) which is adsorbed and co-eluted from the C-18 Sep-Pak cartridges. Eckert and Ziegler proposed a rearrangement of the module tubing and a new synthesis template program enabling direct helium flushing of the C-18 Sep-Pak. By the use of this new configuration, removing excess MeI was achieved and the radio purity limits described by the Ph.Eur monograph was finally met. Different protocols are called for in the initial phase dissolving the precursor in ethanol and NaOH solution, however, tweaking these had little effect of the enantiomeric purity. Different batches of precursor was later analysed for enantiomeric purity. Large discrepancies were found between batches which directly influenced the enantiomeric ratio of the final product.

**Conclusion:** Great care should be taken in the installment of new synthesis systems, and all available tests should be run before system installation is approved. The Ph.Eur. methods may not always be sufficient to prove radio or chemical purity, even if a monograph exist.

### PP02 Fully automated synthesis of 68Ga-labelled peptides using the IBA Synthera® and Synthera® Extension modules

#### René Martin^1^, Sebastian Weidlich^1^, Anna-Maria Zerges^1^, Cristiana Gameiro^2^, Neva Lazarova^2^, Marco Müllera^2^

##### ^1^ABX GmbH, Radeberg, Germany; ^2^IBA RadioPharma Solutions, Louvain-La-Neuve, Belgium

###### **Correspondence:** René Martin – ABX GmbH, Radeberg, Germany

**Introduction:** The interest in ^68^Ga-tracers has been growing strongly over the last years, mainly due to new developments in prostate cancer imaging and therapy.^1,2^ In collaboration with IBA, we have established an automated synthesis of ^68^Ga-labelled peptides including ^68^Ga-DOTA-TATE/-NOC,^68^Ga-PMSA-11 and ^68^Ga-PSMA-617 on Synthera®.

**Materials and methods:** During the tests, an IBA Synthera® and the new Synthera® Extension module were used. ^68^Ga was obtained initially from an old iGG 100 generator in 5 mCi. The final tests were carried out in combination with a new GalliaPharm ^68^Ge/^68^Ga generator loaded with 50 mCi. A modified standard nucleophilic IFP^TM^ (IFP™ FDG) was designed for the synthesis process. Synthera® Extension was used for the elution of the generator. The eluate was loaded on a Chromafix PS-H^+^ cartridge and the hydrochloric acid waste - which contains most of the ^68^GeCl_4_ - was transferred into the waste container. The activity was eluted from the cation exchange cartridge using a solution of 5 M sodium chloride in 0.1 N hydrochloric acid^3^ and transferred directly into the reaction vessel which was pre-loaded with precursor in 1.5 M HEPES buffer. Labelling was carried out at 95 °C for 7 minutes. Purification was performed using a Sep-Pak® Light C18 cartridge. The product was eluted with a 1:1-mixture of ethanol/water and dispensed into the final vial through a sterile filter. Further dilution was performed with 0.9% saline by passing through the sterile filter. The peptides were synthesized in a GMP-compliant qualified area at ABX. For the DOTA-peptides, stock solutions were prepared (1 mg/ml) and freezed. For PSMA-11, vials with 10 μg of precursor were used.

**Results:** With 50 μg of DOTA-TATE, DOTA-NOC and PSMA-617, the final radiolabelled products were obtained in >60% uncorrected yield. For PSMA-11, only 10 μg of precursor were used. The radiochemical purity was >98% in all cases. The Ph. Eur. spot test for HEPES was performed and showed HEPES < 200 μg/V with V being 12 to 14 ml. The pH of the final solution was 5 to 5.5. Ethanol content was < 10%.

**Discussion/conclusion:** We have developed a dedicated disposable IFP cassette for the IBA Synthera® and Synthera® Extension modules, which delivers all common ^68^Ga-tracers in high yield. The use of dedicated single -use Gallium-68 IFP^TM^ allows for production of ^18^F-FDG on the same module with no cross-contamination.

### PP03 GMP compliant production of ^15^O-labeled water using IBA 18 MeV proton cyclotron

#### Gert Luurtsema^1^, Michèl de Vries^1^, Michel Ghyoot^2^, Gina van der Woude^1^, Rolf Zijlma^1^, Rudi Dierckx^1^, Hendrikus H. Boersma^1^, Philip H. Elsinga^1^

##### ^1^Department of Nuclear Medicine and Molecular Imaging, University Medical Center Groningen, University of Groningen, The Netherlands; ^2^IBA RadioPharma Solutions, Louvain-la-Neuve, Belgium

###### **Correspondence:** Gert Luurtsema – Department of Nuclear Medicine and Molecular Imaging, University Medical Center Groningen, University of Groningen, The Netherlands

**Introduction:** The most common tracer used for measuring brain perfusion is [^15^O]H_2_O. In general [^15^O] is produced using a cyclotron that accelerates deuterons onto a target filled with ^14^N_2_ with a trace of oxygen. Nowadays, cyclotrons have been developed that are only capable of accelerating protons. This abstract describes the performance of Cyclone 18 twin from IBA, which accelerate only protons, for production and administration of [^15^O]H_2_O.

**Methods:** The Cyclone 18 twin from IBA is a fixed energy cyclotron using 18 MeV protons in which H^-^ particles are accelerated and converted into protons (H^+^). Bombardment with protons of the special designed target filled to 25 bar takes place, initiating the nuclear reaction: ^15^N(p,n)^15^O. [^15^O]H_2_O was produced by conversion of the [^15^O]O_2_ using a new designed IBA chemistry module and placed in a shielded class A foam hood located in the GMP laboratory.During preparation, ^15^O gas flows from the cyclotron into the IBA chemistry module and is mixed with hydrogen gas and passed through a palladium column. The produced [^15^O]H_2_O is collected in a sterile 0.9% saline solution to obtain a final product suitable for patient administration. Validation of the production process included: (1) assessment of practical yields, (2) check on pharmaceutical requirements and (3) reproducibility of performance of both cyclotron and water module.

**Results:** According to our knowledge, this is the first time that GMP-production of ^15^O labeled water using IBA 18 MeV proton cyclotron is described. The method was validated and met all pharmacopeia specifications, and was subsequently approved for patient studies. The practical production yield of ^15^O labeled water using this method was ranged between 1300-1700 MBq measured in the syringe. This method is suitable for multiple batches within a time frame of 8 minutes. After validation more than 50 patient runs were performed with a reliability of > 99 %.

**Conclusion:** A new production method for ^15^O-labeled water using Cyclone 18 and dedicated chemistry module from IBA was described and qualified according to GMP regulations. It was demonstrated to be suitable for clinical use.

### PP04 In vitro Nuclear Imaging Potential of New Subphthalocyanine and Zinc Phthalocyanine

#### Fatma Yurt Lambrecht^1^, Ozge Er^1^, Mine Ince^2^, Cıgır Biray Avci^3^, Cumhur Gunduz^3^, Fatma Aslihan Sarı^4^

##### ^1^Department of Nuclear Applications, Institute of Nuclear Science, Ege University, Bornova, 35100, Izmir, Turkey; ^2^Department of Energy Systems Engineering, Faculty of Technology, Mersin University, TR-33480 Tarsus, Mersin, Turkey; ^3^Department of Medical Biology, Faculty of Medicine, Ege University, Bornova, 35100, Izmir, Turkey; ^4^Advanced Technology Research & Application Center, Mersin University, Ciftlikkoy Campus, TR-33343 Yenisehir, Mersin, Turkey

###### **Correspondence:** Fatma Yurt Lambrecht – Department of Nuclear Applications, Institute of Nuclear Science, Ege University, Bornova, 35100, Izmir, Turkey

**Introduction:** Cancer caused by abnormal cell proliferation has become a very common disease in recent years. Nuclear imaging has a significant place in cancer diagnosis. Photosensitizers (PS) such as chlorine, phthalocyanine, porphyrin and tetraprol derivatives are come up PDT applications. Furthermore, PSs accumulate selectively in the tumor tissue compared to normal tissue. Therefore, such compounds labeled with a radionuclide can be used as a probe for nuclear imaging. In present study, we synthesized a new Subphthalocyanine (SubPc) and Zinc phthalocyanine [Zn(II)Pc] and determined *in vitro* nuclear imaging potential.

**Materials and methods:** SubPc and Zn(II)Pc were synthesized according to published methods of Ince et al.The radiolabeling with ^131^I via iodogen method was carried out and optimization conditions (pH, amount of iodogen and reaction time) were determined. We will use EMT6/P (mouse mammary carcinoma), HeLa (human cervix adenocarcinoma) Hep G2 (human liver hepatocellular carcinoma), HT-29 (human colon colorectal adenocarcinoma), WI38 (human normal lung fibroblast) cell lines for the intracellular uptake studies. The intracellular uptake efficiency of ^131^I labeled Pcs are determined according to published study of Ocakoglu et al. in mentioned cell lines.

**Results:** We observed that the radiolabeling efficiencies of SubPc and Zn(II)Pc were significantly high (91.9±3.1% and 97.2±1.4%, respectively). The optimization conditions were determined as pH 9, 1 mg amount of iodogen and 30 minutes of reaction time for SubPc; pH 7, 1 mg amount of iodogen and 45 minutes of reaction time for Zn(II)Pc. *In vitro* cell line studies are in progress.

**Acknowledgement**: The authors gratefully acknowledge financial support by The Scientific and Technological Research Council of Turkey, TUBITAK (Grant no: 114Z430).

### PP05 Synthesis, Photodynamic Therapy Efficacy and Nuclear Imaging Potential of Zinc Phthalocyanines

#### Kasim Ocakoglu^1,2^, Ozge Er^3^, Onur Alp Ersoz^3^, Fatma Yurt Lambrecht^3^, Mine Ince^2^, Cagla Kayabasi^4^, Cumhur Gunduz^4^

##### ^1^Advanced Technology Research & Application Center, Mersin University, Ciftlikkoy Campus, TR-33343 Yenisehir, Mersin, Turkey; ^2^Department of Energy Systems Engineering, Faculty of Technology, Mersin University, TR-33480 Tarsus, Mersin, Turkey; ^3^Department of Nuclear Applications, Institute of Nuclear Science, Ege University, Bornova, 35100, Izmir, Turkey; ^4^Department of Medical Biology, Faculty of Medicine, Ege University, Bornova, 35100, Izmir, Turkey

###### **Correspondence:** Ozge Er – Department of Nuclear Applications, Institute of Nuclear Science, Ege University, Bornova, 35100, Izmir, Turkey

**Introduction:** In recent years, photodynamic therapy (PDT) gained speed in cancer treatment. This method is based on two steps. Firstly, a photosensitizer (PS) is accumulated in cancer tissue then flowed by light irradiation. During a photoreaction Type II, energy transfer occurs between the PS and molecular oxygen (O2), the exposed photosensitizers produce highly toxic singlet oxygen (1O2). The singlet oxygen is responsible for the cell damage.1-3 Pthalocyanines have high tumor uptake efficiencies. This property allows them to be used as bifunctional agents for PDT and nuclear imaging. In the present study, symmetrical Zn(II)Pc 1 and unsymmetrically substituted Zn(II)Pc 2 were synthesized and examined as a bifunctional agent for nuclear imaging and PDT.

**Materials and methods:** Zn(II)Pcs were synthesized according to a published procedure.4 The Zn(II)Pcs were radiolabeled with 131I by using iodogen method. Optimization conditions of radiolabeling (pH, amount of iodogen and reaction time) were determined. Biodistribution studies were performed with Albino Wistar female healthy rats. The radiolabeled Zn(II)Pcs were injected into the tail vein of each rats and then the rats were sacrificed at 30, 60 and 120 min post administration. Tissue samples were collected and counted using a Cd(Te)-RAD-501 single channel analyzer. In vitro the photodynamic therapy studies, EMT6/P (mouse mammary carcinoma), HeLa (human cervix adenocarcinoma) cell lines were used. Zn(II)Pc 1 and Zn(II)Pc 2 were exposed to red light (650 nm) at the doses of 10-30 J/cm2.

**Results:** The Zn(II)Pc 1 and Zn(II)Pc 2 were radiolabeled with 131I with high efficiency (93.4±1.6% and 91.4±1.6%, respectively). The results of biodistribution study showed that radiolabeled Zn(II)Pc 1 has high uptake on lung, large intestine, ovary and pancreas. However, the uptake of radiolabeled Zn(II)Pc 2 was determined as to be statically significant in pancreas and large intestine. Zn(II)Pc 1 was showed no phototoxic effect in both cell lines although PDT activity of Zn(II)Pc 2 in HeLa cell line was determined.

**Conclusion:** In conclusion the radiolabeled Zn(II)Pc 1 might be a promising imaging agent for lung, ovary pancreas, and colon tumors. However, the radiolabeled Zn(II)Pc 2 might be a promising nuclear imaging agent for colon and pancreas tumors, also promising PDT agent for cervical tumors.

**Acknowledgement:** The authors gratefully acknowledge financial support by The Scientific and Technological Research Council of Turkey, TUBITAK (Grant no: 112T565).

### PP06 Radio-U(H)PLC – the Search on the Optimal Flow Cell for the γ-Detector

#### Torsten Kniess, Sebastian Meister, Steffen Fischer, Jörg Steinbach

##### Helmholtz-Zentrum Dresden-Rossendorf, Institute of Radiopharmaceutical Cancer Research, 01314 Dresden, Germany

###### **Correspondence:** Torsten Kniess – Helmholtz-Zentrum Dresden-Rossendorf, Institute of Radiopharmaceutical Cancer Research, 01314 Dresden, Germany

**Introduction:** Radio-HPLC is routinely used for analysis and quality control of radiopharmaceuticals. The introduction of U(H)PLC enabled decreased detection levels, improved resolution and significant shorter analysis times; crucial parameters for radiotracer analysis. Despite these benefits only a few examples of radio-U(H)PLC have been reported; one reason may be the absence of a suitable γ-detector with a flow cell which is compatible to UPLC conditions. We used a commercial γ-detector in combination with different flow cell inserts and report on their effect on the sensitivity and resolution of the γ-signal.

**Materials and methods:** An ACQUITY UPLC H-Class System (Waters) was used with a GABI Star γ-detector (Raytest) equipped with a 2”x 2” pinhole scintillation crystal in combination with three different flow cells. UPLC was performed on an ACQUITY HSS C18 column (2.1 x 100 mm, 1,8 μm) with a flow rate of 0.65 mL/min and a 3 min gradient of 30/70 acetonitrile/0.1% TFA. The sigma-1 receptor imaging probe [^18^F]fluspidine served as analyte with 2 μL injection volume, containing 8 – 400 kBq of [^18^F]fluspidine.

**Results:** For best resolution and avoiding an excessive back-pressure to the PDA cell (<70 bar) the capillary between PDA and γ-detector should be as short as possible. This was realized by placing the γ-detector in 30 cm distance to the PDA-detector on a special table. Three different flow cells were applied:

a) *Raytest standard flow cell* (5 μL),

b) *Raytest 2” capillary holder* equipped with a UPLC capillary 0.004” ID (9/5/2 nL),

c) *self-build lead insert* with a 5 mm hole and UPLC capillary (2 nL).

By injecting 400 kBq of [^18^F]fluspidine and using the different flow cells, γ-signals having a peak width between 0.13 and 0.20 mm and a peak intensity 750 - 4400 cps were obtained. The capillary holder (5 nL) and the self-build insert (2 nL) showed a comparable peak width, but differentiated sensitivity. To determine the minimum detectable level samples between 170 kBq and 8 kBq of [^18^F]fluspidine were injected; with 15 kBq of [^18^F]fluspidine a good signal to background ratio of 6:1 was achieved. **Conclusion:** By performing radio-U(HPLC) with a GABI Star γ-detector equipped with a *Raytest 2” capillary holder* (5 nL), γ-signals with good sensitivity and peak width can be obtained. In addition, the self-build flow cell insert (2 nL) gave the highest sensitivity and the best signal to background ratio.

### PP07 Radiolabeling, characterization & biodistribution study of cysteine and its derivatives with Tc99m

#### Rabia Ashfaq^1,2^, Saeed Iqbal^2^, Atiq-ur-Rehman^1^, Irfan ullah Khan^3^

##### ^1^Nuclear Medicine Department, Shaukat Khanum Cancer Hospital and Research Centre, Lahore, Pakistan; ^2^Forman Christian College, Lahore, Pakistan; ^3^Institute of Nuclear medicine and Oncology, Lahore, Pakistan

###### **Correspondence:** Rabia Ashfaq – Nuclear Medicine Department, Shaukat Khanum Cancer Hospital and Research Centre, Lahore, Pakistan

**Introduction:** Several studies are available, which highlight the use of Schiff based as ligands for radio-labeling, but very little work has been reported with Marcepto compounds. It is desirable to include such compounds in the design of radiopharmaceuticals due to their importance in the biological system.

**Materials and methods:** Equimolar L – cysteine and salicylaldehyde in distilled water and ethanol were heated to form a white colored ligand. The product formed was analyzed using FT – IR, thermo gravimetric analysis and elemental analysis. Radiolabeling of the ligand was performed using ^99m^Tc from ^99^Mo generator. SnCl_2_.H_2_O was used as a reducing agent. Radio TLC was performed using SG plates, 0.9% NaCl and acetone as the mobile phases. Geiger Muller counter detected the counts on the SG plates. After quality control the radiolabeled drug was injected IV into the animal ear. Scanning was performed under the gamma camera.

**Results:** Ligand synthesis was verified from IR indicating the presence of the closed ring structure Thiozolidine ring ^1^. IR showed absence of chromophore group hence the ligand was colorless. DSC peaks indicated the reaction type as endothermic. Ligand appeared to be stable in DMSO. Effective radiolabeling was achieved using lyophilized tin chloride pyrophosphate cold kit in NaCl (0.9%). Optimization of ph, temperature and radioactivity was done using DOE. Maximum radiolabeling was achieved at pH 5.Animal study was performed for bio distribution of the radiopharmaceutical. ^99m^Tc – ligand uptake was seen in the soft organs immediately after injection. The areas showing higher radiotracer uptake were kidney (20 %), liver (35 %), bones (15%) and brain (10 %). Delayed images showed the radiotracer retention in the soft organs as well as in the spine of the animals. Comparative study was performed using ^99m^Tc – MDP and ^99m^Tc PHYTATE.

**Conclusion:** Salicylaldicysteine is efficiently labeled with ^99m^Tc and is suitable in simultaneous scanning of liver, kidney and brain. Due to uptake in all soft organs in dynamic flow followed with delay images, radiotracer retention in the brain is worth consideration. This indicates the ability of drug in crossing the blood brain barrier. This study also highlights the importance of sulfur containing ligands in brain scanning

### PP08 Radiolabelling of poly (lactic-co.glycolic acid) (PLGA) nanoparticles with 99mTC

#### R Iglesias-Jerez^1^, Cayero-Otero^2^, L. Martín-Banderas^2^, A. Perera-Pintado^3^, I. Borrego-Dorado^1^

##### ^1^Servicio de Medicina Nuclear, Hospital Universitario Virgen del Rocío, Avda Manuel Siurot s/n. 41013, Seville, Spain; ^2^Dpt. Farmacia y Tecnología Farmacéutica, Facultad de Farmacia, Universidad de Sevilla. c/Prof. Gracía González n°2, 41012, Seville, Spain; ^3^Dirección de Investigaciones Clínicas, Centro de Isotopos, 34 No. 4501 e/45 y 47, Kohly, Playa, La Habana, CP 11300, Cuba

###### **Correspondence:** R Iglesias-Jerez – Servicio de Medicina Nuclear, Hospital Universitario Virgen del Rocío, Avda Manuel Siurot s/n. 41013, Seville, Spain

**Introduction:** Radiolabelled nanoparticles have gained a widespread application in the diagnosis and therapy of several diseases (inflammation/infection, cancer, and others). PLGA Nanoparticles (NPs) prepared by solvent emulsion evaporation method [1] have shown more suitable characteristics with regard to those produced by nanoprecipitation: better size, a higher homogeneity and higher encapsulation efficiency. The combination of ^99m^ Tc + PLGA nanoparticles (platforms with excellent biodegradability and biocompatibility) could allow obtaining a suitable nanosystem for theranosis. The aim of the present work is to optimize the radiolabelling of PLGA nanoparticles with ^99m^Tc.

**Materials and methods:** PLGA nanoparticles were prepared using the nanoprecipitation method [1]. Then, 5 mg of lyophilized NPs were dispersed in 1 mL 0.9% NaCl and stored in a vacuum vials. Different amounts of stannous chloride dehydrate in aqueous solution (30, 20, 4, 2, 1 and 0,1 μg) were added. Then, was added ≈74 MBq (2 mCi) of ^99m^Tc in 1 mL of 0.9% NaCl. Finally, the suspension was incubated for 10 min. ^99m^Tc -NPs suspensions were analyzed by thin layer chromatography (TLC) with silica gel strips (10 x2,5 cm). With 0.9% NaCl as the mobile phase, free pertechnetate ran with the front, meanwhile particles stayed in the start. Using a solution of pyridine: acetic acid: water (3: 5: 15), radiocolloids remained at start, NPs migrated with Rf = 0.3 and free pertechnetate moved with Rf = 0.7-0.8 [2].

**Results:** Using 1 μg of SnCl_2_*2H_2_O as a reducing agent, PLGA nanoparticles (without surface modification) were labeled with ^99m^ Tc with a yield ≥ 90 %.PLGA nanoparticles size ranged from 160-180 nm in diameter with an electric surface of -36 mV. After radiolabelling, particles increased in size up to a diameter ≈ 380 nm.

**Conclusions:** Results obtained, indicate that the procedures of nanoparticle synthesis, radiolabelling and quality control were reproducible and right to design a biodegradable nanosystem suitable for *in vivo* theranosis.

### PP09 Development of [^18^F]PD-410 as a non-peptidic PET radiotracer for gastrin releasing peptide receptors

#### Ines Farinha-Antunes^2^, Chantal Kwizera^2^, Enza Lacivita^1^, Ermelinda Lucente^1^, Mauro Niso^1^, Paola De Giorgio^1^, Roberto Perrone^1,3^, Nicola A. Colabufo^1,3^, Philip H. Elsinga^2^, Marcello Leopoldo^1,3^

##### ^1^Dipartimento di Farmacia – Scienze del Farmaco, Università degli Studi di Bari “Aldo Moro”, Bari, Italy; ^2^Department of Nuclear Medicine and Molecular Imaging, University Medical Center Groningen, University of Groningen, Groningen, The Netherlands; ^3^BIOFORDRUG s.r.l., Spin-off, Università degli Studi di Bari “Aldo Moro”, Bari, Italy

###### **Correspondence:** Philip H. Elsinga – Department of Nuclear Medicine and Molecular Imaging, University Medical Center Groningen, University of Groningen, Groningen, The Netherlands

**Introduction:** The Gastrin Releasing Peptide (GRP) receptor has high expression on prostate cancer cells. Consequently, bombesin, a 14-amino acid peptide that binds with high affinity to GRP receptors, has been widely studied for the development of radiopeptides for application in prostate cancer imaging. To overcome some limitations of radiolabelled peptides, we developed a small molecule non-peptidic radiotracer targeting GRP receptors. Small molecules offer various advantages over peptides since they can be suitably designed to modulate potency, selectivity, lipophilicity, and cell permeability and do not suffer from poor tissue penetration, poor serum stability, and quick elimination. The aim of the present study is to develop a non-peptidic fluorine-18 PET radioligand with antagonist properties, for visualization of GRP receptors. PD-410 ((*S*)-3-(1*H-*indol-3-yl)*-N-*[1-[5-(2-fluoroethoxy)pyridin-2-yl]cyclohexylmethyl]-2-methyl-2-[3-(4-nitrophenyl)ureido]propionamide) was selected as potent compound for ^18^F-radiolabelling (K_i_ was 38 ± 2 nM).

**Methods:** [^18^F]PD410 was synthesized by reaction of [^18^F]fluoroethyl tosylate with the corresponding phenol precursor in presence of NaH followed by HPLC-purification. Cell experiments were carried out with PC3 cells to determine cell uptake, efflux and *in vitro* binding affinity.

**Results:** In contrast to other [^18^F]fluoroethylation reactions, RCY was low (<5%) because of substantial defluorination. The uptake of [^18^F]PD410 in PC3 prostate cancer cells gradually increased within 60 minutes of incubation, thereafter reaching a plateau. The maximum cellular associated radioactivity was found to be 70%. Non-specific binding accounted for 40%. The efflux kinetics of [^18^F]PD410 showed rapid dissociation of the tracer, remaining only 6% of the tracer within 120 min. This radioactivity decreased exponentially with a half-life of 13 minutes. *In vitro* binding affinity of [^18^F]PD410 was plotted as sigmoid curves for the displacement of [^18^F]PD410 as a function of increasing concentrations of the GRP receptor specific inhibitor Glu[Aca-BN(7-14)]_2_. The IC_50_ value was 0.10 nM for Glu[Aca-BN(7-14)].

**Conclusions:** [18F]PD410 displayed high affinity for GRP receptor and the in vitro results warrant further in vivo PET-studies.

### PP10 An improved nucleophilic synthesis of 2-(3,4-dimethoxyphenyl)-6-(2-[18F]fluoroethoxy) benzothiazole ([18F]FEDMBT), potential diagnostic agent for breast cancer imaging by PET

#### V.V. Vaulina^1^, O.S. Fedorova^1^, V.V. Orlovskaja^1^, С.L. Chen^2^, G.Y. Li^2^, F.C. Meng^2^, R.S. Liu^2,3^, H.E. Wang^2^, R.N. Krasikova^1^

##### ^1^N.P. Bechtereva Institute of Human Brain, Russian Academy of Science, Saint-Petersburg, Russian Federation; ^2^Department of Biomedical Imaging and Radiological Sciences, National Yang-Ming University Taipei, Taiwan; ^3^National PET/Cyclotron Center, Veterans General Hospital, Taipei, Taiwan

###### **Correspondence:** O.S. Fedorova – N.P. Bechtereva Institute of Human Brain, Russian Academy of Science, Saint-Petersburg, Russian Federation

**Introduction:** Substituted benzotiazoles (BT) are a class of compounds with high affinity and selectivity to aryl hydrocarbon receptor (AhR), a receptor often expressed in breast cancer tissue. Among them 2-(3,4-dimethoxyphenyl)-5-fluorobenzothiazole (PMX 610) exhibited very potent (GI_50_ < 0.1 nM) antitumor properties *in vitro* in human breast cancer cell lines. Based on that scaffold we recently suggested ^18^F-fluoroethylated analogue of PMX 610, the 2-(3,4-dimethoxyphenyl)-6-(2-[^18^F]fluoroethoxy)benzothiazole ([^18^F]FEDMBT)^1^. Preliminary studies showed high accumulation of the radiotracer in MCF-7, MDA-MB468 and MDA-MB231 breast cancer cells. Here we report an improved synthesis and purification of [^18^F]FEDMBT for preclinical trials.

**Materials and methods:** The extent of ^18^F-fluorination of labeling precursor was followed via radioTLC (EtAc:hexane 1:2). The HPLC purification was performed using Waters C18 XBridge 250 x 10 mm column, 0.05M NH_4_OAc:EtOH, 50:50, 3 ml/min, UV 254 nm. For SPE purification 1.2 mL of aqueous 25% EtOH was added to the reaction mixture (5 mg of precursor, 0.6 mL of DMF) to prevent precipitate formation. The resulting solution was passed through the Sep-Pak tC18 Plus Long cartridge. The cartridge was then rinsed with 2 mL of 50% aq. EtOH allowing for complete removal of the precursor. The product, [^18^F]FEDMBT, was recovered by successive elution of the cartridge with 2 and 1 mL of 55% aq. EtOH.

**Results** The [^18^F]FEDMBT was prepared via direct nucleophilic fluorination of the corresponding tosyl precursor^1^. Under optimal reaction conditions (kryptofix 2.2.2., DMF, 140°C, 5 min) a high ^18^F-incorporation rate of 80-85% was achieved. The HPLC purification afforded the product in >99% radiochemical purity and RCY of 55% (decay corrected). Isolated product fraction was mixed with phosphate buffered saline to adjust the pH and ethanol concentration, without final re-formulation step. Fractionated SPE purification afforded product with >99% RCP and 60% RCY (decay corrected). While SPE technique allowed for complete removal of precursor, a minor chemical impurity (not identified) remained in the final formulation. Work is being undertaken now to resolve this issue.

**Discussion/conclusion:** An optimized synthesis of [^18^F]FEDMBT afforded product with radiochemical purity and high yield. It can be easy automated using TracerLab FX-N Pro platform. The suggested “HPLC free” SPE purification can be applied in the production of [^18^F]FEDMBT for ongoing *in vitro* cell lines experiments and preclinical trials. This study was supported by RFBR grant 15-54-52026/15 and MOST 104-2923-B-010-001-MY2.

### PP11 Internal radiation dose assessment of radiopharmaceuticals prepared with accelerator-produced 99mTc

#### Laura Meléndez-Alafort^1^, Mohamed Abozeid^1^, Guillermina Ferro-Flores^2^, Anna Negri^3^, Michele Bello^4^, Nikolay Uzunov^5,6^, Martha Paiusco^3^, Juan Esposito^6^, Antonio Rosato^1,3^

##### ^1^DiSCOG-University of Padua, Padua, Italy; ^2^ININ, Ocoyoacac, México; ^3^IOV Padua, Padua, Italy; ^4^DFA-University of Padua, Padua, Italy; ^5^Faculty of Natural Sciences, University of Shumen, Shumen, Bulgaria; ^6^INFN-LNL, Legnaro, Italy

###### **Correspondence:** Laura Meléndez-Alafort – DiSCOG-University of Padua, Padua, Italy

**Introduction**: 99mTc is the radionuclide most widely used in diagnostic nuclear medicine. It is available from 99Mo/99mTc generators, where 99Mo is obtained by a fission reaction in nuclear reactors. Direct reactions using proton beams [e.g. 100Mo(p,2n)99mTc] are a reliable and relatively cost-effective method to fulfill the shortage of this isotope given the imminent closure of the existing old reactors. However, the results of LARAMED project from the LNL-INFN showed that the extracted solution of 99mTc from the proton-bombarded 100Mo-enriched target contains small quantities of several technetium radioisotopes (93Tc, 94Tc, 95Tc, 95mTc and 96Tc)[1]. The aim of this work was to estimate the total contribution of technetium radioisotopes to the patient radiation absorbed dose after administration of four radiopharmaceuticals prepared with technetium-99m obtained from the 100Mo(p,2n)99mTc reaction.

**Methods:** The internal radiation absorbed doses of pertechnetate, sestamibi, hexamethylpropyleneamine oxime (HMPAO), and disodium etidronate (HEDP) radiopharmaceuticals, were assessed considering both technetium-99m prepared from the 100Mo(p,2n)99mTc reaction as well as from generators. Time-integrated activity in the main source organs [Ã(rs,TD)] for each radioisotope was calculated using the radiopharmaceutical biokinetic models published by the International Commission on Radiological Protection (Publication 53 and 80, ICRP). OLINDA/EXM 1.1 software was applied for dose calculations using an adult male phantom as a program input and the above Ã(rs,TD) calculated values. The total absorbed dose in the target organs, produced by the mixture of technetium radioisotopes, was calculated for 3 time periods after 100Mo irradiation.

**Results:** The amounts of other technetium radioisotopes in the 99mTc-radiopharmaceuticals produced a low increase of radiation doses. Bone marrow is the organ that exhibited the highest difference between the dose obtained from the generator-produced 99mTc and the accelerator-produced one. For example, HEDP bone marrow dose (4.41E-03 mSv/MBq) showed an increase in the of 8.5% (4.79E-03 mSv/MBq), 11.8% (4.93E-03 mSv/MBq) and 17.7% (5.19E-03 mSv/MBq) using 99mTc obtained at 9, 14, and 19 h after 100Mo irradiation, respectively. The corresponding differences for sestamibi, pertechnetate and HMPAO were less than 8%, 10% and 14% for the same irradiation times.

**Conclusion**: The increase of radiation doses caused by 93Tc, 94Tc, 95Tc, 95mTc and 96Tc, compared with the dose from 99mTc is relatively low. However, its impact should not be underestimated. Administering the labeled radiopharmaceuticals at least nine hours after the target irradiation is advisable to reduce the irradiation caused by the other technetium radioisotopes.

### PP12 A specialized five-compartmental model software for pharmacokinetic parameters calculation

#### Laura Meléndez-Alafort^1^, Cristina Bolzati^2^, Guillermina Ferro-Flores^3^, Nicola Salvarese^1^, Debora Carpanese^1^, Mohamed Abozeid^1^, Antonio Rosato^1,4^, Nikolay Uzunov^5^

##### ^1^DiSCOG-University of Padua, Padua, Italy; ^2^IENI-CNR, Padua, Italy; ^3^ININ, Ocoyoacac, México; ^4^IOV Padua, Padua, Italy; ^5^Faculty of Natural Sciences, University of Shumen, Shumen, Bulgaria

###### **Correspondence:** Laura Meléndez-Alafort – DiSCOG-University of Padua, Padua, Italy

**Introduction:** The use of pharmacokinetics modeling in preclinical nuclear medicine is very limited although it is well known that such models could be valuable tools to study radiopharmaceutical-properties1. Nowadays, the preclinical evaluation of new radiopharmaceuticals is still focused only on biodistribution studies, mainly because of the lack of currently available user-friendly specialized programs to assist researchers in calculating radiopharmacokinetic parameters.

**Methods:** A dedicated spreadsheet software to calculate pharmacokinetic parameters such as tracer-biological half-life (T1/2), mean residence time (MRT) for each compartment, as well as the fraction of the tracer leaving the compartments per unit time (transfer rate constant, TRC), has been developed. The menu-driven spreadsheet software is based on five-Compartment Kinetic Model (CoKiMo). CoKiMo was used to study pharmacokinetic parameters of 11 99mTc-nitrido complexes, reported as potential myocardial perfusion-imaging agents (MPIAs), of general formula [99mTcN(DTC-Ln)(PNP)]+ (DTC-Ln= alicyclic dithiocarbamates; PNP= diphosphinoamine)2, and the results were compared with those of 99mTc-Sestamibi and 99mTc-Tetrofosmin. Biodistribution studies for all MPIAs have been performed in Sprague-Dawley rats (n = 312) to determine organ uptake.

**Results:** MPIAs time-activity curves have been obtained using CoKiMo for 5 compartments, by interpolation of organ activity values measured at different time points. Curves have been integrated to calculate T1/2 and MRT in the studied organs of all MPIAs. Subsequently software employs a module with implemented optimization procedure, dedicated to calculate the values of the TRCs. Finally, CoKiMo calculations were validated by comparison with two different software: Olinda/Exm and SAAM II. [99mTcN-(PNP)]+-complexes showed a faster blood and liver clearance as compared to 99mTc-Sestamibi and 99mTc-Tetrofosmin, in agreement with some previous reported studies2. However, it was found that when CoKiMo pharmacokinetic data were used, a wide quantitative comparison between all MPIAs was possible, which in fact, was not possible using only the biodistribution data. The analysis of the results showed that 99mTcN-PDTC3 turned out to be the best candidate for translation into clinical practice due to its rapid and high heart uptake, and fast liver and lung clearance, which enabled an early visualization of myocardial tissue.

**Conclusion**: CoKiMo proved to be an easy to use and flexible specialized software to study the kinetic of a great number of pharmaceuticals labeled with an unlimited number of radionuclides. Therefore, it can be a useful tool for an extensive preclinical characterization of new radiopharmaceuticals.

### PP13 Molecular imaging of the pharmacokinetic behavior of low molecular weight ^18^F-labeled PEtOx in comparison to ^89^Zr-labeled PEtOx

#### Palmieri L^1,2^, Verbrugghen T^1^, Glassner M^3^, Hoogenboom R^2^, Staelens S^1^, Wyffels L^1,2^

##### ^1^Molecular Imaging Center Antwerp, University of Antwerp, Antwerp, Belgium; ^2^Department of Nuclear Medicine, Antwerp University Hospital, Antwerp, Belgium; ^3^Supramolecular Chemistry, Ghent University, Ghent, Belgium

###### **Correspondence:** L Palmieri – Molecular Imaging Center Antwerp, University of Antwerp, Antwerp, Belgium

**Introduction:** PEGylation is a methodology that is commonly used to improve the PK profile of radiotracers. In recent years biocompatible poly(2-alkyl-2-oxazoline)s (PAOx) gained a lot of attention as alternative to PEG because of their much higher chemical versatility. PAOx can be equipped with side-chain functionalities, allowing loading multiple targeting molecules, while the chain-end can be selectively radiolabeled. We previously evaluated the PK behavior of ^89^Zr-labeled poly(2-ethyl-2-oxazoline)s (^89^Zr-PEtOx) in a molecular weight (MW) range of 5-110kDa using μPET imaging. While we found the expected increase of circulation time with increasing MW, the 5kDa [^89^Zr]-PEtOx demonstrated unusual high kidney retention. We now developed [^18^F]-PEtOx 5kDa and compared its pharmacokinetic behavior to [^89^Zr]-PEtOx 5kDa.

**Materials and methods:** PEtOx was radiolabeled with ^18^F using SPAAC. For this, PEtOx was first derivatized with bicyclononyne (BCN). Automated ^18^F-labeling of BCN-PEtOx was performed by reaction of azidoethyl tosylate with azeotropically dried ^18^F^-^ followed by distillation of the formed [^18^F]-fluorethylazide into a second reactor for reaction with BCN-POX. The reaction mixture was purified using a PD-10 cartridge and QC was performed using a Zenix-C 80Å column. To evaluate the PK profile, C57BL/6 mice (n=4) were intravenously injected with [^18^F]-BCN-PEtOx (16.47±1.68Mbq, ~100μg) and dynamically scanned over the first 2h. Volumes of interest were delineated for heart, liver and kidneys to calculate standard uptake values (SUV).

**Results**: [^18^F]-BCN-PEtOx was obtained with a RCY of 4.30% (n=4, decay corrected to EOB) and a RCP of 100% after PD-10 purification. As was previously demonstrated for [^89^Zr]-PEtOx 5kDa, μPET imaging revealed rapid and complete blood clearance of [^18^F]-PEtOx 5kDa (SUV=2.42±0.16 at 10min pi and SUV=0.27±0.08 at 1h pi). Peak liver uptake related to perfusion was reached within 1min pi (SUV=2.92±0.18) and quickly decreased (SUV=0.50±0.03 at 10min pi and SUV=0.07±0.00 at 1h pi) indicating only minor contribution of the liver in the clearance of [^18^F]-PEtOx 5kDa. The kidneys displayed a high initial uptake (SUV=5.67±0.87 at 3.75min pi versus 7.58±2.54 at 3.75min pi for [^89^Zr]-Df-PEtOx 5kDa) but where [^89^Zr]-Df-PEtOx 5kDa was not cleared from the kidneys (SUV=2.36±0.84 at 24h pi), [^18^F]-PEtOx 5kDa showed a fast and almost complete clearance (SUV=0.27±0.08 at 1h pi and SUV=0.12±0.05 at 2h pi).

**Conlusions**: [^18^F]-PEtOx 5kDa is quickly cleared from the body and does not display high kidney retention. The unusual kidney retention of [^89^Zr]-Df-PEtOx 5kDa is therefore most likely related to endocytosis and lysosomal degradation of the radiolabeled polymer followed by trapping of radiometal ^89^Zr in the proximal tubules.

### PP14 Towards nucleophilic synthesis of the α-[18F]fluoropropyl-L-dihydroxyphenylalanine

#### V. V. Orlovskaja^1^, O. F. Kuznetsova^1^, O. S. Fedorova^1^, V. I. Maleev^2^; Yu. N. Belokon^2^; A. Geolchanyan^3^; A. S. Saghyan^3^; L. Mu^4^, R. Schibli^4^; S. M. Ametamey^4^; R. N. Krasikova^1^

##### ^1^N.P. Bechtereva Institute of Human Brain, RAS, St.-Petersburg, Russia; ^2^A.N. Nesmeyanov Institute of Organoelement Compounds, RAS, Moscow, Russia; ^3^SPC Armbiotechnology NAS, Yerevan, Republic of Armenia; ^4^Center for Radiopharmaceutical Sciences of ETH, PSI and USZ, Zurich, Switzerland

###### **Correspondence:** V. V. Orlovskaja – N.P. Bechtereva Institute of Human Brain, RAS, St.-Petersburg, Russia

**Introduction:** The interest towards use of 6-[18F]fluoro-L-DOPA as a PET radiotracer for the evaluation of dopaminergic system’s state and also as tumor diagnostic agent has been steadily increasing during the last decade. Considerable work is being undertaken to overcome the practical difficulties encountered in the production of 6-[18F]fluoro-L-DOPA and other ring-fluorinated amino acids via nucleophilic substitution route. Aromatic amino acids carrying monofluoro methyl group in the α-position have also been considered as potential PET radiotracers, with nucleophilc synthesis of racemic α-[18F]fluoromethyl phenylalanine using cyclic sulfamidate precursor already reported1. Recently our group has suggested a route towards enantiomerically pure α-[18F]fluoromethyl-p-tyrosine via direct nucleophilic fluorination of a NiII complex2. However, 18F-fluorination of the precursor bearing α-MeSO3 (mesyloxy) leaving group was inefficient, possibly due to the steric hindrance at the α-carbon of the tyrosine moiety. In continuation of that previous work we have investigated the synthesis of the α-[18F]fluoropropyl-L-DOPA via 18F-fluorination of a similar precursor.

**Materials and methods:** Radiofluorination of I was performed in the presence of kryptofix/K2CO3 (acetone, 10 min, 85oC, 10 mg of precursor). The extent of 18F-fluorination was followed by radioTLC (EtAc:CHCl3:CH3COOH 4:1:1). After solvent removal the 18F-fluorinated intermediate was treated with 6M aq. HCl at 140oC for 10 min. The product was analyzed by radioTLC (EtOH:n-Butanol:CH3COOH 4:1:1) and HPLC (Lichrosphere 100-RP-18, 250 x 4 mm, 0.1% CH3COOH).

**Results** The precursor, NiII complex of Schiff base of (2S)-1-benzyl-N-(2-formylphenyl)pyrrolidine-2-carboxamide (BBA) with (S)-2-amino-2-(3,4-bis((methylsulfonyl)oxy)benzyl)-5-((methylsulfonyl)oxy)pentanoic acid (Ni-(S)-BBA-(S)-MsO propyl-DOPA(OMs)2 (I)), was prepared from the corresponding NiII complex of BBA Schiff base with (S)-DOPA.18F-fluorination efficiency of I was 15%, with room for optimization. Acid hydrolysis of the intermediate afforded protected derivative of (S)-α-[18F]fluoropropyl-L-DOPA, but removal of the mesyl protective groups proved problematic - use of 2M potassium methylate in MeOH (60oC, 10 min) was ineffective.

**Discussion/conclusion** The nucleophilic fluorination of Ni^II^-based labeling precursor offers potential novel route towards a new derivative of DOPA with ^18^F-label in the α-fluoropropyl group. Preparation of precursor employing methoxy methoxy (MOM) protective groups instead of mesyl ones, something that should resolve the deprotection problem, is currently in progress.

This study was supported by SNF grant IZ73ZO_152360/1.

### PP15 A convenient one-pot synthesis of [18F]clofarabine

#### Revunov, Evgeny^1^; Malmquist, Jonas^1^; Johnström, Peter^1,2^; Van Valkenburgh, Juno^3^; Steele, Dalton^3^; Halldin, Christer^1^; Schou, Magnus^1,2^

##### ^1^Department of Clinical Neuroscience, Centre for Psychiatric ResearchKarolinska Institutet, Stockholm, Sweden; ^2^AstraZeneca Translational Science Centre, Stockholm, Sweden; ^3^Department of Medical and Molecular Pharmacology, Ahmanson Translational Imaging Division, University of California, Los Angeles, USA

###### **Correspondence:** Evgeny Revunov – Department of Clinical Neuroscience, Centre for Psychiatric Research, Karolinska Institutet, Stockholm, Sweden

**Introduction:** [18F]Clofarabine has recently emerged as a promising radioligand for in vivo imaging of deoxycytidine kinase activity using PET. In the reported protocols by Shu and Wu (1,2), 3 is prepared in a two-step process with an intermediate purification that proceeds with 10-15% radiochemical yield (RCY). We herein report a simplified procedure for the preparation of 3, in which the intermediate purification was eliminated.

**Materials and methods:** [18F]Fluoride was produced using a GE PETtrace cyclotron and dried azeotropically with acetonitrile (MeCN), potassium carbonate and kryptofix (K2.2.2). Radiofluorination was performed in MeCN at 90 oC for 20 minutes, after which solvents were removed in vacuo and deprotection was performed using 10% trifluoroacetic acid (TFA) in dichloromethane (DCM) at room termperature (rt) for 5 min. Following an additional evaporation, the crude product was dissolved in mobile phase and purified using semi-preparative HPLC (ACE, C18, 5μm, HIL 250×10 mm, eluted with MeCN: aq. NH4OH (0.6%) 90.5:9.5 (v/v) at 6 mL/min). 3 was isolated from the eluent using a C-18 SepPak cartridge (Waters) and formulated for intravenous injection in a solution of ethanol and phosphate buffered saline (10:90).

**Results:** The radiochemical conversion (RCC) of 18F-fluoride into 3 was between 25-30%. Higher reaction temperatures or prolonged heating did not improve the RCC. Removal of MeCN from the reaction mixture prior to deprotection was found to be essential and may be ascribed to the known inhibiting properties of MeCN on trityl-group hydrolysis (3). By-product formation, sometimes observed when refluxing 2 with aqueous HCl, could be effectively eliminated by employing mild conditions for the deprotection (10% TFA in DCM, rt, 5 min) without compromising the RCY. No intermediate purification was required in the process, that produced >99% radiochemically pure 3 in 20% RCY with a specific activity >100 GBq/μmol.

**Discussion/conclusion:** A simplified one-pot radiosynthesis of 3 was developed in which intermediate purification was eliminated. The RCY is on par with previously reported protocols for 3 synthesis.

### PP16 BODIPY-estradiol conjugates as multi-modality tumor imaging agents

#### Samira Osati^1^, Michel Paquette^1^, Simon Beaudoin^1^, Hasrat Ali^1^, Brigitte Guerin^1,2^, Jeffrey V. Leyton^1,2^, Johan E. van Lier^1,2^

##### ^1^Département de médecine nucléaire et radiobiologie, Faculté de médecine et sciences de la santé, Université de Sherbrooke, QC, Canada J1H5N4; ^2^Centre d’Imagerie Moléculaire de Sherbrooke (CIMS), CR-CHUS, Sherbrooke, QC, Canada J1H5N4

###### **Correspondence:** Johan E. van Lier – Département de médecine nucléaire et radiobiologie, Faculté de médecine et sciences de la santé, Université de Sherbrooke, QC, Canada J1H5N4

**Introduction**: *In vivo* imaging of estrogen receptor (ER) densities in human breast cancer is a potential tool to stage disease, guide treatment protocols and follow-up on treatment outcome. Among various techniques to detect ligand–ER interaction both positron emission tomography (PET) and fluorescence imaging have received ample attention.

**Materials and methods**: In this study we use 4,4-difluoro-4-bora-3a,4a-diaza-s-indacene (BODIPY) as a fluorescent probe and precursor for ^18^F-labelling to develop estradiol-based ligands for ER. The synthesis involves attachment of a BODIPY moiety to the C17α-position of estradiol using Sonogashira cross coupling reaction of iodo-BODIPY and various 17α-ethynyl estradiol (EE2) derivatives. Confocal laser scanning microscopy was used to monitor cellular uptake of the EE2 conjugates after 1 h of treatment without and with estradiol to block ER. Flow cytometric analysis was performed to determine the relative fluorescence in the nuclei. The relative binding affinities to the estrogen receptors (ERα and ERβ) was assessed by a competitive radiometric assay.

**Results:** The Sonogashira coupling reaction of EE2 derivatives with iodo-BODIPY(1:1 molar ratio) in THF/toluene (1/2) and using PdCl_2_(PPh_3_)_2_ as catalyst, NEt_3_ as a base and CuI at room temperature gave the desired EE2-BODIPY conjugates in 45-86% yield (after purification by silica gel column chromatography). *In vitro* fluorescence imaging of the basic EE2-BODIPY conjugate confirmed ER-mediated uptake by the nuclei of ER-positive MC7-L1cells.

**Discussion and conclusion:** The synthesis of a series of EE2-BODIPY conjugates was achieved via Sonogashira cross-coupling in moderate to very good isolated yields. Our *in vitro* nuclear cell uptake and receptor binding results warrant further studies to evaluate the potential of the EE2-BODIPY conjugates for fluorescence/PET imaging of breast cancer that overexpress ER. Fluor-18 labelling of the BODIPY moiety of the conjugate, and *in vivo* fluorescence/PET imaging studies, are in progress.

### PP17 Easy and high yielding synthesis of 68Ga-labelled HBED-PSMA and DOTA-PSMA by using a Modular-Lab Eazy automatic synthesizer

#### Di Iorio V^1^, Iori M^2^, Donati C^1^, Lanzetta V^1^, Capponi PC^2^, Rubagotti S^2^, Dreger T^3^, Kunkel F^3^, Asti M^2^

##### ^1^Radiopharmacy Nuclear Medicine Unit, IRST IRCCS, Meldola (FC), Italy; ^2^Nuclear Medicine Unit, IRCCS-Arcispedale Santa Maria Nuova, Reggio Emilia, Italy; ^3^Eckert & Ziegler Eurotope GmbH, Berlin, Germany

###### **Correspondence:** M. Asti **–** Nuclear Medicine Unit, IRCCS-Arcispedale Santa Maria Nuova, Reggio Emilia, Italy

**Introduction:** 68Ga-labelled PSMA inhibitor analogues are one the most important class of new radiotracers under clinical observation and development for the detection of prostate cancer lesions and metastases. 68Ga- PSMA-11 (68Ga-PSMA-HBED-CC) has already attested its suitability in the clinical practice and 68Ga-PSMA-617 (68Ga-DOTA-PSMA) has recently demonstrated its potential in individual first-in-man studies gaining importance above all in view of its theranostic application as it can be labelled also with 90-Yttrium and 177-Lutetium. In this study the two 68Ga-labelled radiotracers were synthesized using an automatic Modular-Lab Eazy synthesizer (Eckert & Ziegler) and the reliability of the system was validated to guarantee preparations of pharmaceutical grade.

**Materials and methods:** The system was assembled with a disposable cassettes and the vials were filled with commercial precursors and ready to use pharmaceutical grade reagents. After clicking the start button the following steps were performed: a 1850 MBq GalliaPharm 68Ge/68Ga generator (Eckert & Ziegler) was eluted with 0.1 M HCl by a peristaltic pump by passing through a cation exchange cartridge into a waste vial. The cartridge was dried and the blocked activity was eluted with 0.55 ml of a 5.5 M HCl/5 M NaCl solution into the reaction vial pre-filled with 40 ug of precursor (PSMA-11 or PSMA-617) and 2.6 ml of Sodium Acetate/HCl/EtOH buffer. The reactor was heated at 110°C for 4 (PSMA-11) or 8 (PSMA-617) minutes. The mixture was diluted with 7 ml of 0.9 % NaCl solution and transferred into the final product vial by passing through a light CM cartridge and a sterilizing filter.

**Results:** Both radiopharmaceuticals were produced in high yield. Starting from an activity of 700±20 MBq the radiochemical yield were 75.6±10 and 75.2±7 % for PSMA-11 and PSMA-617, respectively (n = 5) after 15 minutes. RCP was assessed by HPLC and was always > 98 % (for 68Ga-PSMA-11 the sum of the peaks of the two isomers was considered). All the preparations were sterile and the endotoxin content was < 0.5 EU/ml.

**Discussion/conclusions:** The Modular-Lab Eazy synthesizer works with a new technology which uses a pressure distribution system to transfer the liquid instead of solenoid valves or stopcocks. A disposable, easy to assemble cassette allows the synthesis of 68Ga-PSMA-11 and 68Ga-PSMA-617 in high RCY and quality.

### PP18 Synthesis and evaluation of fusarinine C-based octadentate bifunctional chelators for zirconium-89 labelling

#### Chuangyan Zhai^1^, Christine Rangger^1^, Dominik Summer^1^, Hubertus Haas^2^, Clemens Decristoforo^1^

##### ^1^Department of Nuclear Medicine, Medical University Innsbruck, Innsbruck, Austria; ^2^Division of Molecular Biology, Medical University Innsbruck, Innsbruck, Austria

###### **Correspondence:** Chuangyan Zhai – Department of Nuclear Medicine, Medical University Innsbruck, Innsbruck, Austria

**Introduction:** 89Zr has received considerable interest as a positron emitting radionuclide for immuno-PET imaging due to the excellent nuclear and physical properties. Recently we reported that fusarinine C (FSC) is a promising alternative chelator for 89Zr-based PET imaging agents, exhibiting superior stability and kinetic inertness as compared to 89Zr-desferrioxamine B ([89Zr]DFO).[1] Here we designed FSC derivatized chelators for 89Zr labeling which were expected to on the one hand improve the stability of 89Zr-complexes by saturating the 8 coordination sphere of 89Zr and on the other hand, reduce the number of functionality making it suitable for the conjugation to monoclonal antibodies.

**Materials and methods** FSC(succ)2 and FSC(succ)3 were synthesized by FSC reacting with succinic anhydride, and FSC(succ)2AA was synthesized by FSC(succ)2 reacting with acetic anhydride. The complexation properties of FSC(succ)2AA and FSC(succ)3 with Zr4+ were studied by reacting with ZrCl4. For in vitro evaluation partition coefficient, protein binding property, serum stability as well as acid dissociation and transchelation studies of 89Zr-complexes were evaluated and compared with [89Zr]DFO and 89Zr-triacetyfusarinine C ([89Zr]TAFC). The in vivo properties of [89Zr]FSC(succ)3 were further compared with [89Zr]TAFC in nude mice.

**Results** FSC(succ)2AA and FSC(succ)3 were synthesized with satisfying yield. The complexation with ZrCl4 was achieved using a simple strategy resulting in high-purity [natZr]FSC(succ)2AA and [natZr]FSC(succ)3 with a 1:1 stoichiometry. Distribution coefficient of 89Zr-complexes revealed improved hydrophilic characters compared to [89Zr]TAFC. All radioligands showed high stability in PBS and human serum and low protein-bound activity over a period of 7 days. Acid dissociation and transchelation studies exhibited the different in vitro stabilities following the order of [89Zr]FSC(succ)3 > [89Zr]TAFC > [89Zr]FSC(succ)2AA > [89Zr]DFO. Biodistribution study of [89Zr]FSC(succ)3 exhibited a slower excretion compared to [89Zr]TAFC.

**Conclusion [**89Zr]FSC(succ)3 showed best stability and inertness and [89Zr]FSC(succ)2AA predicts the potential of FSC(succ)2 as a monovalent chelator for the conjugation to targeted biomolecules in particular monoclonal antibodies.

### PP19 Fully automated production of [18F]NaF using a re-configuring FDG synthesis module

#### Suphansa Kijprayoon, Ananya Ruangma, Suthatip Ngokpol, Samart Tuamputsha

##### Oncology Imaging & Nuclear Medicine Department, Wattanosoth Hospital, Bangkok Hospital at Headquarter, Bangkok, Thailand

###### **Correspondence:** Suphansa Kijprayoon – Oncology Imaging & Nuclear Medicine Department, Wattanosoth Hospital, Bangkok Hospital at Headquarter, Bangkok, Thailand

**Introduction:** We modified a fully automated method for [18F]NaF synthesis by re-configuring a commercial FDG synthesis module (Single synthesis module, Advanced Cyclotron Systems, Inc.). **Materials and methods:** The Lookout program was used to sequence the steps for automated synthesis using excel spreadsheet. The [18F]fluoride solution is transferred to synthesis module. The [18F]fluoride ions are trapped in QMA. The QMA cartridge is then washed with sterile water for injection and eluted with 0.9% NaCl. The final product was passed through 0.22 μm sterile filter to sterile product vial.

**Results:** [18F]NaF was successfully produced consistently with high yield. The non-decay corrected yield after synthesis is at least 85%. Quality control of [18F]NaF is performed according to USP and EP requirements. The QC results are passed.

**Conclusions:** This modified fully automated synthesis showed reproducible and very good radiochemical yields. This module could be used for [18F]NaF production in our department.

### PP20 Extension of the Carbon-11 Small Labeling Agents Toolbox and Conjugate Addition

#### Ulrike Filp^1^, Anna Pees^1^, Carlotta Taddei^2^, Aleksandra Pekošak^1^, Antony D. Gee^2^, Alex J. Poot^1^, Albert D. Windhorst^1^

##### ^1^Radiology and Nuclear Medicine, VU University Medical Center, Amsterdam, The Netherlands; ^2^ Division of Imaging Sciences and Biomedical Engineering, King’s College, London, UK

###### **Correspondence:** Ulrike Filp – Radiology and Nuclear Medicine, VU University Medical Center, Amsterdam, The Netherlands

**Introduction:** [^11^C]CO_2_ and [^11^C]CO are undoubtedly highly versatile radiolabeling agents with many applications. The formation of [^11^C]CO from [^11^C]CO_2_ has become convenient and difficulties have been overcome for its further implementation in radiochemistry. Likewise, many reactions are being explored and many applications are already possible.^[1]^ However, there is still a need for expansion of the carbon-11 chemistry toolbox. We here present unprecedented Michael addition reactions with carbon-11 labeled synthons **1** – **3** as Michael donors.

**Methods:** The synthesis of [^11^C]Methyl acrylate **1** was performed with [^11^C]CO_2_ by fixation in a Grignard reaction with vinyl magnesium bromide (0.16M in THF) and subsequent Fisher esterification under acidic conditions. Alternatively, a palladium-mediated carbonylation reaction with [^11^C]CO ^[2]^ in the presence of *tert*-butanol or *trityl*-amine with vinyl-halides afforded [^11^C]*Tert*-butyl acrylate **2** and [^11^C]*Trityl*-acrylamide **3**. Michael addition reactions were performed with *N*-(diphenylmethylene)glycine *tert*-butyl ester **4** by adding the synthons to the reaction solution using TBAF as a base in DMSO. Synthon **1** was distilled into the reaction mixture. Synthons **2** and **3** were purified by solid-phase extraction and the Michael addition was performed at 100 °C for 5 min, respectively.

**Results:** The synthesis of **1** was successful with over 70% radiochemical conversion (RCC) and >95% radiochemical purity determined by HPLC. After screening and optimization **2** was obtained in 79 ± 10% and **3** in 73 ± 5% RCC. The subsequent conjugate addition yielded [^11^C]*N*-(diphenylmethylene)-glutamic acid 5-methyl 1-*tert* butyl ester **5** in 90 ± 5%, [^11^C]*N*-(diphenylmethylene)-glutamic acid di-*tert* butyl ester **6** in 93 ± 6% and [^11^C]*N*-(diphenylmethylene)-glutamine 5-*trityl* 1-*tert* butyl ester **7** in 78 ± 3% RCC.

**Conclusion:** The successful synthesis of **1 - 3** has been achieved in good radiochemical yields. Furthermore, conjugate addition to **5 - 7** is possible and we are currently working on expanding the variety of reactions with these small synthons.

**Acknowledgements:** RADIOMI FP7-PEOPLE-2012-ITN; [11C]CO2 provided by BV Cyclotron.

### PP21 In vitro studies on BBB penetration of pramipexole encapsulated theranostic liposomes for the therapy of Parkinson’s disease

#### Mine Silindir Gunay^1^**,** A. Yekta Ozer^1^, Suna Erdogan^1^, Ipek Baysal^3^, Denis Guilloteau^2^, Sylvie Chalon^2^

##### ^1^Department of Radiopharmacy, Faculty of Pharmacy, Hacettepe University, 06100, Ankara, Turkey; ^2^UMR INSERM U930,Université François Rabelais de Tours, Tours, France; ^3^Department of Biochemistry, Faculty of Pharmacy, Hacettepe University, 06100, Ankara, Turkey

###### **Correspondence:** Mine Silindir Gunay **–** Department of Radiopharmacy, Faculty of Pharmacy, Hacettepe University, 06100, Ankara, Turkey

**Introduction**: Brain penetration and targeting is hard due to complex structure with different barriers such as blood–brain barrier (BBB) with blood–cerebrospinal fluid (CSF) interface and CSF–blood interface. Although these tight and rigid barriers protect brain, they also prevent penetration of molecules, drugs and radiopharmaceuticals for diagnosis and therapy of many neurodegenerative diseases. Parkinson’s disease (PD) is assumed to be one of the most frequently observed neurodegenerative disease among geriatric disorders. PD comprises motor symptoms resulting from the death of dopamine generating cells in the substantia nigra. By using the imaging modalities such as single photon emission computed tomography (SPECT), the decline in the accumulation of specific radiotracer in the striatum can be detected. For PD treatment, limited brain penetration of drug is a major concern. Passively or actively targeted, nanosized drug delivery systems such as liposomes have different interests for either therapy or diagnosis. Recent studies generally depend on the development of new delivery systems, theranostics, in which diagnosis can be managed together with therapy by evaluating therapeutic effect.

**Materials and methods:** Theranostic liposomes were formulated by polyethylene glycole (PEG) coated, nanosized, either neutral or positively charged, ^99m^Tc labeled for SPECT imaging and pramipexole encapsulated for PD therapy. Their characterization and in vitro release kinetics were evaluated. In vitro penetration of both formulations was evaluated in a BBB cell co-culture model.

**Results:** Both neutral and positively charged liposomes showed proper characterization with about 10% encapsulation efficiency and around 100 nm particle sizes. All formulations fitted to the first-order release kinetics. Both formulations were found BBB permeable in cell culture studies with fluorescent images and fluorospectroscopy.

**Conclusion:** Promising characterization and release profiles were obtained with theranostic liposomes for both diagnosis and therapy of PD. Both neutral and positively charged formulations found BBB permeable in vitro. In vivo animal studies are continuing to obtain more accurate data. (The authors thank to Abdi Ibrahim Ilaç for Pramipexole. This study was supported by the grant of TUBITAK, Project No: 112S244).

### PP22 Factors affecting tumor uptake of 99mTc-HYNIC-VEGF165

#### Filippo Galli^1^, Marco Artico^2^, Samanta Taurone^2^, Enrica Bianchi^2^, Bruce D. Weintraub^3^, Mariusz Skudlinski^3^, Alberto Signore^1^

##### ^1^Nuclear Medicine Unit, Department of Medical-Surgical Sciences and of Translational Medicine, Faculty of Medicine and Psychology, “Sapienza” University of Rome, Rome, Italy; ^2^Department of Sensory Organs, Sapienza University of Rome, Rome, Italy; ^3^Trophogen Inc., Rockville, MD, USA

###### **Correspondence:** Filippo Galli **–** Nuclear Medicine Unit, Department of Medical-Surgical Sciences and of Translational Medicine, Faculty of Medicine and Psychology, “Sapienza” University of Rome, Rome, Italy

**Introduction:** Angiogenesis promotes tumor growth and metastatization and its principal effector is the vascular endothelial growth factor (VEGF) secreted by cancer cells and other components of tumor microenvironment. Nowadays, many anti-angiogenic therapies have been developed and radiolabelled VEGF analogues may provide a useful tool to non-invasively evaluate the efficacy of such drugs. Aim of the present study was to radiolabel the human VEGF-A165 analogue with 99mTechnetium (99mTc) and evaluate the expression of VEGFR in both cancer and endothelial cells in the tumor microenvironment by nuclear medicine imaging and immunohistochemistry.

**Material and methods:** The human VEGF-A165 analogue was radiolabelled with 99mTc using succinimidyl-6-hydrazinonicotinate hydrochloride as a bifunctional chelator. In vitro quality controls were performed to assess its retained structure and biological activity. In vivo studies included biodistribution studies and tumor targeting experiments in athymic nude CD-1 mice bearing xenograft tumors from ARO, K1 and HT29 cell lines. Immunohistochemistry was performed on excised tumors to evaluate VEGF and VEGF receptor (VEGFR) expression in the lesion and endothelial cells.

**Results:** The analogue was labelled with high labelling efficiency (>95%) and high specific activity, with retained biological activity and structural integrity. Tumor targeting experiments revealed a focal uptake of radiolabelled VEGF165 in tumor xenografts with different tumor-to-background ratios. Immunohistochemical analysis tumors revealed an inverse correlation between VEGF and uptake of the radioactive hormone. A positive correlation between radioactive VEGF165 and VEGFR1 was also observed.

**Conclusion:** Radiolabelled human VEGF-A165 is a promising radiopharmaceutical to image angiogenesis and evaluate the efficacy of anti-angiogenic drugs. However, VEGFR imaging may suffer from quenching effects of endogenous VEGF produced by cancer and other cells of the microenvironment.

### PP23 Rhenium-188: a suitable radioisotope for targeted radiotherapy

#### Nicolas Lepareur^1^**,** Nicolas Noiret^2^, François Hindré^3,4^, Franck Lacœuille^3,4^, Eric Benoist^5^, Etienne Garin^1^

##### ^1^Centre Eugene Marquis, Nuclear Medicine Department, INSERM UMR-S 991, 35042, Rennes, France; ^2^ENSCR, CNRS UMR 6226, 35708, Rennes, France; ^3^University of Angers, INSERM UMR-S 1066 MINT, 49100, Angers, France; ^4^University of Angers, SFR ICAT, PRIMEX, 49100, Angers, France; ^5^ Université Paul Sabatier, SPCMIB, UMR CNRS 5068, 31062 Toulouse, France

###### **Correspondence:** Nicolas Lepareur – Centre Eugene Marquis, Nuclear Medicine Department, INSERM UMR-S 991, 35042, Rennes, France

**Introduction:** Among radioisotopes for targeted therapeutic applications, 188Re is very promising, thanks to its suitable properties (β- emitter, Emax = 2.12 MeV, t1/2 = 17 h, γ-emission of 155 keV, conveniently obtained through a generator), and to the fact that it is a homologous element to 99mTc, the radioelement of choice in nuclear medicine. Inside the “Vectorisation & Radiotherapy” Axis of the Canceropole Grand Ouest and the IRON Labex, our teams have developed a strong expertise on the chemistry and targeting of rhenium labelled radiotracers, from bench to bedside. Some examples of what we have been developing are given below. **188Re-labelled Lipiodol:** We have developed a stable and efficient labelling of Lipiodol with 188Re as a potential treatment for HCC. A phase 1 clinical trial is currently running. **188Re-labelled particles:** 188Re has been used to label micro- and nanoparticles. Radiolabelled starch-based microspheres are investigated in HCC, while lipid nanocapsules, also investigated in HCC and adenocarcinoma models, gave prominent results in glioma, for which transfer to clinic is underway. **188Re-labelled peptides:** We are also developing a new bifunctional chelate for the Re(I) tricarbonyl core, able to link a biomolecule (peptide, protein…).

**Conclusions:** For more than 15 years, our teams, working closely together, have developed a series of potential therapeutic radiotracers, some of which are now investigated in man, or about to be.

### PP24 Preparation of a broad palette of 68Ga radiopharmaceuticals for clinical applications

#### Trejo-Ballado F, Zamora-Romo E, Manrique-Arias JC, Gama-Romero HM, Contreras-Castañon G, Tecuapetla-Chantes RG, Avila-Rodriguez MA

##### Unidad Radiofarmacia-Ciclotrón, Facultad de Medicina, Universidad Nacional Autónoma de México, México, D.F. 04510, México

###### **Correspondence:** Trejo-Ballado F **–** Unidad Radiofarmacia-Ciclotrón, Facultad de Medicina, Universidad Nacional Autónoma de México, México, D.F. 04510, México

**Introduction**: PET molecular imaging and receptor binding peptides are emerging as powerful tools for imaging. Somatostatin analogues labeled with 68Ga are the most widely used and have proven useful for the management of patients with neuroendocrine tumors. However, within the past few years several other receptor binding compounds labeled with 68Ga have shown promising results with potential clinical applications in PET centers that lack in site cyclotrons. The aim of this study was to develop a single vial kit solution for the production of 68Ga radiopharmaceuticals for clinical applications.

**Materials and methods:** Chemical precursors DOTA-NOC, DOTA-RGDfK dimer, DOTA-Ubiquicidin (29-41), and PSMA-11 were acquired from ABX. Gallium-68 was obtained from an ITG generator while labeling was performed in an iQS module (ITG GmbH). Stock solutions of different concentrations were prepared by dissolving the precursors in 0.25M NaOAc. Aliquots of 100 μl of the stock solutions were dispensed in Eppendorf vials and stored at -20°C. For labeling, the aliquots were diluted with 900 μl of 0.25M NaOAc, mixed with 4 ml of 68GaCl3, and incubated for 10 min. Purification was made by SPE eluting the product with 1ml 50% EtOH. Final product was diluted with physiological saline and sterilized by filtration (0.22 μm, Millex-GV). Radiochemical purity (RCP) was determined by gradient HPLC using a Nova-Pak C18 column (3.9 x 150 mm), 2.5 ml/min flow rate. Eluents were A=0.1N TFA and B=MeCN.

**Results:** Non-decay-corrected yields (mean±SD, n=10) were 60±12, 61±6, 56±10 and 70±10% for NOC, RGD, UBI and PSMA, respectively. RCP was >98% for all the radiopharmaceuticals. HPLC retention times were 0.5±0.1 min and 2.5±0.2 min for free ^68^GaCl_3_ and labeled compounds, respectively. No ^68^Ge was detected in the final product.

**Conclusion**: This labeling method using a single vial kit solution is simple, efficient and reliable, suitable to be used for the production of a variety of 68Ga radiopharmaceuticals for clinical diagnostic applications.

**Acknowledgments:** Research supported by CONACYT Grant 179218, and IAEA RC16467.

### PP25 68Ga-peptide preparation with the use of two 68Ge/68Ga-generators

#### H. Kvaternik, D. Hausberger, C. Zink, B. Rumpf, R. M. Aigner

##### Division of Nuclear Medicine, Department of Radiology, Medical University of Graz, Graz, Austria

###### **Correspondence:** H. Kvaternik **–** Division of Nuclear Medicine, Department of Radiology, Medical University of Graz, Graz, Austria

**Introduction:** The clinical demand for 68Ga-labelled peptides is constantly rising, but the achievable radioactivity per batch is limited by the inventory of the 68Ge/68Ga generator. One possible approach to raising the radioactive yield is to merge the radioactive contents of two 68Ge/68Ga generators for one batch of 68Ga-peptide. The key to this method can found in frame with the cationic purification (1) merging two generator eluates. Our aim was to establish this method in the synthesis of 68Ga-DOTANOC.

**Material and methods**: The experimental setup considered two 1.85 GBq (50 mCi) iThemba 68Ge/68Ga-generators with calibration Feb/15 and Oct/15 and a Scintomics GRP synthesis module. SC-01 peptide cassettes including the reagents were applied in the synthesis of 68Ga-DOTANOC. The automatic synthesis sequent was modified by us as followed: After the initial conditioning of the C18-SPE with ethanol, water and subsequent drying with N2 the “older” 68Ge/68Ga-generator was eluted with 7 mL 1M HCl by the motor syringe. 10 mL water was added for dilution, and the mixture was transferred over a PS-H+ SCX column (Machery-Nagel). The sequent stopped for the manual change of the generator tube. After continuing the sequent, the “newer” Generator was eluted in the same manner as described above. Once washing and drying the valve seats, 68Ga was delivered from PS-H+ into the reaction vessel with a solution of 1.4 mL 5M NaCl and 0.2 mL 6M HCl. The further labelling with 40 μg precursor in HEPES were performed as usual.

**Results:** We found in our preliminary experiments more the 98% of the theoretical 68Ga radioactivity after “double elution” adsorbed on the PS-H+ SCX cartridge. About 15 % of 68Ga retained on the PS-H+ SCX after transfer of the 68GaCl3 into the reaction vessel. The followed labelling of 68Ga-DOTANOC revealed an uncorrected yield of >40%.

**Conclusion:** The promising preliminary results with a yield of 1.1 GBq 68Ga-DOTANOC (EOS) in a merging operation of an “old” and a “new” 68Ge/68Ga generator indicates a high potential for improvement in routine preparation. Therefore, we extend our synthesis system with an additional valve actuator unit, which should handle the 68Ga elution from both generators automatically and under GMP conditions.

**Table 1 (abstract PP25):** See text for description

^68^Ga-DOTANOC preparation	Starting activity	^68^Ga-DOTANOC (EOS)	Synthesis-time	Yield (uncorr.)
Standard Single generator	1.73 GBq	0.87 GBq	32 min	50 %
Experimental Dual generator	2.56 GBq*	1.10 GBq	44 min	43 %

*Theoretical starting activity of both 68Ge/68Ga-generator corrected to elution start of the second one

### PP26 Assay of HEPES in 68Ga-peptides by HPLC

#### H. Kvaternik, D. Hausberger, B. Rumpf, R. M. Aigner

##### Division of Nuclear Medicine, Department of Radiology, Medical University of Graz, Graz, Austria

###### **Correspondence:** H. Kvaternik **–** Division of Nuclear Medicine, Department of Radiology, Medical University of Graz, Graz, Austria

**Introduction:** HEPES (2-[4-N-(2-hydroxyethyl)-1-piperazinyl]-N’-ethanesulfonic acid) is widely used as a “good” reaction buffer in the labelling of 68Ga-peptides. The final, injectable solution of 68Ga-peptide may contain traces of HEPES as an impurity. The European Pharmacopoeia limited the HEPES content in 68Ga-Edotreotide (68Ga-DOTATOC) with 200 μg per patient dose (1). This limit test is performed by TLC and developed by the exposing to iodine vapour. Alternatively, an HPLC method for HEPES has been published (2). Our aim was to approve this method of the quality control of 68Ga-peptides.

**Material and methods:** 68Ga-peptides (68Ga-DOTANOC, 68Ga-DOTATOC, 68Ga-DOTATATE) were synthesised with a Scintomics GRP synthesis module using SC-01 cassettes. The determination of HEPES was performed by HPLC Agilent 1260 equipped with a DAD detector on an Obelisc N column (4.6 x 150 mm, Sielc Technologies): isocratic eluent 20% ACN/80% water/0.05% phosphoric acid; 0.8 mL/min; UV detection at 195 nm.

**Results:** Due to the polar characteristic of the Obelisc N column, a good separation of the sulfonic acid HEPES was achieved with a retention time of about 9 min. A system suitability test with a solution of 100 μg/mL HEPES and 1 μg/mL gentisinic acid was introduced. The analysis method was successful validated according to ICH in a concentration range of HEPES from 10 μg/mL to 200 μg/mL. This assay of HEPES was applied effectively in the refinement of synthesis sequence to the 68Ga-peptides. The accurate measurement of HEPES did help us to optimise the automatic workup of the reaction mixture via C18-SPE, that we reach an HEPES content in the final solution below 10 μg/mL routinely. A disadvantage of the Obelisc N is that the HPLC column is very sensitive to alcohol and neutral media. To avoid damage, for the Obelisc N column an acid media of about pH 2 must use to store. Otherwise due to irreversible deactivation of groups on the column material the signals of NaCl from the sample will shift in the retention time and interfere with the signal of HEPES, which disables the interpretation of the analyse results.

**Conclusion:** We have demonstrated that HEPES impurities in 68Ga-peptides can analyse by HPLC with respect to the limit of the European Pharmacopoeia. We validated this analysis method and used it in the development of the preparation of 68Ga-peptides. The HEPES determination by HPLC replaces in our laboratory the test by TLC and is integrated into the parametric release of our routinely prepared 68Ga-peptides.

### PP27 Preparation, in vitro and in vivo evaluation of a 99mTc(I)-Diethyl Ester (S,S)-Ethylenediamine- N,N´-DI-2-(3-Cyclohexyl) Propionic acid as a target-specific radiopharmaceutical

#### Drina Janković^1^, Mladen Lakić^1^, Aleksandar Savić^2^, Slavica Ristić^3^, Nadežda Nikolić^1^, Aleksandar Vukadinović^1^, Tibor J. Sabo^2^, Sanja Vranješ-Đurić^1^

##### ^1^Laboratory for radioisotopes, Vinča Institute of Nuclear Sciences, University of Belgrade, Belgrade, Serbia; ^2^Chair of General and Inorganic Chemistry, Faculty of Chemistry, University of Belgrade, Belgrade, Serbia; ^3^Biomedical Research Center, R&D Institute, Galenika a.d., Belgrade, Serbia

###### **Correspondence:** Drina Janković – Laboratory for radioisotopes, Vinča Institute of Nuclear Sciences, University of Belgrade, Belgrade, Serbia

**Introduction:** The “organometallic” complexes have been used in the development of new diagnostic as well as therapeutic radiopharmaceuticals.1 The organometallic labeling approach has led to the creation of a precursor [M(H2O)3(CO)3]+ (M = Tc, Re), which exhibits several useful characteristics. These include the small size of the core, easy preparation with aqueous-based precursor kit formulations, and readily substituted water molecules of the precursor fac-[99mTc(H2O)3(CO)3]+ by a variety of functional groups.2 The aim of this study is to label diethyl ester (S,S)-ethylenediamine-N,N´-di-2-(3-cyclohexyl)propionic acid (L) with 99mTc(I)-tricarbonyl precursor. The stability of the formed complex and its in vitro and in vivo properties were investigated.

**Materials and methods:** 99mTc(I)-L complex was prepared by a two-step procedure involving the preparation of the precursor [99mTc(CO)3(H2O)3]+, followed by the addition of L. The labelling efficiency and challenge with histidine and cisteine were determined using gradient HLPC. The in vitro stability of the radiochemical complex was determined in saline and human plasma. TCA precipitation method for determining the percentage of complex bound to proteins was very useful. Lipophilicity measurements were done by solvent extraction method with n-octanol. The overall complex charge was determined by electrophoresis on paper strips (Whatman no. 1) after exposure to a constant voltage (300 V) in phosphate buffer (pH 7.4) for 1 h. Organ distribution studies were carried out on normal and tumor-bearing mice (c57).

**Results:** Radiolabeling yield was higher than 98% and remained high and practically unchanged at 24h post-labeling. 99mTc(I)-L complex was stable and resistant histidine challenges. The percentage of protein binding was 19.62±2.02%. It showed a lipophyilic character. Biodistribution data, 5 min post injection, showed a very high uptake in liver and intestine. The majority of the radioactivity of 99mTc(I)-L complex was eliminated via the liver into the intestine. The organ distribution in B16-melanoma-bearing mice showed increase in uptake by the tumor.

**Discussion/conclusion:** 99mTc(I)-L complex revealed high radiochemical purity and stability in vitro, without any measurable decomposition. A significant difference of 99mTc(I)-L complex uptake between melanoma model and the normal mice was observed. Biodistribution studies showed high tumor uptake in B16-melanoma-bearing mice. This study indicates that 99mTc(I)-L complex may be a potential agent for melanoma detection.

### PP28 90Y-labeled magnetite nanoparticles for possible application in cancer therapy

#### S. Vranješ-Đurić^1^, M. Radović^1^, D. Janković^1^, N. Nikolić^1^, G. F. Goya^2^, P. Calatayud^2^, V. Spasojević^1^, B. Antić^1^

##### ^1^Laboratory for radioisotopes, “Vinča” Institute of Nuclear Sciences, University of Belgrade, Belgrade, Serbia; ^2^Instituto de Nanociencia de Aragón (INA), University of Zaragoza, Zaragoza, Spain

###### **Correspondence:** S. Vranješ-Đurić **–** Laboratory for radioisotopes, “Vinča” Institute of Nuclear Sciences, University of Belgrade, Belgrade, Serbia

**Introduction:** The synergistic interaction between hyperthermia and radiation therapy is based on the heat effect that may make some cancer cells more sensitive to radiation or harm other cancer cells that radiation cannot damage. In the present work two different types of magnetic nanoparticles (MNPs), Fe3O4-Naked and polyethylene glycol 600 diacid functionalized Fe3O4 (Fe3O4-PEG600), were synthetized in order to compare their efficiency as radioactive vectors. They were 90Y-labeled for the potential use in localized hyperthermia-radionuclide therapy.

**Materials and methods:** MNPs were synthesized by co-precipitation of ferric and ferrous salts in a basic solution. Radiolabeling was performed by mixing 0.5 mL of aqueous Fe3O4-Naked and Fe3O4-PEG600 MNPs suspension with 37 MBq 90YCl3 (0.001 mL) and incubating at room temperature on a shaker for one hour. Radiolabeled particles were then separated from free 90Y activity by precipitation with the help of permanent magnet. Radiolabeled MNPs were further used for in vitro stability studies in saline and human serum, and in vivo biodistribution studies in normal Wistar rats.

**Discussion/conclusion:** The Fe3O4-PEG diacid MNPs showed high phase purity and favorable physicochemical and magnetic characteristics for in vivo use. The obtained SPA value for Fe3O4-PEG diacid MNPs (200 W/g) indicates that these MNPs could be used as heating agents for in situ magnetic fluid hyperthermia protocols. Besides, high labeling yield (97%) and in vitro and in vivo stability of 90Y-labeled-PEGylated magnetite nanoparticles create opportunities for their use in the combined radio-hyperthermia cancer treatment.

### PP29 Simplified automation of the GMP production of 68Ga-labelled peptides

#### David Goblet, Cristiana Gameiro, Neva Lazarova

##### IBA, Louvain-la-Neuve, Belgium

###### **Correspondence:** Cristiana Gameiro **–** IBA, Louvain-la-Neuve, Belgium

**Introduction:** Optimized automated process for the GMP production of 68Ga-DOTA-NOC and 68Ga-PSMA-11 using a cassette-based synthesizer (Synthera®, IBA, Louvain-la-Neuve, Belgium) in combination with a recently commercialized 68Ge/68Ga generator (Galli EoTM, IRE-Elit, Fleurus, Belgium) have been developed in this work.

**Materials and methods:** The generator outlet is connected to an unmodified FDG-type disposable cassette with the appropriate reagent set and loaded onto the synthesizer. Once the automated process is started, vacuum elution takes place using only 1.1 mL of a 0.1 M HCl solution from the generator integrated reservoir. No purification being required, Ga-68 activity is directly sent to the reaction vessel. DOTA-NOC labeling is conducted as follows: a solution of 50 μg peptide in 1 mL 250 mM acetate buffer at pH 5 is added and the mixture is heated at 120°C for 5 min. The reaction bulk is then loaded on an HLB cartridge and washed with 10 mL of water. 68Ga-DOTA-NOC is then eluted with 1 mL of EtOH/water 65:35 v/v mix followed by 1 mL of saline 0.9% and collected through a 0.22 μM Millex®-GV filter into a sterile vial containing 8 mL of saline 0.9%. PSMA-11 labeling is conducted as follows: a solution of 10 μg peptide in 1 mL 1.5 M acetate buffer at pH 4.5 is added and the mixture is heated at 95°C for 5 min. The reaction bulk is then loaded on a Sep-pak® Light C18 cartridge and washed with 10 mL of water. 68Ga-PSMA-11 is then eluted with 2 mL of EtOH/water 1:1 v/v mixture followed by 2 mL of phosphate buffered saline (PBS) and collected through a 0.22 μM Millex®-GV filter into a sterile vial containing 6 mL of PBS.

**Results:** 68Ga-DOTA-NOC is produced in r <20 min with 81.5±5.2 % radiochemical yield (RCY) (decay-corrected-d.c.) and 68Ga-PSMA-11 is produced in 13 min with 97.4±2.5 % RCY (d.c.). Reported process times include generator elution and formulation. In both cases, final products show high radiochemical purity (TLC > 97 % and 99 % respectively).

**Discussion/conclusion:** Automated processes for the production of both 68Ga-DOTA-NOC and 68Ga-PSMA-11 have been successfully achieved using a commercial synthesizer and a 68Ge/68Ga generator. The labeling procedures are straightforward and efficient, thanks to the low elution volume and high purity of the generator eluate (no need for fractionation or post-elution purification).

### PP30 Combining commercial production of multi-products in a GMP environment with Clinical & R&D activities

#### Cristiana Gameiro^1^, Ian Oxley^1^, Antero Abrunhosa^2^, Vasko Kramer^3^, Maria Vosjan^4^, Arnold Spaans^4^

##### ^1^IBA SA, Louvain-La-Neuve, Belgium; ^2^ICNAS, University of Coimbra, Coimbra, Portugal; ^3^PositronPharma S.A., Santigo, Chile; ^4^BV Cyclotron VU, Amsterdam, The Netherlands

###### **Correspondence:** Cristiana Gameiro **–** IBA SA, Louvain-La-Neuve, Belgium

**Introduction:** Mono-product facilities are turning to multi-product to cope with decreasing prices of [18F]-FDG (FDG) and to support R&D programs. The aim is to demonstrate that in the same facility and equipment, a busy research program can be safely integrated into commercial production. **Materials and methods:** To be able to safely combine both activities a risk-assessment should be designed to identify key control points. The use of a fully automated platform (e.g. IBA Synthera) and its disposable cassettes can prevent human errors, reduce risk of cross-contamination and improve reliability. A production schedule, training and labeling procedures should also be considered. **Results:** Positronpharma in Chile delivers FDG daily to several hospitals and produced > 1600 Ci FDG in the last 3 years. In addition, [18F]-(FCH, NaF, FET, FLT, F-MISO, DMFP), [68Ga]-DOTA-peptides and PSMA-11 have been implemented as well as two more INDs: [18F]-PR04MZ and MHMZ were translated into clinical studies. ICNAS is a research unit of the University of Coimbra that hosts a GMP-compliant PET production facility which supports clinical and pre-clinical R&D programs and supplies RPs to nearby hospitals. The unit is in operation since 2012 and currently has five radiopharmaceuticals authorized in the market (18F-: FDG, FCH, NaF) and 68Ga-DOTA-NOC. All are produced on IBA Synthera and together represent >2000 production cycles. An extensive R&D program is in place with plans for production of other [18F]-tracers (F-DOPA nucleophilic route, FMISO, FLT, FES), [68Ga]- (PSMA, DOTA-TOC) and [64Cu]- (ATSM). BV Cyclotron VU in Amsterdam is a private company with a GMP-compliant PET production facility. Since the 90’s FDG is delivered for the Dutch hospitals (annual output of > 7300 patient doses (11 TBq)). FCH and FBB (Florbetaben) are also commercially produced with annual output >1400 (1.7 TBq) and >340 patient doses (1.1 TBq), respectively. Besides the commercial productions there is also room for an R&D program, e.g., currently improvement of the production of FCH is examined.

**Discussion/conclusion:** As a conclusion, the sites described have been functioning for several years and are able to safely combine commercial daily production while keeping a highly active R&D program with more than ten different tracers developed for pre-clinical and clinical applications.

### PP31 ^99^mTc(CO)3-labeling and Comparative In-Vivo Evaluation of Two Clicked cRGDfK Peptide Derivatives

#### Kusum Vats^1^, Drishty Satpati^1^, Haladhar D Sarma^2^, Sharmila Banerjee^1^

##### ^1^Radiopharmaceuticals Chemistry Section, Bhabha Atomic Research Centre, Trombay, Mumbai 400085, India; ^2^Radiation Biology and Health Science Division, Bhabha Atomic Research Centre, Trombay, Mumbai 400085, India

###### **Correspondence:** Drishty Satpati **–** Radiopharmaceuticals Chemistry Section, Bhabha Atomic Research Centre, Trombay, Mumbai 400085, India

**Introduction:** There is a continued interest for development of radiolabeled RGD peptides as integrin αvβ3 receptor targeted molecular probes possessing favorable pharmacokinetic behavior.1,2 In this study, two click chemistry modified cRGDfK peptide analogues have been synthesized 3 and radiolabeled with [99mTc(CO)3(H2O)3]+ precursor. Comparative in vivo evaluation of the two radiotracers was carried out to determine the pharmacokinetics and integrin αvβ3 targeting potential.

**Materials and methods:** cRGDfK was functionalized with N3CH2COOH and N3PEG7COOH at ε-amino group of lysine. The two azide-modified derivatives (with PEG7and without) were then clicked with propargyl glycine to introduce a tridentate chelating unit for radiolabeling with [99mTc(CO)3(H2O)3]+ precursor. The radiotracers, 99mTc(CO)3-Pra-Tz-CH2-cRGDfK and 99mTc(CO)3-Pra-Tz-PEG7-cRGDfK were analyzed by HPLC. Analogous rhenium complexes were synthesized for characterization of 99mTc(CO)3-labeled radiotracers. Biodistribution study of the two radiotracers was carried out in C57BL/6 mice bearing αvβ3-positive melanoma tumors. The radioactive preparation (~3.7 MBq/animal, 100 μL) was injected intravenously through the lateral tail vein. At different time intervals after injection (30, 60, and 180 min), the animals (n = 4/time point) were sacrificed and the relevant organs excised for measurement of retained activity**.**

**Results:** The two radiotracers, 99mTc(CO)3-Pra-Tz-CH2-cRGDfK and 99mTc(CO)3-Pra-Tz-PEG7-cRGDfK were prepared in >90% radiochemical yield and exhibited excellent stability in saline as well as in serum. Maximum tumor uptake was observed at 30 min p.i. for the two radiotracers and was higher for 99mTc(CO)3-Pra-Tz-PEG7-cRGDfK (4.11 ± 0.51 %ID/g) as compared to that for 99mTc(CO)3-Pra-Tz-CH2-cRGDfK (3.01 ± 0.77 %ID/g). Tumor accumulation of both the radiotracers reduced to 50-60% during blocking studies with cold cRGDfK peptide, suggesting receptor-mediated uptake of radiotracers.

**Conclusion:** The two neutral 99mTc(CO)3 radiotracers prepared exhibited receptor mediated uptake in melanoma tumor. Increased tumor uptake on introduction of PEG7 unit was compromised with slow clearance from other organs. Further modification of the peptide with a different spacer or a smaller PEG unit may lead to more favorable pharmacokinetics.

### PP32 Application of AnaLig resin for 99mTc separation from molybdenum excess

#### Wojdowska W., Pawlak D.W., Parus L. J., Garnuszek P., Mikołajczak R.

##### Radioisotope Centre POLATOM, National Centre for Nuclear Research, Andrzeja Sołtana 7, 05-400 Otwock, Poland

###### **Correspondence:** Wojdowska W. **–** Radioisotope Centre POLATOM, National Centre for Nuclear Research, Andrzeja Sołtana 7, 05-400 Otwock, Poland

**Introduction:** The shortage of 99Mo (fission) has renewed interest in alternative technetium-99m production methods either in nuclear reactor starting from 98Mo or in accelerator with 100Mo as a target material. Adsorption chromatography on alumina is not sufficient for alternative production methods due to high amount of Mo in the dissolved target solution. Several chromatographic resins were described for the separation of pertechneate from molybdate including Dowex or ABEC. Here we focused our attention on application of AnaLigTc-02 resin for efficient recovery of 99mTc from large molybdenum excess.

**Methods:** To simulate a target dissolution mixture, 10 mL of sodium molybdate solution and a small amount of Na99mTcO4 in NaOH or (NH4)2CO3 was delivered on the column containing 100 mg of AnaLig resin using the flow rate of 0.4 mL/min. Before 99mTc elution, the column was fed with 3 mL of sodium hydroxide or ammonium carbonate solution and subsequently with 1.5 mL of water. The 99mTc was recovered in 5 mL of water. The eluate and other effluents activities (one after introduction of Mo solution and two after rinsing columns in term with NaOH or (NH4)2CO3 solution and water) were measured with the dose calibrator Capintec CRC-55tR. The sorption yield was studied as a function of NaOH and (NH4)2CO3 for concentration of 1.05, 1.4, 2.8 and 4.2 M for the former and 0.5, 1.5 and 2.5 M for the latter.

**Results:** Best 99mTc recovery was obtained in 2.8M NaOH or 1.5M (NH4)2CO3 solution. Under these conditions, more than 86% adsorption and elution were achieved. Most 99mTc losses occurred during rinsing the column with water, even approaching 10% of activity and due to rinsing the column with NaOH or (NH4)2CO3 after sorption Tc-99m on Analig resin (up to 3.5%). The activity retained on the column was around 1% for solution in NaOH and about 3% for (NH4)2CO3, respectively.

**Acknowledgements:** This project was supported by the grant ALTECH PBS1/A9/2/2012 awarded by the National Centre for Research and Development in Poland within the Applied Science Program.

### PP33 Constraints for selection of suitable precursor for one-step automated synthesis of [18F]FECNT, the dopamine transporter ligand

#### Pijarowska-Kruszyna J^1^**,** Jaron A^1^, Kachniarz A^2^, Malkowski B^2^, Garnuszek P^1^, Mikolajczak R^1^

##### ^1^Radioisotope Centre POLATOM, National Centre for Nuclear Research, Otwock, Poland; ^2^Department of Nuclear Medicine, Oncology Centre prof. Lukaszczyk Memorial Hospital, Bydgoszcz, Poland

**Introduction**: The fluorine-18 labelled nortropane derivative 2β-carbomethoxy-3β-(4-chlorophenyl)-8-(2-fluoroethyl)-nortropane ([18F]FECNT) is a Positron Emission Tomography (PET) radioligand of reference for imaging the dopamine transporter (DAT). The use of this radioligand as a clinical marker is highly dependent on the robustness of the radiochemical process used for its preparation. [18F]FECNT has been previously synthesized in a two-step chemical process with 16% decay-corrected yield in total time of 150 min. Recently, more effective (25-33% decay-corrected yield, 80-90 min) one-step synthesis of [18F]FECNT by direct nucleophilic [18F]fluorination from N-mesyloxy precursor was reported in literature. However, the use of a mesylate as a leaving group in such a nucleophilic substitution reaction has some drawbacks. Mesylates are rather hard to detect using UV-absorption, which complicates the final HPLC purification of the radiotracer and also characterized by higher susceptibility to decomposition. Herein, we reported a fully automated synthesis of [18F]FECNT from the chlorinated precursor, which was earlier found to be the most stable of the studied halo- and sulfonyloxy- analogues.

**Materials and methods:** The commercially available SynChrom [18F] R&D synthesis module was readily configured for the one-step synthesis according to conditions achieved for the fluorination with non-radioactive fluoride. The radiolabelling process involved a classical [18F]fluoride nucleophilic substitution from the chlorinated precursor (5mg) was performed at 110°C for 12 min. Crude product was purified using preparative HPLC and SPE methods.

**Results:** The obtained yields for a chlorine-for-[18F]fluorine substitution of 59±12% (n=5) were higher than those (45-48%) reported for the [18F]FECNT batches prepared from the N-mesyloxy precursor. The total synthesis time was 80-90 min and [18F]FECNT batches of about 2.0±0.5 GBq (n=3) with high radiochemical purity over than 99% were obtained with 32±7% overall decay-corrected yields. These yields were comparable with previously reported results for the one-step synthesis of [18F]FECNT and significantly higher than yields obtained in two-step syntheses [2]. The specific activity of the final [18F]FECNT product was 55 GBq/μmol, which is consistent with literature synthesis results (38-72 GBq/μmol) of this radiotracer and sufficient for the DAT density determination in the human PET imaging studies.

**Discussion/conclusions:** In this study, the new chlorinated precursor was successfully used for one-step automated radiosynthesis of [18F]FECNT. Considering these promising results as well as long- term storage stability of this precursor, reported procedure may provide a straightforward and reliable method for the production of radiotracer suitable for human use.

### PP34 Gamma scintigraphy studies with 99mTc- amoxicillin sodium in bacterially infected and sterile inflamed rats

#### Derya Ilem-Ozdemir, Oya Caglayan-Orumlu, Makbule Asikoglu

##### Faculty of Pharmacy, Department of Radiopharmacy, Ege University, Bornova, Izmir, Turkey

###### **Correspondence:** Derya Ilem-Ozdemir **–** Ege University, Faculty of Pharmacy, Department of Radiopharmacy, Ege University, Bornova, Izmir, Turkey

**Introduction:** The in-time diagnosis of bacterial infection is extremely significant for the accurate treatment. Nuclear medicine scintigraphic imaging is an excellent non-invasive method of whole-body scanning, allow to determinate infectious foci in all parts of the body, based on pathophysiological changes which occur earlier in the infection process. The aim of this study was to perform gamma scintigraphy studies with bacterial infected and sterile inflamed rats with newly developed radiopharmaceutical 99mTc-amoxicillin sodium (99mTc-AMOX).

**Method:** E.coli suspension was intramuscularly injected into the right thigh muscle of rats to create infection model. Turpentine oil was intramuscularly injected into the left thigh muscle of rats to create sterile inflammation model. After the infection and sterile inflammation focus were allowed to develop for 24 hours swelling appeared and gamma scintigraphy studies were performed. 99mTc-AMOX (3.7MBq) was intravenously injected via tail vein to the rats. After administration of radiopharmaceuticals, serial static images were acquired at different time intervals (5 minutes, 1, 2, 3, 4 and 5 hours post injection).

**Results:** The uptake of 99mTc-AMOX following intravenous administrations was assessed on static images. The images depicted rapid distribution throughout the body and uptake in the bacterial infected and sterile inflamed thigh muscle within one hour after injection. There was a higher activity in both bacterial infected and sterile inflamed thigh muscle as compared to healthy thigh muscle. For quantitative evaluation, regions of interest were drawn around the target (infected and inflamed thigh muscle) and non-target (healthy thigh muscle) regions of the rats. The 99mTc-AMOX uptake was calculated by dividing the average counts per pixel within the region of target to the average counts per pixel within the region of non-target.

**Conclusion:** Based on the in vivo studies, 99mTc-AMOX has higher uptake in infected and inflamed thigh muscle than healthy muscle. The data suggest that 99mTc-AMOX remained at the infection and inflammation foci during the whole experiments, there being no statistically significant differences in the ratios during the studied period (p≥0.05).

### PP35 Preparation of 99mTc- Amoxicillin Sodium Lyophilized Kit

#### Derya Ilem-Ozdemir, Oya Caglayan-Orumlu, Makbule Asikoglu

##### Faculty of Pharmacy, Department of Radiopharmacy, Ege University, Bornova, Izmir, Turkey

###### **Correspondence:** Derya Ilem-Ozdemir **–** Ege University, Faculty of Pharmacy, Department of Radiopharmacy, Ege University, Bornova, Izmir, Turkey

**Introduction:** Infection detection by nuclear medicine imaging techniques based on pathophysiological changes which appear much earlier than anatomical changes in the infection process (1,2). To develop a better infection imaging which will accumulate efficiently to inflammatory foci, clear rapidly from background tissue, discriminate between bacterial infection and sterile inflammation, cost low and prepare easily we labeled amoxicillin sodium (AMOX) with 99mTc. The aim of this study is to prepare 99mTc-AMOX in a simple method with good labeling efficiency and evaluate the ready to use cold kit formulation thus making it available to the other nuclear medicine centers. The stability of 99mTc-AMOX in human serum was identified; sterility and pyrogenicity of the radiopharmaceutical was estimated.

**Method:** To investigate the optimum radiolabeling conditions, radiolabeling was tested with different concentrations of reducing (stannous chloride and stannous tartrate) and antioxidant (ascorbic acid) agent. Radiochemical analysis was performed with Radio Thin Layer Chromatography (RTLC) and Radio High Performance Liquid Chromatography (RHPLC) studies. Two different freeze dry kits were formulated with optimum labeling conditions and stability, sterility and pyrogenicity of the kits were performed.

**Results:** The effect of reducing agent concentration on the radiochemical purity was evaluated and optimum reducing agent amount found 200 μg. Under these conditions labeling efficiency was around 90%. In the presence of ascorbic acid, stability of the complex was slightly increase while labeling efficiency for early hours was not affected significantly. According to the experiments, the formation of the 99mTc-AMOX complex was very fast and reached the radiochemical purity over 95 % in 15 min after radiolabeling. While keeping other reaction conditions constant and varied the pH of the reaction from 4.8 to 7.4 radiochemical purity was slightly decreased. 99mTc- AMOX was found stable during to 24 hours incubation in saline and human serum. Kits were labeled with 37MBq, 185MBq and 370 MBq 99mTc. Slightly decrease in radiochemical purity was observed with increasing of radioactivity. According to sterility test, since there was no growth in batches, kits were sterile. Also gel clot test showed that kits were pyrogen free. The freeze dry kits developed in this study were found to be stable with a shelf–life of six months when preserved at both at +5±3°C and +25±2°C/60 ±5% RH.

**Conclusion:** Simple method for radiolabeling of AMOX with 99mTc has been developed and standardized. Labeling efficiency of 99mTc-AMOX was assessed by both RTLC and RHPLC and found higher than 90%. The resulting complex was quite stable and labeling of ˃90% was maintained for up to 6 hours. Two different freeze dry kits was developed and evaluated. Based on the data obtained from this study, both products was stable for 6 months with high labeling efficiency.

### PP36 Outfits of Tracerlan FXC-PRO for 11C-Labeling

#### Arponen Eveliina, Helin Semi, Saarinen Timo, Vauhkala Simo, Kokkomäki Esa, Lehikoinen Pertti

##### Radiopharmaceutical Chemistry Laboratory, Turku PET Centre, University of Turku, Turku, Finland

###### **Correspondence:** Arponen Eveliina – Radiopharmaceutical Chemistry Laboratory, Turku PET Centre, University of Turku, Turku, Finland

**Introduction:** Commercial synthesis modules for ^11^C-labeling are not often directly suitable for the production of different labeling agents. Modification of Tracerlab FX_C-Pro_ synthesis module to perform both [^11^C]methylation and [^11^C]carboxylation^1^ and the set-up of [^11^C]radiopharmaceuticals for clinical use are described.

**Materials and methods:** Commercial GE Tracerlab FX_C-Pro_ synthesizer was modified to enable ^11^C-methylation with both [^11^C]methyl iodide and [^11^C]methyl triflate as well as direct ^11^C-carboxylation with [^11^C]CO_2_. Additional magnetic valves were installed to bypass the [^11^C]methyl iodide recycling system for direct use of [^11^C]CO_2_ and to bypass the [^11^C]methyl triflate oven when using [^11^C]methyl iodide as such. The valves are operated by Tracerlab software. The HPLC operations were modified by installing pneumatic column selector and a syringe operated loop injector. A sterile filtration unit (SFU) was coupled with synthesizer to allow online filter integrity test. For monitoring purposes more GM tubes were also installed. [^11^C]PK11195 was labeled with [^11^C]methyl iodide while [^11^C]methionine and [^11^C]metomidate were labeled with [^11^C]methyl triflate. [^11^C]Acetate was synthesized using direct carboxylation with [^11^C]CO_2_. Semi-preparative HPLC was used for the purification of [^11^C]PK11195 and [^11^C]methionine. Solid phase extraction cartridges were used for the purification of [^11^C]metomidate and [^11^C]acetate.

**Results**: The average synthesis times were 30 minutes for [^11^C]methionine, 38 minutes for [^11^C]PK11195, 20 minutes for [^11^C]metomidate and [^11^C]acetate including purification, formulation and online sterile filter integrity test. [^11^C]Radiopharmaceuticals were obtained in good radiochemical yields, sufficiently for several simultaneous clinical doses. The radiochemical purity exceeded 95% in all syntheses. The use of column selector enables easy change of HPLC columns between syntheses. The filling of the injector loop with pneumatic syringe decreases losses of the injection solutions compared to the inbuilt fluid detector. Due to the limited number of electrical connections in Tracerlab FX_C-Pro_ the column selector, loop syringe and SFU are remote controlled from external touch screen. Readings from additional GM tubes are also shown on the touch screen.

**Conclusion:** Reliable and robust routine procedures for the production of [^11^C]methionine, [^11^C]PK11195, [^11^C]metomidate and [^11^C]acetate were developed using a modified Tracerlab FX_C-Pro_ module. The synthesis module can be easily modified and re-modified for production of many different [^11^C]radiopharmaceuticals.

### PP37 Microfluidic synthesis of ω-[18F]fluoro-1-alkynes

#### Mariarosaria De Simone^1^, Giancarlo Pascali^2^, Ludovica Carzoli^1^, Mauro Quaglierini^1^, Mauro Telleschi^1^, Piero A. Salvadori^1^

##### ^1^CNR Institute Clinical Physiology Pisa, Pisa, Italy; ^2^Life Sciences, Australian Nuclear Science and Technology Organisation, New Illawarra Road, Lucas Heights NSW 2234, Australia

###### **Correspondence:** Mariarosaria De Simone **–** CNR Institute Clinical Physiology Pisa, Italy

**Introduction:** Cu(I)-catalyzed 1,3-dipolar cycloadditions is widely used in labeling azido-functionalized peptides and small-molecules with18F-alkynes. We report on the microfluidic (ADVION) synthesis of 5-[18F]fluoro-1-pentyne (5-[18F]-FP) and 6-[18F]fluoro-1-hexyne (6-[18F]-FH) and application in labeling an azide-functionalized Factor-H.

**Materials and methods:** 5-tosyloxy-1-pentyne (5-TP) and 6-tosyloxy-1-hexyne (6-TH) were obtained from the corresponding alcohols [1]. [18F]fluoride (0.3 GBq) was produced by irradiation of >98% 18O-water (nyobium target), trapped on a trap&release column and eluted with K222/K2CO3 (15mg/3mg) in dry acetonitrile (1ml). The K222/K[18F]F complex was dried by azeotropic distillation in the Concentrator module, recovered in the reaction solvent (600ul) and uploaded in a storage loop (P3). 5-TP or 6-TH was dissolved in 1 ml of solvent and uploaded in a storage loop (P1). Boluses of precursor and K222/K[18F]F solutions were pushed (total flow 40μL/min; P1=P3) through the reactor (2m fused-silica coil-reactor i.d.=100μm, 15.6μl) at 90, 110, 130, 150 and 170°C Experiments were repeated in acetonitrile, dimethylsulfoxide, tert-butanol and different P1:P3 ratios (1:1 and 1:5). Products were purified by distillation and analyzed (radio-TLC, radio-HPLC). Factor-H was reacted with an azido-(PEG)4-carboxylate linker (azido-PEG) targeting protein amino groups. Factor-H (1.6nmol in 250μl of pH 9 PBS buffer), was reacted (4h at RT) with 48nmol of NHS-activated azido-PEG. The azido-protein was purified by ultracentrifugation, suspended in water and lyophilized. 5-[18F]-FP was distilled into a vial containing 2-6nmol of azidoPEG-Factor-H, 1.4 μmol of Cu(I), 1.9μmol of TBTA in 150μl of THF/H2O (2:1) and reacted at 45°C for 10'. Unreacted 5-[18F]FP was removed under nitrogen flow. 5-[18F]-FP-Factor-H was purified by gel filtration (RCP> 97%) and the yield (92±3% d.c.y., n=5) determined by HPLC.

**Results:** Best yield for 5-[18F]-FP and 6-[18F]-FH (91±6% and 83±5% d.c.y, n=3) were obtained in acetonitrile (130°C), P1:P3 1:1. Reaction yield was lower in DMSO for both 5-[18F]-FP and 6-[18F]-FH (68±11% and 71±9%, d.c.y, n=3) and worse in tert-butanol (33±5% and 45±2%, d.c.y, n=3). Increasing P1:P3 ratio did not influence yield and resulted in a more frequent reactor clogging**.**

**Discussion/conclusion:** We describe a microfluidic approach for the synthesis of C5 and C6 18F-alkynes and the on-line radiolabelling of an azido-functionalized Factor-H with 5-[18F]-FP. The proposed method is applicable to a broad group of compounds (small molecules, peptides and nanoparticles).

**References:** 1 - Wang, Zhe et al., (2000), J. Org. Chem. 65(6):1889-1891.

### PP38 Automated 18F-flumazenil production using chemically resistant disposable cassettes

#### Phoebe Lam, Martina Aistleitner, Reinhard Eichinger, Christoph Artner

##### IASON GmbH, Feldkirchnerstraße 4, A-8054 Graz-Seiersberg, Austria

###### **Correspondence:** Phoebe Lam **–** IASON GmbH, Feldkirchnerstraße 4, A-8054 Graz-Seiersberg, Austria

**Introduction:** Production of 18F-flumazenil from 2-nitroflumazenil under GMP conditions is primarily performed with manipulators or custom built radiosynthesis modules with fixed components, as the use of N,N-dimethylformamide (DMF) at 160°C prohibits the use of commonly available disposable cassettes. Cassettes for the GE TRACERlab MXFDG synthesis modules are commercially available in different materials. ROTEM Industries and GE Healthcare use polysulfone as the main component, and it is known that this polymer is not compatible with DMF. Cassettes from ABX are reportedly more chemical resistant, with polypropylene as its main constituent. The aim of this work was to establish a robust automated radiosynthesis procedure for 18F-flumazenil on the GE TRACERlab MXFDG, where staff previously trained on the same equipment can easily perform the radiosynthesis.

**Materials and methods:** Fragments of the ABX FDG cassette were exposed for 1 or 24 h to various solvents including acetonitrile and DMF at room temperature (RT) or 60°C to assess its chemical resistance. A new synthesis sequence was written for the GE TRACERlab MXFDG. A non-radioactive test run was performed with the ABX FDG cassette, and radiosynthesis was carried out with the cassette components replaced by chemically resistance manifolds (ABX).

**Results:** Incubation of the PP ABX FDG cassette fragments in acetonitrile at RT showed no macroscopic influence on the polymer while the fragment became soft and mouldable at 60°C. Visible changes occurred after 1 h incubation with DMF, with the polymer having completely dissolved after 24 h, regardless of temperature. To verify the implication of these results on a 1 h radiosynthesis, a test run was performed using the same cassette material and synthesis conditions without radioactivity. Parts of the manifold was found to have cracked, and a valve blockade was observed due to polymer melting. Radiosynthesis was carried out with the manifolds replaced by chemically resistant ones, and 18F-flumazenil was successfully obtained in 4 % decay corrected yield.

**Conclusions:** Disposable cassettes assembled with chemically resistant manifolds is a requirement for the automated synthesis of 18F-flumazenil from 2-nitroflumazenil using the GE TRACERlab MXFDG synthesis module. Neither reagent kits nor program sequences are hitherto commercially available, but GMP grade production can nonetheless be successfully carried out with modifications on existing equipment.

### PP39 The effect of the eluent solutions (TBAHCO3, Kryptand K2.2.2) on the radiochemical yields of 18F-Fluoromethylcholine

#### Surendra Nakka, Hemantha Kumara MC, Al-Qahtani Mohammed

##### Cyclotron and Radiopharmaceuticals Department, King Faisal Specialist Hospital and Research Center, Riyadh, Saudi Arabia

###### **Correspondence:** Al-Qahtani Mohammed **–** Cyclotron and Radiopharmaceuticals Department, King Faisal Specialist Hospital and Research Center, Riyadh, Saudi Arabia

**Introduction:** [18F]Fluoromethylcholine ([18F]FCH) proves to be a unique radiotracer for the detection of prostate cancer as well as brain and lung tumors with Positron Emission Tomography (PET). The aim of this work was to improve radiochemical yields of 18F-Fluoromethylcholine ([18F]FCH) with high reproducibility. The used eluent solution plays an important role in production of [18F]FCH radiotracers. [18F]FCH is manufactured under GMP conditions using one of two eluent solutions namely Tetrabutylammonium Bicarbonate (TBAHCO3) and Kryptand 2.2.2., each time.

**Method:** [18F]FCH was radiosynthesized using a fully automated synthesis module TRACERlab MX. Hardware cassette and reagents kit purchased from ABX..[18F]FCH is synthesized by 18F-Fluoroalkylation of N-N-Dimethylaminoethanol (DMAE) in presence of Dimethyl Sulfoxide (DMSO) using 18F-Fluorobromomethane [18F]FBM as an alkylating agent.

**Results:** [18F]Fluoromethylcholine radiosynthesis assisted by Kryptand 2.2.2 shown irregular or poor distillation of 18F-Fluorobromomethane, which is observed by pressure trending window on TRACERlab MX during distillation step very poor gas flow through the four silica cartridges been observed which in turn leads to production of very less amounts of [18F]FCH. Tetrabutylammonium Bicarbonate (TBAHCO3) assisted 18F-FCH radiosynthesis observed with regular and continuous distillation of 18F-Fluorobromomethane through the four silica cartridges. [18F]FCH solution proved high radiochemical purities (>99%), Residual solvents analyzed using Gas Chromatography (GC), DBM & DMAE is not identified.

**Conclusion**: Choice of Tetrabutylammonium Bicarbonate (TBAHCO3) as eluent solution shown very consistent and improved radiochemical yields decay corrected 20-25% over Kryptand 2.2.2 as eluent solution.

### PP40 [68Ga]Radiolabeling of short peptide that has a PET imaging potentials

#### Al-Qahtani, Mohammed, Al-Malki, Yousif

##### Cyclotron and Radiopharmaceuticals Department, King Faisal Specialist Hospital and Research Center, Riyadh, Saudi Arabia

###### **Correspondence:** Al-Qahtani, Mohammed **–** Cyclotron and Radiopharmaceuticals Department, King Faisal Specialist Hospital and Research Center, Riyadh, Saudi Arabia

**Introduction:** Targeting short peptides are gaining interests in the molecular imaging field. Choosing the radioisotope to label those peptides is a challenging stage in the process of identifying the right imaging modality.

**Method:** All needed reagents and solvents were used with no further purifications unless it’s necessary. Peptide used was purchased from GenScript and used without further purifications. Reaction progress was monitored using both radio-analytical thin-layer chromatography (Radio-TLC) and high pressure liquid chromatographic (Radio-HPLC). Radio-HPLC analyses were carried out on semi-preparative Phenomenex C-18 (250 mm x 10 mm). Radiolabeled peptide was detected with the eluting of 0.065% TFA in water and 0.05% TFA in MeCN at 2 ml/min applying gradient elution mode. Radiochemical yields were calculated based on the Radio-HPLC collections. Sodium Citrate Buffer (PH=7.5) wa the Radio-TLC mobile phase.

**Synthesis of DOTA- Peptide Conjugate:** 9.97 E-7 mol of the intended peptide dissolved in DMSO reacts with 1.18 E-6 mol of (DOTA-NHS-ester) that was prepared in MeCN. The reaction was done in a phosphate buffer with PH=8 and heating at 90°C for 30min.

**68G -labeling of DOTA-Peptide:** 68G solution was added to 60 μg of conjugated DOTA-Peptide in 100 μl of HEPES buffer (PH=4) and the mixture allowed to react for 30 min at 80°C.

**Result:** [68G-DOTA-Peptide] was analyzed on Radio-HPLC; the ration time of the product was 8.4 min and Radio-TLC checks for the collected product confirm the high radiochemical purity.

**Discussions:** The labeled peptide is produce in good radiochemical yields and high radiochemical purity. Initial biological works are ongoing to evaluate its imaging potential mainly on a melanoma cell line. The findings will be presented.

### PP41 Is validation of radiochemical purity analysis in a public hospital in a developing country possible?

#### N Mambilima^1,2^, SM Rubow^1,2^

##### ^1^University Teaching Hospital, Lusaka, Zambia; ^2^Stellenbosch University, Stellenbosch, South Africa

###### **Correspondence:** SM Rubow **–** University Teaching Hospital, Lusaka, Zambia

**Introduction:** Pharmacopoeial or manufacturer’s radiochemical purity (RCP) analysis methods are not always practical in a hospital setting, leading to modifications or substitution with quicker, simplified, or cost effective analytical procedures. This study aimed to determine whether appropriate validation procedures based on ICH Q2A and Q2B guidelines are feasible in a resource limited environment, such as most hospital radiopharmacy settings in Southern Africa.

**Materials and methods:** Alternative RCP test methods for Tc-99m sestamibi involving the use of Whatman 31 ET (Wh) and Schleicher and Schuell (SS) chromatography paper were used. Locally procured Macherey-Nagel (MN) aluminium oxide TLC strips were intended as control method, as strips described in the manufacturer’s instructions were unavailable. All analyses were performed on the 3 different strips in parallel, and in triplicate. In-house prepared Tc-99m sestamibi, colloid and pertechnetate were assumed to be 100% pure. Samples containing mixtures of varying concentrations of the radiochemical components were analysed. Results from tests performed by different operators were used to judge intermediate precision, and time delay between spotting and developing was used to evaluate robustness**.**

**Results:** RCP of sestamibi without added impurities was 99.8%±0.0% MN, 99.5%±0.1% for Wh and 99.3%±0.2% for SS strips. Addition of pertechnetate and colloid to Tc-99m Sestamibi after completion of kit reconstitution, resulted in values for sestamibi higher than the calculated RCP for all 3 methods. The radiochromatogram scanner’s limit of detection and limit of quantitation were determined.

**Discussion and conclusion:** Due to the unexpected high RCP values after addition of impurities to sestamibi, all the analytical methods lacked specificity and accuracy. The MN test method showed exceptionally high values for sestamibi due to co-elution of the free pertechnetate with sestamibi. The MN RCP test method could therefore not be used as a reference standard. All three methods met the acceptance criteria for repeatability, intermediate precision, and robustness. Validating an analytical procedure in a hospital setting is only possible once some important prerequisites are met, such as availability of specified materials for the reference procedure, reliable reference standards, and availability of HPLC for radiopharmaceuticals that have impurities other than pertechnetate and colloid. A template validation protocol for TLC and paper chromatography was developed.

### PP42 Improved automated radiosynthesis of [18F]FEPPA

#### N. Berroterán-Infante^1,2^, M. Hacker^1^, M. Mitterhauser^1,3^, W. Wadsak^1,2^

##### ^1^Division of Nuclear Medicine, Department of Biomedical Imaging and Image-Guided Therapy, Medical University of Vienna, Vienna, Austria; ^2^Department of Inorganic Chemistry, University of Vienna, Vienna, Austria; ^3^LBI for Applied Diagnostics, Vienna, Austria

###### **Correspondence:** N. Berroterán-Infante **–** Division of Nuclear Medicine, Department of Biomedical Imaging and Image-Guided Therapy, Medical University of Vienna, Vienna, Austria

**Introduction:** The translocator protein (TSPO) has been proposed as a biomarker for several conditions, including neurodegenerative disorders, cancer and non-alcoholic fatty liver disease. Hence, a number of PET ligands have been developed to image the TSPO, including the aryloxyacetanilide derivative [^18^F]FEPPA, which is currently in clinical use [1]. The synthesis of [^18^F]FEPPA was first reported by Wilson et al. [2] and successively fully automated [3]. Nevertheless the purification of the crude product by means of semipreparative HPLC is unsatisfactorily time consuming (retention time t_R_ ≈ 23 min). Therefore, the aim of this study was to establish a new set of conditions, which can reduce the retention time of the product and consequently reduce the total time of the synthesis.

**Methods:** [^18^F]FEPPA was synthesized as described elsewhere [3] with minor modifications in a Nuclear Interface synthesizer (GE Healthcare, Sweden). Briefly, the tosylate precursor dissolved in acetonitrile was added to the azeotropically dried [^18^F]fluoride and the reactor was heated at 90° C for 10 minutes; after cooling, the reaction was quenched with a solution 50:50 acetate buffer:acetonitrile (pH=3,6) and automatically injected onto a semipreparative HPLC Column (Merck Chromolith® RP-18e, 100×10 mm). The product fraction was collected in water and the new aqueous solution was passed through a pre-conditioned C-18 plus SepPak cartridge. Finally, the product was eluted with ethanol and formulated with saline and phosphate buffer.

**Results:** The new procedure yielded 3.2 ± 0.3 GBq of [18F]FEPPA (27% ± 3%, not corrected for decay) within 17 min after the azeotropic drying of the [18F]fluoride. Specific activity ranged from 430 to 600 GBq/μmol. Radiochemical purity exceeded 98%.

**Conclusions:** A fast and reliable procedure for the production of [18F]FEPPA was successfully implemented, drastically reducing the total time of the radiosynthesis compared to the previous published method (17 min compared to 36 min).

### PP43 Synthesis and initial evaluation of Al18F-RESCA1-TATE for somatostatin receptor imaging with PET

#### Uta Funke^1^, Frederik Cleeren^1^, Joan Lecina^1^, Rodrigo Gallardo^2^, Alfons M. Verbruggen^1^, Guy Bormans^1^

##### ^1^Laboratory for Radiopharmacy, Department of Pharmaceutical and Pharmacological Sciences, KU Leuven, Leuven, Belgium; ^2^VIB Switch Laboratory, KU Leuven, Leuven, Belgium

###### **Correspondence:** Uta Funke – Laboratory for Radiopharmacy, Department of Pharmaceutical and Pharmacological Sciences, KU Leuven, Leuven, Belgium

**Introduction:** Indium-111 and gallium-68 labelled octreotide derivatives [1] are widely used in SPECT and PET imaging of somatostatin receptor expressing tumours and pancreatic dysfunction. In perspective of advantages of fluorine-18 (favourable half-life, high production capacity, low β-energy), more recently, derivatives based on [^18^F]aluminium fluoride complexation have been developed.[2] To achieve high labelling efficiency at biomolecule compatible conditions and improve radiotracer properties *in vivo*, we synthesized an octreotate conjugate with an acyclic *trans*-cyclohexane spanned pentadentate chelator: Restrained complexing agent 1, RESCA1 (**1**). After low-temperature Al^18^F-complexation, *in vitro* and *in vivo* characteristics of the new radiotracer were studied.

**Materials and methods:** Radiolabelling was achieved manually starting from 50-860 MBq of [^18^F]sodium fluoride. Al^18^F-RESCA1-TATE ([Al^18^F]**2)** was purified by SPE and analysed with iTLC, LC-MS and RP-HPLC. Stability of the radiotracer was investigated *in vitro* over a period of 3 h in EtOH/Saline 1:9 (RT), PBS (37 °C) and rat plasma (37 °C). *Ex vivo* biodistribution data were acquired at 10 and 60 min p.i. of 1.8 MBq [Al^18^F]**2** in healthy male mice (n=4 per time point), and compared with those of ^68^Ga-DOTA-TATE.

**Results:** Complexation of {Al^18^F}^2+^ with **1** succeeded with 54-77% of product formation within 15 min at room temperature and with 85-96 % after 12 min at 40 °C. 28-298 MBq of [Al^18^F]**2** were obtained with 94-98% RCP after SPE. After 3 h of incubation, the content of parent compound was 99% in formulation, 95% in PBS and 91% in plasma. Distribution of [Al^18^F]**2** in mice showed negligible bone uptake, but in comparison with ^68^Ga-DOTA-TATE less binding in endocrine tissues, 4 times higher uptake in kidneys, and lower excretion via the urinary pathway: 33.2%ID at 10 min and 62.2%ID at 60 min p.i,, compared to 44.1%ID at 10 min and 77.9%ID at 60 min. p.i., respectively.

**Discussion/conclusion:** RESCA1-TATE easily forms a chemically and biologically stable complex with {Al^18^F}^2+^. The radiotracer, however, showed lower uptake in organs known for high somatostatine expression in comparison with the clinical standard. This could be caused either by *in vivo* binding competition with non-labelled RESCA1 conjugate, accelerated clearance or lower binding affinity of [Al^18^F]**2**. Future experiments with HPLC purified radiotracer in competition binding studies *in vitro* and *in vivo*, and investigation in a high somatostatin receptor expressing tumour mouse model will have to clarify, whether specific activity, receptor affinity and/or *in vivo* kinetics of [Al^18^F]**2** can explain these results.

**Research support:** This research has received support within the SBO project MIRIAD, funded from the IWT Flanders.

### PP44 Radiolabeling and SPECT/CT imaging of different polymer-decorated zein nanoparticles for oral administration

#### Rocío Ramos-Membrive^1^, Ana Brotons^2^, Gemma Quincoces^1^, Laura Inchaurraga^2^, Inés Luis de Redín^2^, Verónica Morán^3^, Berta García-García^3^, Juan Manuel Irache^2^, Iván Peñuelas^1^

##### ^1^Radiopharmacy Unit, Department of Nuclear Medicine, University Clinic of Navarra, Pamplona, Spain; ^2^Department of Pharmaceutics and Pharmaceutical Technology, University of Navarra, Pamplona, Spain; ^3^Department of Nuclear Medicine, University Clinic of Navarra, Pamplona, Spain

###### **Correspondence:** Rocío Ramos-Membrive **–** Radiopharmacy Unit, Department of Nuclear Medicine, University Clinic of Navarra, Pamplona, Spain

**Introduction and aims:** Zein is the major storage protein of maize, with a “GRAS” status, that can be easily processed to form nanoparticles. Due to its amphiphilic character, mucoadhesive properties and a relatively high resistance to the effect of digestive enzymes, the resulting nanocarriers offer great potential for oral drug delivery purposes.The aim is optimization of ^99m^Tc radiolabelling of different kind of zein nanoparticles (ZNP) and the study by molecular imaging of their biodistribution in Wistar rats after oral gavage. The evaluated nanoparticles were bare zein nanoparticles (ZPN) and decorated with 3 different slippery polymers (ZNP-A, ZNP-B and ZNP-C).

**Materials and methods:** All nanoparticles were prepared by a desolvation technique. For this purpose, zein were first dissolved in an ethanol:water solution, obtained by the addition of an aqueous solution and spray-dried. NP were pre-tinned with SnCl_2_ and labelled with ^99m^TcO_4_^-^. Different SnCl_2_ concentrations ranging from 0.005 to 1.0 mg/mL were used for radiolabelling optimization. RCP was analysed by radio-TLC. For biodistribution studies 4 MBq (0.33mg NP) of ^99m^Tc-nanoparticles were used per animal (n=12). SPECT/CT studies were performed 1,2,4,6 and 8h post-administration. SPECT acquisition protocol was set to account for radionuclide decay. For quantitative analysis data sets were exported to PMOD software and VOIs drawn over CT images on stomach and intestine, count ratios calculated in respect to total animal counts and maximum counts normalized to the last image (8h) for each nanoparticle type.

**Resuts:** Radiochemical purity was >95% for ZNP-A and NPZ using [SnCl_2_]=0.5 mg/mL, while 1.0 mg/mL was needed for ZNP-B and ZNP-C to get similar results. SPECT/CT images showed that unmodified NPZ had a biodistribution in stomach and intestine almost constant during the first 4 hours. ^99m^Tc-NPZ-B had a higher stomach mucoadhesion while ^99m^Tc-NPZ-A showed a similar behaviour but with faster gastric drainage and ^99m^Tc-NPZ-C quickly moved to intestine. All these results were confirmed by the ratios obtained from PMOD processed images.

**Conclusions:** All Zein nanoparticles could be easily radiolabelled with 99mTc with >95% RCP. The results show a clear relationship between the biodistribution and the superficial properties which the coating material gives to the nanoparticle.

### PP45 An analysis of the quality of 68Ga-DOTANOC radiolabelling over a 3 year period

#### Trabelsi, M., Cooper M.S.

##### Radiopharmacy Department, Royal Liverpool University Hospital, Prescot Street, Liverpool, L7 8XP, UK

###### **Correspondence:** Cooper M.S. **–** Radiopharmacy Department, Royal Liverpool University Hospital, Prescot Street, Liverpool, L7 8XP, UK

**Introduction:** 68Ga-DOTANOC is an excellent radiopharmaceutical for imaging neuroendocrine tumours and gives superior quality images to equivalent peptides labelled with 111In. For that reason, we have been developing our 68Ga-DOTANOC radiolabelling service over the past 3 years. It is essential that we provide a high quality product and that the production is robust, reproducible and in line with GMP. Therefore, we have reviewed our quality data from the production of 68Ga-DOTANOC

**Materials and methods:** 68Ga-DOTANOC has been prepared using an iTG 68Ge/68Ga generator and iQS fluidic labelling module. DOTANOC (ABX) 50-100 μg was dissolved in 0.25M sodium acetate and heated for 5 min at 110°C before elution of the generator in 4mL 0.05M HCl. The reaction was allowed to proceed for 10 min before purification over a Sep-Pak C8 Plus cartridge eluted with 50% ethanol and washed with 0.9% NaCl. The generator was flushed each day with 5ml 0.05M HCl when not used for radiolabelling and with 20ml 0.05M HCl on Monday mornings. The 68Ga-DOTANOC product was tested for free 68Ga and colloids by TLC and analysed by HPLC, pH was measured, endotoxin content assessed and radioactivity, volume and the presence of particles were checked prior to release of the product. 68Ge content and sterility were assessed retrospectively.

**Results:** A total of 746 elutions were carried out between May 2013 and December 2015, the average elution yield was 85.5%. The average production yield (decay and generator yield corrected) was 66.5%. The average 68Ge breakthrough was 0.0028% for the 68Ga elution but 68Ge was not seen in the 68Ga-DOTANOC product. 296 production batches were made with 7 failures (2.4%) and these could all be put down to human error (leaks in the fluidic path, incorrect purification method etc.) and so have been minimised with training. No product has failed quality control tests for any reason. 499 patients have been imaged with the average number of patient’s per production run being 1.7.

**Discussion/conclusion:** The preparation of 68Ga-DOTANOC is a very robust process giving a high quality product in a very reliable manner. In spite of high 68Ge content in the 68Ga elution with the iTG generator, the product has negligible 68Ge content. The product has very low levels of 68Ga colloids and negligible amounts of free 68Ga and gives a very clean HPLC trace. We conclude that this method of production gives a safe, high quality product.

### PP46 In vivo biodistribution of adult human mesenchymal stem cells I (MSCS-ah) labeled with 99MTC-HMPAO administered via intravenous and intra-articular in animal model. Preliminary results

#### Alejandra Abella^1^, Teodomiro Fuente^1^, Antonio Jesús Montellano^2^, Teresa Martínez^1^, Ruben Rabadan^4^, Luis Meseguer-Olmo^3,4^

##### ^1^Unidad de Radiofarmacia Hospital Clínico Universitario Virgen de la Arrixaca, Madrid, Spain; ^2^Servicio de Medicina Nuclear, Clínica Belén, Murcia, Spain; ^3^Servicio de Traumatología, Hospital Clínico Universitario Virgen de la Arrixaca, Murcia, Spain; ^4^UCAM Universidad Católica San Antonio de Murcia, Murcia, Spain

###### **Correspondence:** Teresa Martínez **–** Unidad de Radiofarmacia Hospital Clínico Universitario Virgen de la Arrixaca, Madrid, Spain

**Introduction:** The application of adult human mesenchymal stem cells from bone marrow (MSCs-ah) is being considered a promising treatment of musculoeskeletal injuries, however, their trafficking and homing are still controversial issues with further studies needed. Our work is focused on in vivo biodistribution of 99mTc-HMPAO labelled MSCs-ah after both intravenous and intra-articular injection in an animal model.

**Materials and methods:** 2 x 106 MSCs-ah isolated from bone marrow of healthy subjects using SEPAX system were suspended in 1 ml of PBS and labeled with up to 9.3 Bq/cel of 99mTc-HMPAO. Radiolabeled cell suspensions were administered to 8 adult male New Zealand rabbits (3.5-4 k) randomized in two groups. In group A (n=4), cell suspensions were administered via intravenous (marginal ear vein) and in group B (n=4) by intra-articular injection. After dynamic acquisition of images every 30 s for 25 min, whole body static images in anterior projection at 1,6 and 24 hours were acquired, previous sedation. No immunosuppressive agents were administered.

**Results:** Early immune response to xenogenic MSCs-ah was not detected during the study. Neither adverse reaction nor technical complications were registered, so any and every animal was recruited for the study. In group A, high retention of the activity was observed in lung parenchyma, with kidney and bladder visualization from the start, and biliary and abdominal uptake from 1h, maintaining the pattern of distribution at 24h, with no significant activity in liver and spleen. Group B show activity in heart, spleen, liver, kidneys and bladder from the start to the late acquisition of 24h, with biliary and intestinal uptake from 6 h. No activity was observed in the lung parenchyma in this group.

**Discussion/conclusion:** Exogenous MSCs-ah administered via intravenous are trapped mainly in lungs, with slight posterior retrafficking to other organs. On the other hand, no lung trapping is observed after intra-articular injection, pointing out the suitability of this route of administration for the therapeutic potencial of MSCs-ah in musculoskeletal pathology**.**

### PP47 Synthesis of [^18^F]F-exendin-4 with high specific activity

#### Lehtiniemi P^1^, Yim C^2^, Mikkola K^1^, Nuutila P^1^, Solin O^1,2^

##### ^1^Turku PET Centre, University of Turku, Kiinamyllynkatu 4-8, FI-20500 Turku, Finland; ^2^Turku PET Centre, Åbo Akademi University, Porthansgatan 3, FI-20500 Turku, Finland

###### **Correspondence:** Lehtiniemi P – Turku PET Centre, University of Turku, Kiinamyllynkatu 4-8, FI-20500 Turku, Finland

**Introduction:** A reliable method for non-invasive in vivo quantification of beta-cell mass in human pancreas is needed to understand the pathophysiology of type 1 and 2 diabetes. Based on animal studies, the [18F]F-exendin-4-peptide looks promising for imaging pancreatic beta cells but further development of the tracer synthesis is needed to obtain high specific activity (SA) and synthesis procedures amendable for GMP production. A proof-of-concept study in type 1 diabetic patients and healthy subjects showed a correlation between beta cell mass and uptake of 111In-exendin-4, which could lead to further insight into the pathophysiology of diabetes. Recent results with [18F]F-exendin-4 analogues demonstrate enhanced pharmacokinetic properties, as compared to radiometal-labelled counterparts. Here we describe a synthesis of [18F]F-exendin-4 aiming at high SA for the product

**Materials and methods:** We have investigated several conditions and solvents for synthesizing [18F]F-exendin-4. Alkyne tosylate and a [18F]fluoride-Kryptofix complex were allowed to react in DMSO for 5 min at 80 °C. 18F-labelled tosylate precursor ([18F]FP) was isolated by semi-preparative HPLC and concentrated with a HLB cartridge, followed by elution with THF from the cartridge. Exendin-4-azide was allowed to react with [18F]FP in presence of CuSO4/Na-ascorbate for 5 min at room temperature using vigorous mixing. [18F]F-exendin-4 was isolated by semi-preparative HPLC and concentrated with a Sep-Pak C8 cartridge. [18F]F-exendin-4 was eluted with ethanol and PBS. Radio-TLC and radio-HPLC was used for radiochemical analysis and SA determination of the [18F]FP and [18F]F-exendin-4.

**Results:** 18F-labelling of [18F]FP proceeded in an 50% radiochemical yield (RCY). Isolation of [18F]FP by HPLC was efficient and the radiochemical purity was > 99%. The RCY of [18F]F-exendin-4 ranged between 25-60% (calculated from [18F]FP, uncorrected) and the radiochemical purity was > 96%. The SA was up to 350 GBq/μmol at EOS.

**Discussion/conclusion:** We now can produce [^18^F]F-exendin-4 with a SA of 350 GBq/μmol for synthesis batches of >500 MBq. Our further aim is to automate all synthesis procedures in order to have a GMP-level production of the tracer, suitable for clinical evaluation of [^18^F]F-exendin-4.

### PP48 Experimental radionuclide therapy with 177Lu-labelled cyclic minigastrin and human dosimetry estimations

#### von Guggenberg E^1^, Rangger C^1^, Mair C^1^, Balogh L^3^, Pöstényi Z^3^, Pawlak D^2^, Mikołajczak R^2^

##### ^1^Department of Nuclear Medicine, Innsbruck Medical University, Innsbruck, Austria; ^2^Radioisotope Centre POLATOM, National Centre for Nuclear Research, Otwock, Poland; ^3^”Frédéric Joliot-Curie” National Research Institute for Radiobiology and Radiohygiene (NRIRR), Budapest, Hungary

###### **Correspondence:** Rangger C **–** Department of Nuclear Medicine, Innsbruck Medical University, Innsbruck, Austria

**Introduction:** Radiolabelled cyclic minigastrin-analogues are promising candidates for possible clinical application in diagnosis and therapy of cholecystokinin-2 receptor (CCK2R) expressing tumours, such as medullary thyroid cancer (MTC) and SCLC. Within an international collaboration of the IAEA we performed biodistribution studies and an experimental radionuclide therapy in a CCK2R-specific mouse tumour model using two 177Lu-labelled cyclic minigastrin-analogues: DOTA-cyclo[γ-D-Glu-Ala-Tyr-D-Lys]-Trp-Met-Asp-Phe-NH2 (DOTA-cyclo-MG1) and DOTA-cyclo[γ-D-Glu-Ala-Tyr-D-Lys]-Trp-Nle-Asp-Phe-NH2 (DOTA-cyclo-MG2). Based on results of biodistribution studies dosimetric considerations for humans were extrapolated to evaluate the possible injected activity in first applications in patients.

**Materials and methods:** For biodistribution studies BALB/c nude mice were xenografted with A431 human epidermoid carcinoma cells transfected with the human CCK2R (A431-CCK2R) and transfected with the empty vector alone (A431-mock) in both flanks. Tumours were allowed to grow for 2 weeks. Biodistribution studies with 177Lu-DOTA-cyclo-MG1 and 177Lu-DOTA-cyclo-MG2 were performed at an injected dose of 1 MBq (0.02 nmol DOTA-peptide) at 30min, 4h, 24h, 48h, 72h and 168h p.i. (5 animals/group). Dose extrapolation to humans was based on linear scaling of injected activity per gram tissue between animals and humans and computed using OLINDA software. For experimental radionuclide therapy the animals were injected with both 177Lu-labelled DOTA-peptides at two different dose levels of 15 and 30 MBq corresponding to 0.6 and 1.2 nmol peptide (6 animals/group). For up to five weeks after treatment tumour volume and body weight was evaluated in comparison with a control group injected with physiological saline.

**Results and conclusion:** Biodistribution studies in tumour-bearing mice revealed an uptake of >3.5% ID/g for both 177Lu-labelled DOTA-peptides in the A431-CCK2R-tumour 30min p.i.. This uptake remained stable for 4h p.i. and declined to >2% ID/g at 24h p.i.. A much lower uptake was observed in the A431-mock tumour. The tumour-to-kidney ratio resulted to be in the order of 3. In the experimental radionuclide therapy a clear therapeutic effect could be observed. In comparison to the control group the mean tumour volume doubling time of A431-CCK2R-tumours was increased by a factor of 1.8 and 2.6 in the 15 and 30 MBq group, respectively. For the A431-mock-tumours lower factors of 1.2 and 1.7 were observed in both treatment groups proving a receptor-specific effect. On the basis of dosimetric extrapolation to humans first therapeutic applications in patients should start with 4 repeated administrations of 3-5 GBq to limit the cumulative kidney dose to <27Gy. Based on patient individual dosimetry accompanying the first therapy the injected activity may be adapted accordingly.

### PP49 Synthesis of radiopharmaceuticals for cell radiolabelling using anion exchange column

#### Socan A^1^, Kolenc Peitl P^1^, Krošelj M^1^, Rangger C^2^, Decristoforo C^2^

##### ^1^Department of Nuclear Medicine, University Medical Centre Ljubljana, Ljubljana, Slovenia; ^2^University Clinic for Nuclear Medicine, University for Medicine, Innsbruck, Austria

###### **Correspondence:** Socan A – Department of Nuclear Medicine, University Medical Centre Ljubljana, Ljubljana, Slovenia

**Introduction:** 111In labelled radiopharmaceuticals are extensively used for cell labelling in routine clinical practice. Being members of coordinating radiometals group 68Ga and 89Zr are suitable candidates for synthesis of radiopharmaceuticals used for cell labelling. Due to its half life (78,4h) 89Zr labelled radiopharmaceuticals, could possibly enable long-term “in vivo” cell tracking with several attractive clinical applications (imaging of infection and inflammation, stem cell therapy). Aim of study was to develop a novel, versatile approach to prepare tracers (oxine, tropolone) using concentration on an anion exchange column.

**Materials and methods:** The column (SepPAK QME) was washed with 2 ml of water. Samples of 68Ga, 111In, 89Zr-HEPES (0,5M, 1M) solutions, samples varying in pH (3-9) and volume were prepared and applied on column. Activity retained on, activity washed from column and pH of the solution were measured. Samples of 0.3 ml ligand solution (tropolone:1mg/ml, water; oxine:1mg/ml, 20%, 50% EtOH) were applied on the column and incubated. The column content was washed with 2 ml of PBS. Activity and pH measurements were performed as described above. Complex formations were analyzed using extraction method into octanol.

**Results:** With 68Ga-HEPES (1M) yield of activity retained on the column was above 89% and was not pH (3.7-7.3) and volume dependent (0.5-2.5ml). Yield of 68Ga-oxine solution (20%EtOH) was between 43-77% with pH between 6.1-7.4 and extraction into octanol above 96%. Yield of activity retained on column with 111In-HEPES (1M) varied 31–98% and was pH and volume dependent. Yield decreased with volumes applied greater than 0.5 ml and pH bellow 4. Yield of 111In-tropolone solution was between 41 - 61% with pH between 6-6.7 and extraction into octanol above 82%. Yield of 111In-oxine solution (20%EtOH) was between 41-96% and extraction into octanol above 92%. 89Zr-HEPES (0.5M) yield of activity retained on column was above 96% and was not pH (5.5-9) and volume dependent (0.3-1.5ml). Yield of 89Zr-tropolone solution was above 85% with pH between 7-7.5 and extraction into octanol above 94%. Yield of 89Zr-oxine solution (50%EtOH) was between 25-71% with pH between 7-9 and extraction into octanol above 86%.

**Conclusion**: Concentration of coordinating radiometals 111In, 68Ga and 89Zr on an anion exchange column in HEPES allows on-column formation of tracers (oxine, tropolone) in very small volumes suitable for cell labelling with good yields and quality. This is a versatile and promising tool to prepare tracers on automated systems further used for cell labelling and potentially suitable for clinical practice.

### PP50 [68Ga]peptide production on commercial synthesiser mAIO

#### Collet C.^1,2^, Remy S.^1,2^, Didier R^1,2^,Vergote T.^4^,Karcher G.^1,2,3^, Véran N.^1,3^

##### ^1^Nancyclotep, Plateforme d’imagerie moléculaire, 54500 Vandoeuvre les Nancy, France; ^2^Université de Lorraine, F-54500 Vandoeuvre les Nancy, France; ^3^CHRU de Nancy-Brabois, F-54511 Vandoeuvre les Nancy, France; ^4^Trasis SA, B-4430 Ans, Belgium

###### **Correspondence:** Collet C. **–** Nancyclotep, Plateforme d’imagerie moléculaire, 54500 Vandoeuvre les Nancy, France

**Introduction:** Gallium-68 is a metallic positron emitter with a half-life of 68 min that is ideal for labelling small peptides as radiopharmaceuticals thanks to the use of a chelating agent with several clinical applications. Numerous gallium-68 labelled peptides (eg. [68Ga]DOTA-TOC/-NOC, [68Ga]HBED-PSMA-11, [68Ga]NODAGA-RGD) have shown their interest [1,2]. Developing an easy, rapid and performant labelling method is important. Different methods for the pre-purification of the generator eluate have been explored in the literature, although recent improvement on some generator brands (i.e. low 68Ge breakthrough and low metallic impurities content), makes this pre-purification unnecessary. Development of a labelling process, GMP-compatible and reproducible, using a commercial synthesis module for every peptide labelling is a real challenge for the nuclear medicine. The method presented herein uses a cassette-based approach and a MiniAIO (mAIO, Trasis®) module and has been tested with the IGG100 68Ge/68Ga generators.

**Materials and methods** Preclinical IGG100 was used as ^68^Ge/^68^Ga generator. Precursors of radiolabelling were bought from ABX. Automated ^68^Ga-labelling was performed without pre-purification in mAIO module. Reaction parameters such as sodium acetate concentration, precursor quantity, temperature and time were optimized for each peptide. Labelling efficiency was determined on Waters HPLC system.

**Results:** DOTANOC, DOTATOC, HBED-PSMA-11 and NODAGA-RGD were tested for ^68^Ga-labelling without pre-purification. Optimal and reproducible conditions were determined for each peptide. ^68^Ga-peptides were synthesised with excellent incorporation yields, (90-99%) and high synthesis yields > 60% in less than 15 min.

**Discussion/conclusion:** We developed an efficient automated strategy for peptide labelling with gallium-68. [^68^Ga]DOTANOC, DOTATOC, [^68^Ga]HBED-PSMA-11, [^68^Ga]NODAGA-RGD were obtained in high radiochemical yield. Their preparation could be performed with this automation and their use in human could be done under clinical trial.

### PP51 Dry kit formulation for efficient radiolabeling of 68Ga-PSMA

#### D. Pawlak, M. Maurin, P. Garnuszek, U. Karczmarczyk, R. Mikołajczak

##### National Centre for Nuclear Research Radioisotope Centre POLATOM, Sołtana 7, 05-400 Otwock, Poland

###### **Correspondence:** M. Maurin **–** National Centre for Nuclear Research Radioisotope Centre POLATOM, Sołtana 7, 05-400 Otwock, Poland

**Introduction:** The development of dry kits containing the active ingredient and suitable excipients permits more efficient use of 68Ge/68Ga radionuclide generators, despite the limitations in the variety of generator eluent’s composition and pH. Our aim was to develop a dry kit formulation for 68Ga radiolabeling of the PSMA inhibitor, PSMA-11 (Glu-CO-Lys(Ahx)-HBED-CC).

**Methods**: A series of experiments on “wet” labelling in the presence of various amounts of sodium acetate and ascorbic acid were performed taking into consideration the challenge to find the composition of buffering agents ready to maintain pH value between 4 and 5 after addition of various volumes of 68Ga eluate (volumes of 1-4mL 0.1M HCl). For quality control of the 68Ga–PSMA-11 the RP-RadioHPLC (Kinetex C18 150mm; A: 0.1%TFA/H2O, B: 0.1%TFA/CAN) was used with gradient conditions (according to M. Eder et al. Pharmaceuticals 2014, 7, 779-796) as well as in isocratic elution (optimally set to 17% B) which visualized the radiolabelled and non-labelled PSMA-11 in UV chromatogram. The radiochemical purity also was tested by TLC (ITLC SG; 1: 0.9%NaCl/MeOH 80/20 v/v; 2: 0.9%NaCl/MeOH/25%NH3, 80/20/5 v/v/v; 3: 1M NH4OAc/MeOH 50/50 v/v).

**Results and discussion:** Ascorbic acid (25mg/mL) added to the “wet” labelling mixture increased the radiolabeling yield (98.8%) compared to the labelling performed in acetate buffer only (95.0%). It also increased the buffering capacity of the formulation. These results were further used to formulate the dry kit composition using 30μg of PSMA-11, 60mg of sodium acetate and 12,5mg of ascorbic acid. The yield of 68Ga labelling of PSMA-11 from the freeze-dried kit was > 95%. The biodistribution of such prepared 68Ga-PSMA-11 was investigated in healthy Balb/c mice (n=5) after intravenous injection of 0.1mL (38 MBq/mL) (at 15, 30, 60 and 120min). 68Ga-PSMA-11 was rapidly cleared from the blood and excreted by kidney route with >70% urine elimination at 1h p.i. These results confirmed high stability of the 68Ga-PSMA-11 complex in vivo, with low contribution of free gallium-68, which circulates longer in blood stream. It is worth noting that the addition of ascorbic acid changes the biodistribution of free gallium and favors its blood clearance. This is probably due to the formation of week Ga complex with ascorbic acid which blocks Ga access to ferritin, hence resulting in lower liver and spleen accumulation.

### PP52 Development of an experimental method using Cs-131 to evaluate radiobiological effects of internalized Auger-electron emitters

#### Pil Fredericia^1^, Gregory Severin^1^, Torsten Groesser^1^, Ulli Köster^2^, Mikael Jensen^1^

##### ^1^Hevesy Laboratory, DTU-Nutech, Technical University of Denmark, Roskilde, Denmark; ^2^Institut Laue-Langevin, Grenoble, France

###### **Correspondence:** Mikael Jensen **–** Hevesy Laboratory, DTU-Nutech, Technical University of Denmark, Roskilde, Denmark

**Introduction:** The low energy electrons emitted in the Auger cascade of certain isotopes are expected to have a disproportionately high radiobiological effect. This is seen as the effect of the multiplicity and predominance of very low energy electrons in dose delivery. Despite many years of research in Auger emitters, only few direct measurements of the radiobiological effect of such Auger cascades have been published. One problem in making such fundamental dose-effect experiments is the method to bring Auger-emitting isotopes inside the cell and close to the nucleus in a predictable and quantifiable manner. Only when the location, activity, and time-activity profile are known it is possible to compare the observed damage with damage induced by the same dose and dose rate given by external irradiation. We have used the biological uptake of the Auger-emitting potassium analogue Cs-131 (9.7 d) into mammalian cells in culture as a test system for measuring the dose-effect of Auger electrons.

**Materials and methods:** Cs-131 was repeatedly extracted from Ba-131 (11.5 d) made by high flux neutron irradiation at ILL (6 to 10 days at (1.1-1.3) 1015 n.cm-2s-1) of either natural Ba (17 mg as carbonate) or 49% enriched Ba-130 (0.15 mg as nitrate). Such targets were dissolved in hydrochloric acid, and re-precipitated with ammonium carbonate. The supernatant was spiked with sodium hydroxide, then dried and fired, leaving cesium-131 as a chloride salt with NaCl carrier. The re-precipitated targets were stored for 1-2 weeks for buildup of Cs-131, and then the process can be repeated. We have milked up to 4 batches of useful Cs-131 activity from a single activated barium batch from ILL. The harvested, 131Cs/NaCl salt was dissolved in water to reach isotonicity, and was offered to V-79 and HeLa cells in normal growth media. The cells invariantly up-concentrated the cesium activity, reaching a plateau after 8-10 hours.

**Results:** Using enriched Ba-130, higher activity is obtained, but even with natural barium activities up to 80 MBq have been available repeatedly for the uptake experiments. Under optimal conditions, the cells in culture can reach intracellular activities over 1 Bq/cell during 4 hours incubation. The alkali-metal nature of cesium gives us reasons to assume a homogeneous intracellular distribution, allowing a first-principles calculation of the absorbed dose. With this method and unique isotope, we can compare the effect of equivalent absorbed doses of external gamma and internal, predominantly Auger delivered dose.

### PP53 Preclinical comparative evaluation of NOTA/NODAGA/DOTA CYCLO-RGD peptides labelled with Ga-68

#### R. Leonte^1,2^, F. D. Puicea^1,2^, A. Raicu^1^, E. A. Min^1^, R. Serban^1^, G. Manda^3^, D. Niculae^1^

##### ^1^Horia Hulubei National Institute for Physics and Nuclear Engineering, Radiopharmaceuticals Research Centre, Magurele, Romania; ^2^Politehnica University, Bucharest, Romania; ^3^National Institute of Patology Victor Babes, Bucharest, Romania

###### **Correspondence:** D. Niculae **–** Horia Hulubei National Institute for Physics and Nuclear Engineering, Radiopharmaceuticals Research Centre, Magurele, Romania

**Introduction:** The αvβ3 integrin receptors, expressed on tumor cell membranes can be preferentially targeted by peptides containing the RGD sequence. NOTA-SCN-Bn-E-[c(RGDyK)2], NODAGA-RGDfK, DOTA-E-[c(RGDfK)2], and DOTA-RGDfK were labelled with Ga-68 and tested for radiolabelling yield, purity, stability, in vitro binding, ex vivo biodistribution, and in vivo imaging.

**Materials and Methods:** The radiolabelling was performed using an automated system with inline quality control. Fraction of 68GaCl3 eluate from an organic matrix based 68Ge/68Ga generator, 400-700 MBq in 2 mL, was used to label NOTA/NODAGA/DOTA-derivatised cRGD peptides. Elution, labeling and purification procedures were performed in 20-25 min. The biodistribution was tested in tumor bearing animal models (U87MG, Walker 256, B21 and AR42J). The affinity of cyclo-RGD peptides to tumor cell-surface receptors was determined by real-time quantification using Ligand Tracer.

**Results:** Nanomoles of peptides were labeled with high yields. NOTA-SCN-Bn-E-[c(RGDyK)2] and NODAGA-cRGDfK was labelled at room temperature, while DOTA-cRGDfK, both monomer and dimer, were labeled at 95°C, with over 90% yield. The biodistribution pattern of 68Ga-NOTA/NODAGA/DOTA-cRGD peptides shows high tumor uptake, fast blood clearance, high tumor to background ratios and renal elimination. Up to 5.3% ID/g of 68Ga-DOTA-E-[c(RGDfK)2] was found on U87MG tumor; the biological behavior of the four tracers will be discussed in detail. Binding to receptors was achieved in the first 3 min of incubation of all tracers, however the retention 68Ga-DOTA-E-[c(RGDfK)2] on both AR42J and U87MG was the highest, up to 58% from activity in the cells.

**Discussion/conclusions**: The radiolabelling with Ga-68 of very promising candidates for imaging targets of interest in cancer diagnosis and therapy follow-up such as αvβ3 receptors, were successfully adapted on the automated module, reducing the reaction time and operator exposure. The preclinical biological evaluation of proposed tracers show that the uptake and retention on different tumor cells depends on tumor type, receptors expression and dimerization. Very good uptake-retention profile of 68Ga-DOTA-E-[c(RGDfK)2] makes it our option for further investigations as therapeutic agent. Research contracts CRP16500 IAEA and MEN UEFISCDI, PN II 228/2014 are acknowledged for financial support.

### PP54 Synthesizer- and Kit-based preparation of prostate cancer imaging agent 68Ga-RM2

#### Marion Zerna, Hanno Schieferstein, Andre Müller, Mathias Berndt

##### Piramal Imaging, Berlin, Germany

###### **Correspondence:** Mathias Berndt **–** Piramal Imaging, Berlin, Germany

**Introduction:** Prostate cancer (PCa) is the most common cancer in men and the second most common cause in cancer-related deaths. 68Ga-RM2 is a nona-peptide with optimized binding sequence for Gastrin Releasing Peptide receptor (GRPr) which is overexpressed in PCa.1 In clinical studies 68Ga-RM2 has demonstrated its ability for detection of primary prostate cancer with high specificity due to its low uptake in normal prostate and in benign hyperplasia.2 Recent studies showed further potential of 68Ga-RM2 for detection of recurrent PCa and for imaging of estrogen receptor positive breast cancer.3,4. To provide 68Ga-RM2 for further clinical research, robust methods for preparation of this promising tracer are needed. Herein we describe the results of automated synthesizer based 68Ga-radiolabelings of RM2 as well as of simplified “shake-and-bake” kit-preparations.

**Materials and methods:** The automated 68Ga-radiolabeling was optimized on a PharmTracer module (Eckert&Ziegler). The influence of several scavengers was investigated, as well as a processes with and without subsequent purification via C18 cartridge. Optimized conditions were used on further synthesizers (Scintomics, ITG). In a second approach, a simplified kit preparation was established. The 68Ga-generator eluate was added directly into a vial containing RM2-precursor, buffer and scavenger. The vial was heated and optionally buffer was added for pH-adjustment.

**Results:** Automated procedures on various synthesizers provided up to 850 MBq of 68Ga-RM2 in >70% uncorrected yield in 20 min including purification and formulation. The radiochemical purity was >97% (HPLC and TLC) if ascorbic acid was used as scavenger. The simple and easy to use kit preparation approach provided 68Ga-RM2 in 90% uncorrected yield and purity of >95%.

**Discussion/conclusion:** 68Ga-RM2 can be reliably obtained on automated synthesizers as well as by a simple “shake-and-bake” kit preparation strategy. Both methods provided 68Ga-RM2 in high yields and high radiochemical purity.

### PP55 Synthesis of pancreatic beta cell-specific [18F]fluoro-exendin-4 via strain-promoted aza-dibenzocyclooctyne/azide cycloaddition

#### Cheng-Bin Yim^1,2^, Kirsi Mikkola^1,2^, Pirjo Nuutila^1,2^, Olof Solin^1,2^

##### ^1^Turku PET Centre, University of Turku, Turku, Finland; ^2^Åbo Akademi University, Turku, Finland

###### **Correspondence:** Cheng-Bin Yim **–** Turku PET Centre, University of Turku and Åbo Akademi University, Turku, Finland

**Introduction**: Diabetes has dictated many lives, and its lifetime treatment has accumulated into one of the most costly medical conditions, and yet, its pathophysicology is still undisclosed. However, efforts to develop sensitive noninvasive methods to quantify beta cell mass, which underlies the development of diabetes, have been challenged by the low abundance (1-2 %) and high dispersion of beta cells in the pancreas [1]. Recent preclinical studies using 18F-labelled exendin-4 showed specific targeting of pancreatic islets and insulinomas with favourable clearance kinetics that would potentiate clinical utility [2]. The focus of the present study is the radiosynthesis of a novel 18F-labelled exendin-4 analogue with a clear clinical prospective for imaging pancreatic beta cell mass.

**Materials and methods**: The synthesis of [18F]fluoro-exendin-4 is depicted in Scheme 1. The dried [18F]fluoride-Kryptofix complex was reacted with tosylated prosthetic compound 1 in dimethylsulfoxide at 80 °C. After chromatographic isolation, the 18F-labelled azide 2 was allowed to react with cyclooctyne-derivatised exendin-4 in ethanol/water at 60 °C for 30 min. Isolation and radiochemical analysis of [18F]fluoro-exendin-4 was performed using high performance liquid chromatography (HPLC) with radiodetector.

**Results:** Preliminary results showed efficient reaction between prosthetic reagent [18F]2 and exendin-4 precursor with a radiochemical yield of 30 %. The absence of major radioactive by-products assured neat purification using HPLC. The average isolated yield of [18F]exendin-4 after HPLC purification was 180 MBq starting from 3.5 GBq aqueous [18F]fluoride.

**Discussion:** Currently, the scope of this reaction is being explored and its labelling conditions optimized. Preclinical studies with [18F]fluoro-exendin-4 will include in vivo stability, in vivo distribution kinetics, and dosimetry calculations in healthy and diabetic animal models.

### PP56 Automated systems for radiopharmacy

#### D. Seifert, J. Ráliš, O. Lebeda

##### Nuclear Physics Institute of the CAS, v. v. i., Husinec-Řež 130, 250 68 Řež, Czech Republic

###### **Correspondence:** O. Lebeda **–** Nuclear Physics Institute of the CAS, v. v. i., Husinec-Řež 130, 250 68 Řež, Czech Republic

**Introduction:** Automated modules for target processing and production of radiopharmaceuticals become to be a more and more required in both R&D and routine practice. However, it turns out that available systems for processing of targets for radiometal production and for labeling performed via microfluidic technology are still rather limited. Our aim was to develop a multipurpose automated systems for processing solid state targets and for operating microfluidic PMMA-based chip.

**Materials and methods:** *Stingray – Automated microfluidic system.* The device is a versatile automated system that allows for control of variety of processes performed on a microfluidic chip. The system makes possible operations like precise mixing, separation, extraction or small-scale nanoparticle formation. It consists of 3 independent inlets for 3 different mobile phases with an in-built degasser, 4 peek solenoid valves, 2 independent nano piston pumps, 2 selectors (6/7), 2 manual inject valves with 100 μl loop volume (adjustable), 2/10 valve, 3 check valves and unique automated manifold for inserting microfluidic chip. This platform offers enough variability of reaction parameters and allows for a repeated return of a sample to the microfluidic chip. It may enhance the yield dramatically. *CRAB* – *versatile platform for separation, formulation and simple labelling processes*. The main parts of the system are two reactors, two selectors, peristaltic pump, 3/2 way valves, and the column. Prime reactor R1 allows for transport solid target material from shielding container to process position and for handling liquid target content. It is leak-proof for 5 bars. There is an in-built solid phase extraction (SPE) column for separation processes driven by peristaltic pump and solvents. Four positions are available for uploading the solvents into the reactor R1 or on the SPE column. Splitting the separated radionuclide from the target matrix, including enriched material, is enabled thanks to the smart software checking the column output activity and controlling the splitting valves position. Final activity concentration can be precisely set via the case software without losses on the walls of reactor R2. There are 3 positions for uploading the solvents to the reactor R2 for formulation or for simple labelling steps like chelation.

**Results and conclusion:** Both automated platforms are versatile, reliable tools that offer user-friendly settings for wide range of processes. Stingray is operable up to 200 bar back pressure allowing thus the flow rate of 20 ml/min. Crab was tested on separation of 61Cu and 64Cu from Ni targets in 99% separation yield of copper radionuclides and almost quantitative recovery of target matrix.

### PP57 Simple, suitable for everyday routine use quality control method to assess radionuclidic purity of cyclotron-produced 99mTc

#### Svetlana V. Selivanova, Helena Senta, Éric Lavallée, Lyne Caouette, Éric Turcotte, Roger Lecomte

##### Molecular Imaging Centre, CRCHUS, Université de Sherbrooke, Sherbrooke, QC, Canada

###### **Correspondence:** Svetlana V. Selivanova **–** Molecular Imaging Centre, CRCHUS, Université de Sherbrooke, Sherbrooke, QC, Canada

**Introduction:** Formulations of cyclotron-produced 99mTc contain trace amounts of other Tc isotopes depending on the isotopic composition of starting 100Mo. Presence of other nuclides may affect image quality and patient dosimetry. Therefore, quantification of radioisotopic impurities is very important. Currently, radioisotopic purity of cyclotron-produced 99mTc is measured using gamma-ray spectrometry and is time-consuming. We sought to identify another approach better suitable for quality control during routine productions.

**Materials and methods:** 100Mo targets (99.815% enrichment) were irradiated in a cyclotron at 24 MeV for 2 h. The targets were processed and radioisotopic purity of resulted 99mTc was evaluated using three different procedures. Method 1, “Dilution”: An aliquot of diluted 99mTc solution was measured using gamma-ray spectrometry. Percentage of each radioactive contaminant was calculated dividing activity of each isotope by total activity in the sample. Method 2, “Shielding”: A vial containing formulated 99mTc was measured in a calibrated ionization chamber. Then, it was inserted into a 6 mm thick lead canister, used in nuclear medicine for 99Mo breakthrough determination, and measured using gamma-ray spectrometry. The activity of each radioactive contaminant was calculated and divided by total activity in the vial as measured in ionization chamber. Method 3, “High-energy breakthrough”: A vial containing formulated 99mTc was measured in a calibrated ionization chamber as is and inside the lead canister. The ratio of two measurements gave relative proportion of high-energy gamma-rays.

**Results:** There was a tendency to slightly overestimate the radioisotopic purity of cyclotron-produced 99mTc with “dilution” method (99.983±0.007%) compared to “shielding” method (99.979±0.009%). Since both methods require measurements by gamma-ray spectrometry, they are time-consuming, involve elaborate calculations, and demand specific expertise. “High-energy breakthrough” measurements showed good correlation with the results of gamma-spectrometry.

**Conclusions:** With “shielding” method, lead canister attenuated low-energy gamma-emission (including from 99mTc) and allowed detecting impurities present in trace amounts with better sensitivity. While “high-energy breakthrough” measured in ionization chamber does not give true radioactivity values for each contaminant, it offers sufficient information about relative amount of high-energy radioactive impurities in the product. This could serve as a surrogate test during routine productions when cross-calibrated with gamma-spectrometry data for given isotopic composition of 100Mo targets.

### PP58 Effective dose estimation using Monte Carlo simulation for patients undergoing radioiodine therapy

#### Marina Zdraveska Kochovska^1^, Emilija Janjevik Ivanovska^2^, Vesna Spasic Jokic^3^

##### ^1^Institute of pathophysiology and nuclear medicine, Medical faculty, Skopje, Macedonia; ^2^Faculty of Medical Sciences, University of “Goce Delcev”, Stip, Macedonia; ^3^Faculty of Technical Sciences, Universty of Novi Sad, Novi Sad, Serbia

###### **Correspondence:** Marina Zdraveska Kochovska – Institute of pathophysiology and nuclear medicine, Medical faculty, Skopje, Macedonia

**Introduction:** Therapeutic or diagnostic radiopharmaceutical capsule containing Na131I stays in stomach for about 15 minutes before the absorption starts, long enough to make possible risky exposure.

**Material and methods:** Investigations were performed at Nuclear medicine department, Medical faculty, Skopje. Eighty seven patients were reviewed, age between 21 and 73. Patients were divided in 20 groups receiving average dose per group 1813 MBq to 6105 MBq. For this purpose we used Monte Carlo 4b code for nominal activity of 6105 MBq. The new MIRD phantom model was used for calculation of doses absorbed in specific organs due to the presence of the source in some other organ. The stomach wall is represented by the volume between two concentric ellipsoids and the contents within the inner ellipsoid. The composition of each tissue type is given in ICRP reports 70 and 89 and ICRU 46.

**Results:** Calculated results are given for imparted energy per transformation dose equivalent and effective doses for different organs. The effective doses were between 9,17nSv for bone surface to 122.4 mSv for stomach. As the total dose is estimated to be 126.73 mSv it is obvious that the highest part is received by stomach.

**Discussion/conclusion:** Capsules containing Na131I are widely used as they are more comfortable for administration and there is less possibility for local contamination of patient and medical staff. During the time before absorption starts a large amount of radioactivity needlessly expose a part of stomach and several surrounding organs. Obtained results indicate that values of local doses in stomach wall could not be ignored. As it is not possible to measure these dose directly Monte Carlo calculation seems to be good solution for this problem.

### PP59 Chemical analysis of the rituximab radioimmunoconjugates in lyophilized formulations intended for oncological applications

#### Darinka Gjorgieva Ackova^1^, Katarina Smilkov^1^, Petre Makreski^2^, Trajče Stafilov^2^, Emilija Janevik-Ivanovska^1^

##### ^1^Department of Pharmacy, Faculty of Medical Sciences, University Goce Delčev – Štip, R. Macedonia; ^2^Department of Chemistry, Faculty of Natural Sciences and Mathematics, University “Ss. Cyril and Methodius”- Skopje, R. Macedonia

###### **Correspondence:** Darinka Gjorgieva Ackova **–** Department of Pharmacy, Faculty of Medical Sciences, University Goce Delčev – Štip, R. Macedonia

**Introduction:** For a protein based drug including antibody based radiopharmaceuticals, a structural characterization is mandatory before any possible start of a clinical trial. Vibrational spectroscopic techniques, as Fourier transform infrared (FTIR) spectroscopy and Raman spectroscopy are one of the biophysical methods for structural characterization of proteins because of their sensitivity to the composition and architecture of molecules. Here we used vibrational spectroscopy to characterize the physico-chemical stability and structural changes of three immunoconjugates of rituximab, intended for labeling with radioisotope of choice (^177^Lu, ^90^Y and/or ^68^Ga), relevant for the stability of therapeutic/diagnostic antibodies during preparation, storage and/or transport.

**Materials and methods:** Rituximab, conjugated with three different bifunctional chelating agents (BFCAs), p-SCN-Bn-DOTA, p-SCN-Bn-DTPA and 1B4M-DTPA in a form of lyophilized preparations non-radioactive labeled with above mentioned radioisotope analogues, was subjected to characterization and determination of secondary structure and quality parameters (purity, integrity, fragmentation and aggregation of the antibody) by FT-IR and Raman spectroscopy.

**Results:** Based on the frequencies assigned for amide I, II and III bands, the studied formulations contain highest percentage of β-sheet conformation (antiparallel and parallel) in the structure, followed by α-helices. Significant changes upon processes of conjugation and lyophilization were not observed in comparison with spectra of native antibody.Vibrational spectroscopic data allow detection of alterations in investigated protein models as well as rapid assessment of conformational changes resulting from ligand binding, aggregation or macromolecular interactions. Appearance of strong absorption bands below 1620 cm^–1^ can be correlated with aggregation-usually associated with the formation of new strong β-sheet structures. According to the obtained spectra, it is important that we observed retaining of native structure of the antibody and no obvious aggregation (the lowest band frequency detected was 1620 cm^–1^ with weak intensity) in all samples of lyophilizated conjugates.

**Conclusion:** We investigated the application of vibrational spectroscopy in assessment of conformational changes during stress conditions, as lyophilization and non-radioactive labeling are, using different rituximab-conjugates. The results are a good foundation for further radiolabeling studies of the lyophilized formulations for possible therapeutic application.

### PP61 The need and benefits of established radiopharmacy in developing African countries

#### Aschalew Alemu^1,3^, Joel Munene Muchira^2,3^, David Mwanza Wanjeh^2,3^, Emilija Janevik-Ivanovska^3^

##### ^1^Faculty of Medicine, Addis Ababa, Ethiopia; ^2^Ministry of Health, Nairobi City, Kenya; ^3^Faculty of Medical Sciences, University Goce Delčev – Štip, R. Macedonia

###### **Correspondence:** Aschalew Alemu **–** Faculty of Medicine, Addis Ababa, Ethiopia

**Introduction:** Our work is to present the current status, and in the same time the need and benefit of establishment of Radiopharmacy practice in Eastern Africa using the perspective of Kenya and Ethiopia. The exact information on the status and size of Radiopharmacy units, regionally, is still not clearly documented, as well human resources, education, suitable training and local demand for the Radiopharmacy and Nuclear Medicine services. The Radiopharmacy Practice requires well-defined and controlled conditions to avoid the risk contamination with microbes, pyrogens and particulate matter as well as cross contamination with other radiopharmaceuticals. Corresponding to the expected improvement, the principles of Good Practices in all levels should be planned, introduced by the planned priority and strictly observed in the production, preparation, testing and the packaging of the final product ready for use. Because non-communicable diseases (NCDs) are a challenge of epidemic proportion and that they will be the commonest cause of mortality in Africa by 2030, early detection and treatment can significantly improve patient outcomes. Radiopharmaceuticals should have become of invaluable benefit because they offer the most sensitive tools in the detection, diagnosis and targeted therapy of NCDs and also infectious diseases. In light of the foregoing, therefore, radiopharmacy has a huge role to play in responding to the unfolding new disease trends in sub-Saharan Africa. The preparation of radiopharmaceuticals for human use requires that it is carried out in well-defined and controlled conditions to avoid the risk contamination with microbes, pyrogens and particulate matter as well as cross contamination with other radiopharmaceuticals. Every procedure undertaken should be done according to the clearly defined protocol and under the right conditions so as to build quality into the product. Radiopharmacy professionals should have adequate training in all aspects of sterile production, quality control, GMP, GLP, radiation safety and radiochemistry to ensure that they are competent to handle radioactive materials and that they can take responsibility for their level of practice.

**Conclusions:** We are expecting that the good education and continuing training of all professionals working in Radiopharmacy will be the key point how to create the network of all professionals and state authorities for establishing and develop Good Radiopharmacy Practice, qualified personnel and appropriate regulation according to the local and international parameters will be step forward to have advanced health care system and confidence of the patients.

### PP62 University Master Program of Radiopharmacy – step forward for Good Radiopharmacy Education

#### Emilija Janevik-Ivanovska^1^, Zoran Zdravev^2^, Uday Bhonsle^3^, Osso Júnior João Alberto^3^, Adriano Duatti^4^, Bistra Angelovska^1^, Zdenka Stojanovska^1^, Zorica Arsova Sarafinovska^1^, Darko Bosnakovski^1^, Darinka Gorgieva-Ackova^1^, Katarina Smilkov^1^, Elena Drakalska^1^, Meera Venkatesh^3^, Rubin Gulaboski^1^

##### ^1^Faculty of Medical Sciences, Goce Delcev University, Stip, Republic of Macedonia; ^2^Faculty of Informatics, Goce Delcev University, Stip, Republic of Macedonia; ^3^Radioisotope Products and Radiation Technology Section, Division of Physical and Chemical Sciences, IAEA, Vienna, Austria; ^4^Laboratory of Radiopharmaceutical Chemistry, Department of Chemical and Pharmaceutical Sciences, University of Ferrara, Ferrara, Italy

###### **Correspondence:** Emilija Janevik-Ivanovska **–** Faculty of Medical Sciences, Goce Delcev University, Stip, Republic of Macedonia

**Purpose:** The importance of well-established and recognized education in Radiopharmacy became a need all around the world as result of different national regulation, different system of education and mostly of the level of need, development and investment in clinical and research usage of radiopharmaceuticals. The substantial confusion and challenge in the same time is to define the appropriate and sufficient knowledge, understanding and competences corresponding to position of professionals in the working place, including the type and level of formal education.

**Methods:** The goal of the Academic Master program for Radiopharmacy in English, with the recognized accreditation from the National Body in 2014 and recommended by the Ministry of Education and Science in our country according to the Bologna system with 120 ECTS credits is to give the opportunity for all students with finished academic undergraduate program (at least 240 ECTS) to follow it and to receive academic title Master of Radiopharmacy. The Radiopharmacy Education Program is organized according to the University model including Obligatory and Optional Theoretical Courses, Obligatory Practices and Master Thesis on the end of the second year using the University laboratory of Radiopharmacy, that is under ISO accreditation, new PET facilities and all other corresponding facilities available in the university and in the country. The program use e-learning platform from the University and IAEA contribution and collaboration related to the education and training of the students from developing countries. The result of that collaboration is already inscribed students from Kenya and Ethyopia.

**Results:** The syllabus include knowledge and skills in three categories: 1. Specific knowledge and skills:Design of Radiopharmacy facilities and QA program, Production and synthesis of radionuclides and radiopharmaceuticals, Quality control of radiopharmaceuticals 2. Basic knowledge and skills: Leadership and collaboration, Communication, Negotiations, Analytical and critical thinking skills including creativity and ethical considerations, Pharmaceutical and chemistry knowledge 3. Fundamentals of Radiopharmacy: General models of the area, Key specializations within the field, Evaluation of performance within the area.

**Conclusions:** The program creates academic staff able to work in all spheres of the modern Radiopharmacy, conventional hospital Radiopharmacy and with the option of continuing their education in some PhD program. The program can be also a basic platform for the more advanced courses available for the professionals from the European and world recognized associations.

### PP63 Synthesis and preclinical validations of a novel 18F-labelled RGD peptide prepared by ligation of a 2-cyanobenzothiazole with 1,2-aminothiol to image angiogenesis.

#### Didier J. Colin^1^, James A.H. Inkster^2^, Stéphane Germain^1^, Yann Seimbille^3^

##### ^1^MicroPET/SPECT/CT Imaging Laboratory, Centre for BioMedical Imaging (CIBM), University Hospital of Geneva, Geneva, Switzerland; ^2^Cycloton Unit, University Hospital of Geneva, Geneva, Switzerland; ^3^Life Sciences Division, TRIUMF, Vancouver, Canada

###### **Correspondence:** Yann Seimbille **–** MicroPET/SPECT/CT Imaging Laboratory, Centre for BioMedical Imaging (CIBM), University Hospital of Geneva, Geneva, Switzerland

**Introduction:** The RGD-recognising αVβ3, αVβ5 and α5β1 integrins are known to be involved in carcinogenesis and are overexpressed in many types of tumours compared to healthy tissues; thereby they have been selected as promising therapeutic targets. Positron emission tomography (PET) is providing a unique non-invasive screening assay to discriminate which patient is more prone to benefit from antiangiogenic therapies, and extensive research has been carried out to develop a clinical radiopharmaceutical that bind specifically to integrin receptors. However, despite promising clinical results, one still needs to identify an integrin radioligand that could be easily deployed. We herein report the synthesis of a 18F-labelled RGD peptide, namely [18F]FPyPEGCBT-c(RGDfK), by condensation of a 2-cyanobenzothiazole prosthetic group to a cysteine-modified cyclic RGD peptide and the characterisation of its preclinical biologic properties.

**Materials and methods:** A novel bifunctional synthon ([18F]FPyPEGCBT) has been developed to accommodate: i) an efficient [18F]F- incorporation and ii) a mild conjugation to biomolecules. Then, [18F]FPyPEGCBT has been conjugated to a cyclic RGD peptide known for its high binding affinity and selectivity for integrin αVβ3. The in vitro binding characteristics of FPyPEGCBT-c(RGDfK) were analysed by standard binding assay in U-87 MG and SKOV-3 cancer models and its selectivity towards integrins by siRNA depletions. Its preclinical potential was studied in mice bearing subcutaneous tumours by ex vivo biodistribution studies and in vivo microPET/CT imaging.

**Results:** [18F]FPyPEGCBT was prepared from its corresponding 2-trimethylammonium triflate precursor and purified by solid-phase extraction. Ligation of [18F]FPyPEGCBT to the cysteine-modified cyclo-(RGDfK) peptide was carried out in DMF at 43 oC. The 18F-peptide was obtained, after HPLC purification and reformulation, in 124–132 min from the end of bombardment with a final decay-corrected yield of 7% ± 1. In vitro, FPyPEGCBT-c(RGDfK) bound efficiently to RGD-recognising integrins as compared to a control c(RGDfV) peptide (IC50 = 30.8 × 10-7 M vs. 6.0 × 10-7 M). In vivo, this new tracer demonstrated specific tumour uptake with %ID/g of 2.9 and 2.4 in U-87 MG and SKOV-3 tumours 1 h after injection. Tumour-to-muscle ratios of 4 after 1 h of uptake allowed good visualisation of the tumours. However, unfavourable background accumulation and high hepatobiliary excretion were noticed.

**Discussion/conclusion: [**18F]FPyPEGCBT-c(RGDfK) specifically detects tumours expressing RGD-recognising integrin receptors in preclinical studies. Further optimisation of this radioligand may yield candidates with improved imaging properties and would warrant the further use of this efficient labelling technique for alternative targeting vectors.

